# Genetically predicted testosterone and cancers risk in men: a two-sample Mendelian randomization study

**DOI:** 10.1186/s12967-022-03783-z

**Published:** 2022-12-08

**Authors:** Junke Chang, Yongming Wu, Sicheng Zhou, Ye Tian, Yan Wang, Jie Tian, Wenpeng Song, Yinxian Dong, Jue Li, Ziyi Zhao, Guowei Che

**Affiliations:** 1 Department of Thoracic Surgery, West China Hospital, Sichuan University, Chengdu, China; 2 Department of Lung Cancer Center, West China Hospital, Sichuan University, Chengdu, China; 3 Healthy Food Evaluation Research Center, West China School of Public Health and West China Fourth Hospital, Sichuan University, Chengdu, China

**Keywords:** Testosterone, Bioavailable testosterone, Cancer, Mendelian randomization

## Abstract

**Objective:**

In observational studies, testosterone has been reported to be associated with some types of cancers. However, the direction and magnitude of the causal association between testosterone and different types of cancer remain unclear. This Mendelian randomization study assessed the causal associations of total testosterone (TT) and bioavailable testosterone (BT) with cancer risk in men.

**Methods:**

We performed two-sample Mendelian randomization using publicly available GWAS summary statistics to investigate the genetically causal association between testosterone and the risk of 22 kinds of cancers in men. Causal estimates were calculated by the inverse variance weighted method. We also performed additional sensitivity tests to evaluate the validity of the casualty.

**Results:**

Genetically predicted BT level were significantly associated with an increased risk of prostate cancer [odds ratio (OR) = 1.17 95% confidence interval (CI): 1.09–1.26, P = 2.51E^−05^] in the MR analysis with the IVW method. TT was found to be the suggestive protective factor against stomach cancer (OR = 0.66, 95% CI: 0.48–0.93, P = 0.0116) as well as pancreatic cancer (OR = 0.59, 95% CI: 0.36–0.96, P = 0.0346). A suggestive association was found between TT and the occurrence of small intestine cancer (OR = 1.0004, 95% CI: 1.0001–1.0007, P = 0.0116). However, testosterone had no significant association with other cancers.

**Conclusion:**

This study investigated the role of testosterone in the development of prostate cancer, stomach cancer, pancreatic cancer, and small intestine cancer but found no strong association with the other cancers in men.

**Supplementary Information:**

The online version contains supplementary material available at 10.1186/s12967-022-03783-z.

## Introduction

As a primary sexual hormone, testosterone not only plays a key role in the primary sexual development and regulation of secondary male characteristics but is also responsible for the metabolism of glucose/lipid/proteins, growth of muscle, and adipogenesis [1]. Testosterone has a variety of physiological target organs, such as the prostate, bone, muscle, brain, and peripheral nerves [2]. Testosterone deficiency may be associated with metabolic syndrome, obesity, sexual dysfunction, impaired infertility, depression, bone/muscle mass loss, anaemia, diabetes, and sarcopenia [3]. Testosterone is primarily bound to sex hormone binding protein (SHBG) and albumin and dissociate in tissue capillaries to generate free testosterone to exert androgenic activity [4]. Testosterone binding to albumin is weak and can be reversed easily. Therefore free testosterone and albumin-bound testosterone are called bioavailable testosterone. A cohort study showed that low serum testosterone can increase the risk of prostate cancer. Patients with baseline testosterone below 3.85 ng/ml had greater chances of developing prostate cancer than those with high levels [5]. However, whether testosterone levels are a risk factor or predictor of prostate cancer prognosis remains unclear. A case–control study suggested that high levels of circulating free testosterone may be associated with a reduced risk of lung cancer in postmenopausal never-smoking women [6]. Another study suggested that premenopausal women with elevated serum testosterone levels have an increased risk of breast cancer [7]. A meta-analyses demonstrated inverse associations between circulating levels of testosterone and colorectal/colon cancer risk [8]. Although there are many researches on the association of indirect measures of testosterone exposure with the risk of cancer, studies on the causal role of testosterone levels in men’s cancers remain unclear.

Mendelian randomization (MR) is a method of using genetic variation arising from meiosis to investigate the causal relationship between exposure and complex outcomes. MR analysis depends on integrating genome-wide association study (GWAS) summary data [9]. A genetic variable is valid in the MR analysis if it meets the following 3 assumptions: (i) the genetic variants are associated with exposure; (ii) the genetic variants are independent of confounders between exposure and outcomes, and (iii) the genetic variants only influence the outcome via exposure [10]. Two-sample MR analysis refers to an MR analysis that includes a pair of exposures and outcomes from different (nonoverlapping) samples of the same underlying population. Many researchers have used Mendelian randomization to explore the role of endogenous testosterone in different diseases. Testosterone was shown to have a positive correlation with thromboembolism, heart failure, and myocardial infarction in men through a Mendelian randomization study by Luo [11]. With genetic instruments from JMJD1C and SHBG regions, a two-sample Mendelian randomization study found that low levels of testosterone may cause gout and type II diabetes (T2D), while testosterone higher than normal levels may result in rheumatoid arthritis (RA) and depression [12]. With the rapid development of large-scale GWAS, many MR studies have emerged that have explored the potential causal relationship between exposure and cancers, for example, elevated plasma HDL and LDL levels have been associated with increased breast cancer risk [13] and type 2 diabetes mellitus (T2DM) have been associated with several cancers [14].

This study used large-scale GWAS data to assess the potential causal associations of testosterone levels with 22 kinds of cancers in men through a two-sample MR study. This study helps to reveal testosterone’s genetic role in men’s cancer risk.

## Methods

### Genetic instruments

The measurable testosterone includes TT and BT, both of which can reflect the effect of testosterone in the body. Up-to-date summary statistics for men’s TT and BT were obtained from the UKBiobank, conducted by Hayes et al. [15]. This study consists of a large-scale GWAS including TT and BT of males, which included 199,569 (TT) and 184,205 (BT) European individuals, respectively. The threshold for single nucleotide polymorphism (SNP) selection was P < 5 × 10^–8^. SNPs must be independent of each other, so clustering was performed to rule out linkage disequilibrium (LD) between SNPs. The criteria for LD were defined as SNPs with R2 > 0.01 and physical distance within 10,000 kb. Among SNP-shaved LDs, only those with the lowest p values were retained. Then we performed a look‐up of all of the SNPs in Phenoscanner (a curated database holding publicly available results from large‐scale GWAS with > 65 billion associations and > 150 million genetic variants) to evaluate whether these SNPs were associated with other traits at the genome-wide significance level (P < 5 × 10^–8^) that could be potential confounders [16]. In GWAS of TT, We found that nine SNPs (rs1730865, rs3842763, rs28929474, rs1933801, rs520829, rs55707100, rs59194935, rs79717793, and rs8107967) were associated with Body mass index (BMI), one SNP (rs4925809) was associated with years of educational attainment, and one SNP (rs40831) was associated with alcohol intake frequency. As for GWAS of BT, eight SNPs (rs9284814, rs1264327, rs1933801, rs1361109, rs62465144, rs11600831, rs1272131, rs2295094) were detected associated with BMI, one SNP (rs8061590) was associated with years of educational attainment and one SNP (rs58879558) was associated with alcohol intake frequency. After excluding 11 SNPs and 10 SNPs in TT and BT GWAS separately, we used the remaining SNPs as the instrument in the MR analysis (Additional file 1: Tables S2, S3).

### Data source of different types of cancer

The summary statistics from the GWAS for cancers in the publicly available databases were retrieved from the Medical Research Council Integrative Epidemiology Unit OpenGWAS project (https://gwas.mrcieu.ac.uk/), Medical Research Council (MRC), University of Bristol. The GWASs of cancers all included patients of European ancestry because two-sample MR requires data from the same population. Therefore, we excluded the GWAS of cancers from different populations. Supplementary Table S1 presents the summary of the data sources of the different traits, including the number of SNPs, number of cases, number of controls, sample size, etc. The GWAS data for colon, rectum, brain cancer, and melanoma were obtained from the MRC IEU OpenGWAS database [17]. The estimates for the association between the genetic variants and risk of kidney cancer, liver and intrahepatic bile duct cancer, lip, oral cavity, and pharynx cancer, stomach cancer, testis cancer, thyroid cancer, lung cancer, male genital organ cancer, multiple myeloma, and malignant plasma cell neoplasms were obtained from the publicly available summary statistics of the FinnGen consortium (www.finbb.fi). The GWAS summary data of prostate cancer, pancreatic cancer, lung cancer, small intestinal cancer, head, and neck cancer, and bladder cancer were obtained from the Prostate Cancer Association Group to Investigate Cancer Associated Alterations in the Genome (PRACTICAL), Pancreatic Cancer Cohort Consortium (PanScan), Neale Lab, and Genome-wide Association Study of Cancer Risk in UK Biobank, respectively[18–21]. Because we used only deidentified data, we did not need institutional review board approval for our analysis.

### Estimation of causal association

**Fig. 1:**
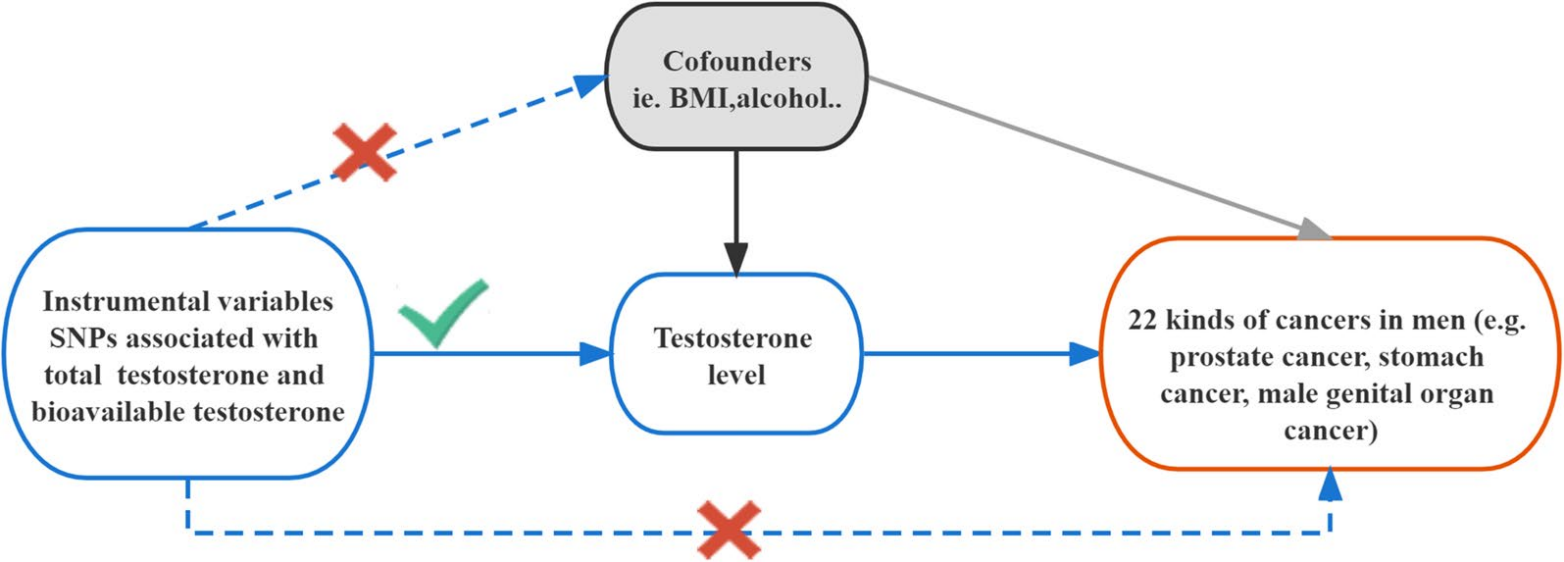
Study design of Mendelian randomization between testosterone and 22 kinds of cancers. The blue solid lines represent the association between the instrumental variables and exposure as well as the association between exposure and outcome. Dash lines with a cross means that the association meets two basic assumptions of Mendelian randomization: (i) the genetic variants are independent of confounders between exposure and outcomes, (ii) the genetic variants only influence the outcome via exposure

After clustering, only the SNPs with MAF > 0.01 within the European population were analysed in the exposure data. We also harmonized the exposure and outcome data to ensure that the exposure and outcome data were reconciled to ensure SNP effects on the same allele. Then, we estimated the association between testosterone and each kind of cancer with several two-sample MR methods, including inverse-variance weighted (IVW), MR Egger, and weighted median. The IVW method is the most efficient MR method, but it relies on the assumption that all genetic variants are valid instrumental variables. The weighted median estimator allows up to half of the SNPs not to be instrumental variables (IVs), and this method can assess whether SNPs have pleiotropic effects on the outcome [22]. To further test the robustness of causal association, a series of sensitivity analyses, such as the heterogeneity test by Cochran’s Q test and I^2^ statistics (a p-value of < 0.05 or an I^2^ value of > 50%), MR presso, the pleiotropy test, and the leave-one-out test, were employed. In addition, we used the ggplot2 package to visualize the results using scatter plots, forest plots, least-one-out plots, and funnel plots. All the analyses mentioned were performed with R software (v4.1.3). We used the two-sample MR R package (v0.5.6) to perform the MR analysis. Figure 1 presents the workflow of our study. A Bonferroni-corrected significance threshold of P = 1.1364 × 10^–3^ (0.05/44 [2 exposures and 22 outcomes]) was prespecified to adjust for multiple testing. Associations with p values between 0.05 and 1.1364 × 10^–3^ were considered suggestive evidence of a possible association.

## Results

### Causal effect of testosterone on cancer

**Fig. 2:**
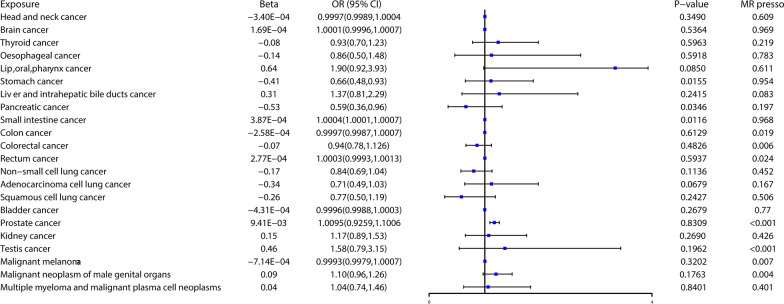
Associations of total testosterone levels with 22 kinds of cancer in the MRC IEU, FinnGen, PRACTICAL, PanScan, Neale Lab, UK Biobank. There were suggestive association between TT level and stomach cancer, small intestine cancer, and pancreatic cancer. Beta: beta coefficient, OR, odds ratio

**Fig 3:**
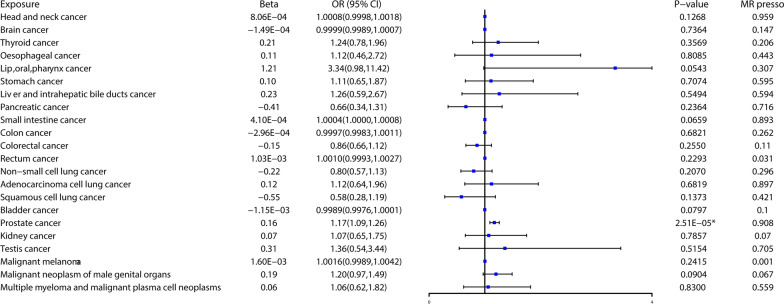
Associations of bioavailable testosterone levels with 22 kinds of cancer in the MRC IEU, FinnGen, PRACTICAL, PanScan, Neale Lab, UK Biobank. *MR presso detected 4 distortion SNPs (rs10279715, rs11191415, rs13072540, and rs34040779) between BT and prostate cancer, the results of MR analysis showed in this figure were tested after excluding these SNPs. The results showed that BT level was significantly associated with prostate cancer. Beta: beta coefficient, OR, odds ratio

There were 126 and 55 candidate IVs for TT and BT after P value selection and LD clumping, respectively (Additional file 1: Tables S2, S3). We presented the results of the MR analysis of the causal effect of testosterone on the different types of cancer in Figs. 2, 3 and Additional file 1: Tables S4, S5. After excluding distortion SNPs, genetically predicted BT levels were significantly associated with an increased risk of prostate cancer (OR = 1.17, 95% CI: 1.09–1.26, P = 2.51E^−05^) in the MR analysis with the IVW method. TT was found to be the suggestive protective factor against stomach cancer (OR = 0.66, 95% CI: 0.48–0.93, P = 0.0116) as well as pancreatic cancer (OR = 0.59, 95% CI: 0.36–0.96, P = 0.0346). TT level was suggestively associated with an increased risk of small intestine cancer (OR = 1.0004, 95% CI: 1.0001–1.0007, P = 0.0116). However, TT and BT had no association with the rest kinds of cancers (Figs. 1, 2).

### Sensitivity analysis

A sensitivity analysis was conducted to verify the reliability of IVW results. IVW and MR-Egger tests for heterogeneity showed that there was no heterogeneity in MR analysis results between TT and stomach and pancreatic cancer as well as BT and prostate cancer (P > 0.05) (Additional file 1: Table S6). The MR-Presso global test was also applied and didn’t show any heterogeneity in the combination above. After removing these SNPs, TT and prostate cancer showed a casual association though still had heterogeneity. After removing distortion SNPs (rs10279715, rs11191415, rs13072540, and rs34040779) detected by MR presso, BT and prostate cancer showed a significant association and had no heterogeneity.

Except for TT with small intestine cancer, MR-Egger regression results showed that there was no pleiotropy in MR analysis results (P > 0.05) (Additional file 1: Table S7). Scatter plots and funnel plots of each pair of associations for casualty were shown in the supplementary materials (Additional file 2: Figures S1–S4). The result of the Leave-one-SNP-out analysis indicated that there weren’t significant impact on the results (Additional file 2: Figures S5–S50).

## Discussion

This is the first two-sample MR analysis of the potential causality of men’s testosterone levels for a broad range of site-specific cancers. The results showed that BT level was significantly associated with a higher risk of prostate cancer; TT levels may be a suggestively protective factor for stomach cancer and pancreatic cancer. There was a suggestive association between TT level and increased risk of small intestine cancer as. We did not find significant evidence of the association of testosterone levels with other cancers.

Bioavailable testosterone is the part of testosterone that diffuses easily and plays important roles in different organs and systems. So it can better reflect the biological activity of testosterone than TT. As has been previously mentioned, testosterone, and testosterone deficiency has a significant impact on the overall quality of life. In the 1940s, Huggins and Hodges found that orchiectomy or estrogen therapy for metastatic prostate cancer patients can reduce testosterone secretion and reduce serum acid phosphatase activity and injection of androgens can increase acid phosphatase. Since then, testosterone was believed to promote prostate cancer cell growth and disease progression [23]. Subsequently, more evidence claimed high serum testosterone does not increase the risk of prostate cancer [24]. Prostate growth is very sensitive to changes in androgen concentrations that are well below physiological concentrations but is not sensitive to higher levels of androgen concentrations due to receptor saturation [25]. A review that included 25 studies found that higher serum testosterone levels were associated with a lower risk of prostate cancer [2]. A in vitro study showed that low levels of androgens are essential for the growth of prostate cancer cells, while physiological levels or higher inhibit the growth of prostate cancer cells, which may explain the contradiction between androgen deprivation therapy for prostate cancer and the reduced risk of prostate cancer in patients with high testosterone levels [26]. Therefore, whether testosterone can promote the occurrence and development of prostate cancer is a paradox. Some scholars have proposed a model to explain it: If levels of testosterone below the saturation point, prostate cancer growth would be expected to vary with testosterone concentration. But Exogenous testosterone couldn’t cause any increase of prostate volume or development of prostate cancer in normal men [27, 28]. Overall, the relationship between testosterone and prostate cancer is complex, and our study supports the hypothesis that testosterone is a risk factor for prostate cancer at the genetic level.

Few studies have investigated sex hormones and gastrointestinal cancer risk. But testosterone has shown different roles in the development of gastrointestinal cancers in existing studies. A large prospective cohort study revealed that testosterone had little association with colorectal cancer and oesophageal squamous cell carcinoma in men [29]. Our MR study also did not support an association between testosterone and colorectal cancer risk in men. As for stomach cancer, our results suggested a positive association between testosterone and stomach cancer, though a case–control study by Yelda et al. found levels of testosterone were not associated with stomach cancer [30]. The precise role of testosterone in the stomach is not well understood.

We suspect this may be due to the anti-inflammatory effects of testosterone. It was reported by Jonathan et al. that androgens treatment could suppress the expression of the proinflammatory cytokines by ILC2s. To protect the stomach from spasmolytic polypeptide-expressing metaplasia (SPEM) development [31]. Chronic SPEM is associated with the development of gastric adenocarcinoma [32]. The study by Busada et al. also implicated androgens limited IL-33-driven lung inflammation through a cell-intrinsic inhibition of ILC2 expansion. It could well explain why men have lower rates of inflammatory and autoimmune diseases than women. However, Men are twice to third as likely to develop stomach cancer as women, suggesting other factors contribute to disease risk [33]. So whether testosterone plays a role in the prevention of stomach cancer merits further study. In our study, testosterone might be a risk factor for small intestine cancer. To date, there are few studies on the association between testosterone and small intestine cancer.

Epidemiological studies have shown that men are more likely to develop pancreatic cancer than women, with a male-to-female ratio between 1.25–1.75 and 1, suggesting that sex hormones may play a role in pancreatic cancer [34]. The presence of androgen receptors in human pancreatic tissue is also well established, and some animal experiments have confirmed that testosterone promotes the growth of pancreatic cancer cells [35–37]. However, none of the several observational studies were able to conclude that testosterone levels are higher in pancreatic cancer patients, and even in a considerable number of studies, testosterone levels were found to be lower in pancreatic cancer patients [38–40]. Moreover, some the other experiments have also shown that dehydroepiandrosterone (DHEA), an androgen precursor, inhibits pancreatic cancer in animal models [41, 42]. Given the possible effects of androgens on pancreatic cancer, several prospective randomized controlled trials have explored the effects of androgen receptor inhibitors on pancreatic cancer; however, the findings have been inconsistent y[43, 44]. In addition to their methodological shortcomings, the sample sizes of these RCTs were small, making it difficult to draw reliable conclusions. While our study suggested that a high level of testosterone is a protective factor against pancreatic cancer, more studies are needed in the future to reveal the exact mechanisms.

The main strength of this study is the MR design, which reduces potential bias from confounding factors and reverses causality. Another important advantage is that we assessed the association between testosterone and a wide range of cancers, most of which were not previously examined based on genetic instruments. One limitation is that our analysis included only men of European ancestry, thus limiting the universality of our results to other populations. Another disadvantage is that it was less accurate in analyzing cancers with a limited number of cases (fewer than 1000). More large sample data needs to be included for validation analysis. Finally, we cannot pinpoint the relationship between specific serum testosterone levels and various cancers, which will be fulfilled in later studies.

## Conclusions

In summary, the results of this study suggested a significant causal between genetically predicted testosterone levels and the risk of prostate cancer. Testosterone was also suggestively associated with the risk of stomach cancer, pancreatic cancer, and small intestine cancer among different types of cancer. These findings indicate the importance of testosterone in the prevention and treatment of cancer. To better illustrate the causal association between testosterone and 22 kinds of cancer, more large-scale prospective studies and mechanistic studies are warranted.

## Supplementary Information

**Additional file 1:** **Table S1**. Summary of data source of different Traits; **Table S2**. The single nucleotide polymorphism selected for total testosterone to perform Mendelian randomization analysis; **Table S3**. The single nucleotide polymorphism selected for available testosterone to perform Mendelian randomization analysis; **Table S4**. Estimates of causal effect of total testosterone on different types of cancer; **Table S5**. Estimates of causal effect of bioavailable testosterone on different types of cancer; **Table S6**. Heterogeneity analysis of mendelian randomization analysis of testosterone on different types of cancer; **Table S7**. Pleiotropy analysis of mendelian randomization analysis of testosterone on different types of cancer; **Table S8**. Leave-one-out analysis of mendelian randomization analysis of total testosterone on different types of cancer; **Table S9**. Leave-one-out analysis of mendelian randomization analysis of bioavailable testosterone on different types of cancer.

**Additional file 2:** **Figure S1**. Scatter plot of the causal effect of total testosterone on different types of cancer; **Figure S2**. Funnel plot of the causal effect of total testosterone on different types of cancer; **Figure S3**. Scatter plot of the causal effect of bioavailable testosterone on different types of Cancer; **Figure S4**. Funnel plot of the causal effect of bioavailable testosterone on different types of cancer; **Figure S5-S50**. Leave-one-out inverse-variance weighted mendelian randomization analyses of total testosterone on different types of Cancer.

## Data Availability

All data generated or analyzed during this study are included in this article and its additional materials.
