# Supplementary material for: Genetically predicted testosterone and cancers risk in men: a two-sample Mendelian randomization study
Source: J Transl Med. 2022 Dec 8;20:573. doi: 10.1186/s12967-022-03783-z (PMC9730605; doi:10.1186/s12967-022-03783-z)
Supplement: Supplementary file 2 — Additional file 2: Figure S1. Scatter plot of the causal effect of total testosterone on different types of cancer; Figure S2. Funnel plot of the causal effect of total testosterone on different types of cancer; Figure S3. Scatter plot of the causal effect of bioavailable testosterone on different types of Cancer; Figure S4. Funnel plot of the causal effect of bioavailable testosterone on different types of cancer; Figure S5-S50. Leave-one-out inverse-variance weighted mendelian randomization analyses of total testosterone on different types of Cancer. [file 12967_2022_3783_MOESM2_ESM.pdf]

Supplementary Figures

Figure S1:Scatter plot of the causal effect of total testosterone on different types of cancer, with the slope of each line corresponding to the estimated causal effect per method 4

Figure S2:Funnel plot of the causal effect of total testosterone on different types of cancer 9

Figure S3:Scatter plot of the causal effect of bioavailable testosterone on different types of cancer, with the slope of each line corresponding to the estimated causal effect per method. 12

Figure S4:Funnel plot of the causal effect of bioavailable testosterone on different types of cancer 15

Figure S5:Leave-one-out inverse-variance weighted mendelian randomization analyses of total testosterone on Head and neck cancer 16

Figure S6:Leave-one-out inverse-variance weighted mendelian randomization analyses of total testosterone on Brain cancer 17

Figure S8:Leave-one-out inverse-variance weighted mendelian randomization analyses of total testosterone on Thyroid cancer 18

Figure S9:Leave-one-out inverse-variance weighted mendelian randomization analyses of total testosterone on Oesophageal cancer 20

Figure S10:Leave-one-out inverse-variance weighted mendelian randomization analyses of total testosterone on Lip, oral, and pharynx cancer 21

Figure S11:Leave-one-out inverse-variance weighted mendelian randomization analyses of total testosterone on Stomach cancer 22

Figure S12:Leave-one-out inverse-variance weighted mendelian randomization analyses of total testosterone on Liver and intrahepatic bile ducts cancer 23

Figure S13:Leave-one-out inverse-variance weighted mendelian randomization analyses of total testosterone on Pancreatic cancer 24

Figure S14:Leave-one-out inverse-variance weighted mendelian randomization analyses of total testosterone on Small intestine cancer 25

Figure S15:Leave-one-out inverse-variance weighted mendelian randomization analyses of total testosterone on Colon cancer 26

Figure S16:Leave-one-out inverse-variance weighted mendelian randomization analyses of total testosterone on Colorectal cancer 27

Figure S17:Leave-one-out inverse-variance weighted mendelian randomization analyses of total testosterone on Rectum cancer 28

Figure S18:Leave-one-out inverse-variance weighted mendelian randomization analyses of total testosterone on Non-small cell lung cancer 29

Figure S19:Leave-one-out inverse-variance weighted mendelian randomization analyses of total testosterone on Adenocarcinoma cell lung cancer 30

Figure S20:Leave-one-out inverse-variance weighted mendelian randomization analyses of total testosterone on Squamous cell lung cancer 31

Figure S21:Leave-one-out inverse-variance weighted mendelian randomization analyses of total testosterone on Bladder cancer 32

Figure S22:Leave-one-out inverse-variance weighted mendelian randomization analyses of total testosterone on Prostate cancer 33

Figure S23:Leave-one-out inverse-variance weighted mendelian randomization analyses of total testosterone on Kidney cancer 34

Figure S24:Leave-one-out inverse-variance weighted mendelian randomization analyses of total testosterone on Testis cancer 35

Figure S25:Leave-one-out inverse-variance weighted mendelian randomization analyses of total testosterone on Malignant melanoma 36

Figure S26:Leave-one-out inverse-variance weighted mendelian randomization analyses of total testosterone on Malignant neoplasm of male genital organs 37

Figure S27:Leave-one-out inverse-variance weighted mendelian randomization analyses of total testosterone on Multiple myeloma and malignant plasma cell neoplasms 38

Figure S28:Leave-one-out inverse-variance weighted mendelian randomization analyses of bioavailable testosterone on Head and neck cancer 39

Figure S29:Leave-one-out inverse-variance weighted mendelian randomization analyses of bioavailable testosterone on Brain cancer 40

Figure S31:Leave-one-out inverse-variance weighted mendelian randomization analyses of bioavailable testosterone on Thyroid cancer 41

Figure S32:Leave-one-out inverse-variance weighted mendelian randomization analyses of bioavailable testosterone on Oesophageal cancer 42

Figure S33:Leave-one-out inverse-variance weighted mendelian randomization analyses of bioavailable testosterone on Lip, oral, and pharynx cancer 43

Figure S34:Leave-one-out inverse-variance weighted mendelian randomization analyses of bioavailable testosterone on Stomach cancer 44

Figure S35:Leave-one-out inverse-variance weighted mendelian randomization analyses of bioavailable testosterone on Liver and intrahepatic bile ducts cancer 45

Figure S36:Leave-one-out inverse-variance weighted mendelian randomization analyses of bioavailable testosterone on Pancreatic cancer 46

Figure S37:Leave-one-out inverse-variance weighted mendelian randomization analyses of bioavailable testosterone on Small intestine cancer 47

Figure S38:Leave-one-out inverse-variance weighted mendelian randomization analyses of bioavailable testosterone on Colon cancer 48

Figure S39:Leave-one-out inverse-variance weighted mendelian randomization analyses of bioavailable testosterone on Colorectal cancer 49

Figure S40:Leave-one-out inverse-variance weighted mendelian randomization analyses of bioavailable testosterone on Rectum cancer 50

Figure S41:Leave-one-out inverse-variance weighted mendelian randomization analyses of bioavailable testosterone on Non-small cell lung cancer 51

Figure S42:Leave-one-out inverse-variance weighted mendelian randomization analyses of bioavailable testosterone on Adenocarcinoma cell lung cancer 52

Figure S43:Leave-one-out inverse-variance weighted mendelian randomization analyses of bioavailable testosterone on Squamous cell lung cancer 53

Figure S44:Leave-one-out inverse-variance weighted mendelian randomization analyses of bioavailable testosterone on Bladder cancer 54

Figure S45:Leave-one-out inverse-variance weighted mendelian randomization analyses of bioavailable testosterone on Prostate cancer 55

Figure S46:Leave-one-out inverse-variance weighted mendelian randomization analyses of bioavailable testosterone on Kidney cancer 56

Figure S47:Leave-one-out inverse-variance weighted mendelian randomization analyses of bioavailable testosterone on Testis cancer 57

Figure S48:Leave-one-out inverse-variance weighted mendelian randomization analyses of bioavailable testosterone on Malignant melanoma 58

Figure S49:Leave-one-out inverse-variance weighted mendelian randomization analyses of bioavailable testosterone on Malignant neoplasm of male genital organs 59

Figure S50:Leave-one-out inverse-variance weighted mendelian randomization analyses of bioavailable testosterone on Multiple myeloma and malignant plasma cell neoplasms 60

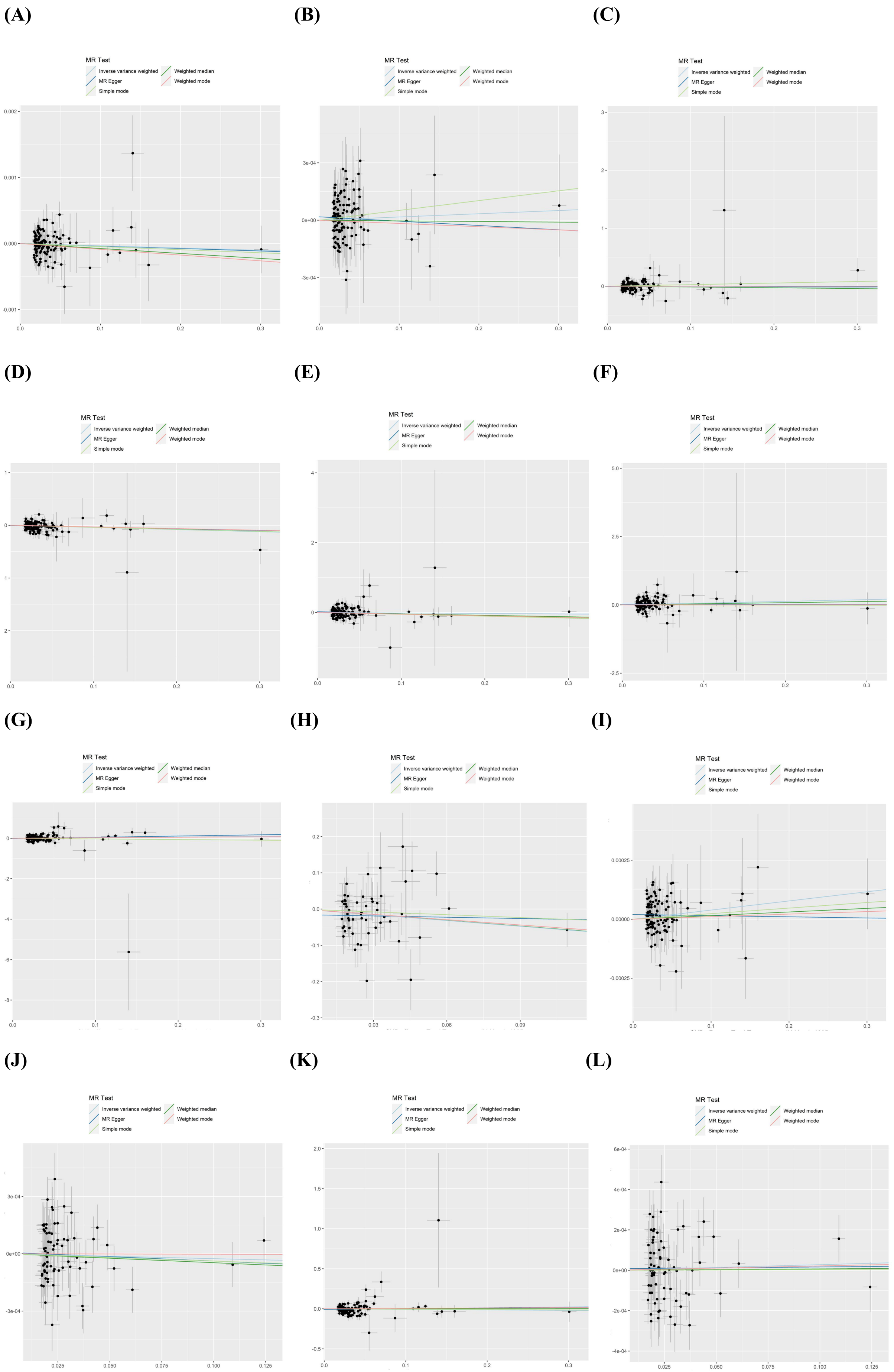
(A)
MR Test
Inverse variance weighted
MR Egger
Simple mode
Weighted median
Weighted mode
(B)
MR Test
Inverse variance weighted
MR Egger
Simple mode
Weighted median
Weighted mode
(C)
MR Test
Inverse variance weighted
MR Egger
Simple mode
Weighted median
Weighted mode
(D)
MR Test
Inverse variance weighted
MR Egger
Simple mode
Weighted median
Weighted mode
(E)
MR Test
Inverse variance weighted
MR Egger
Simple mode
Weighted median
Weighted mode
(F)
MR Test
Inverse variance weighted
MR Egger
Simple mode
Weighted median
Weighted mode
(G)
MR Test
Inverse variance weighted
MR Egger
Simple mode
Weighted median
Weighted mode
(H)
MR Test
Inverse variance weighted
MR Egger
Simple mode
Weighted median
Weighted mode
(I)
MR Test
Inverse variance weighted
MR Egger
Simple mode
Weighted median
Weighted mode
(J)
MR Test
Inverse variance weighted
MR Egger
Simple mode
Weighted median
Weighted mode
(K)
MR Test
Inverse variance weighted
MR Egger
Simple mode
Weighted median
Weighted mode
(L)
MR Test
Inverse variance weighted
MR Egger
Simple mode
Weighted median
Weighted mode

(M)

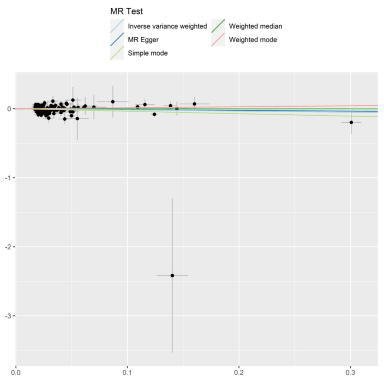
MR Test
Inverse variance weighted
Weighted median
MR Egger
Weighted mode
Simple mode

(N)

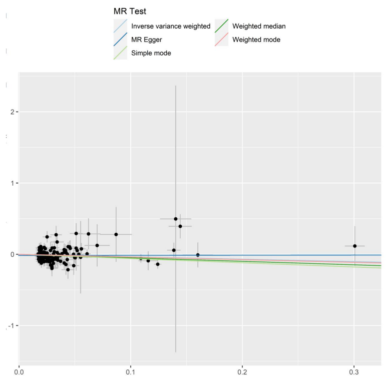
MR Test
Inverse variance weighted
Weighted median
MR Egger
Weighted mode
Simple mode

(O)

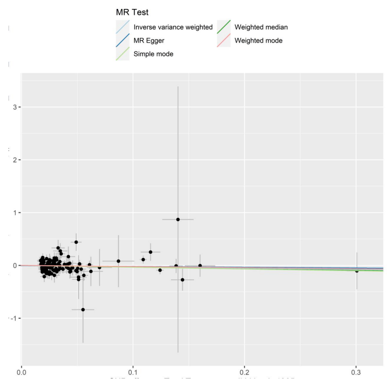
MR Test
Inverse variance weighted
Weighted median
MR Egger
Weighted mode
Simple mode

(P)

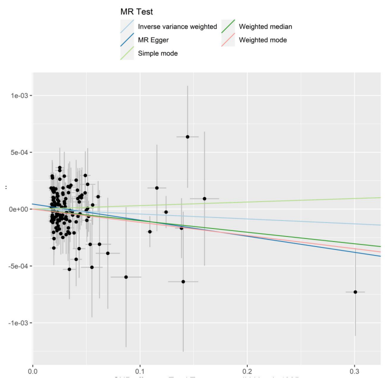
MR Test
Inverse variance weighted
Weighted median
MR Egger
Weighted mode
Simple mode

(Q)

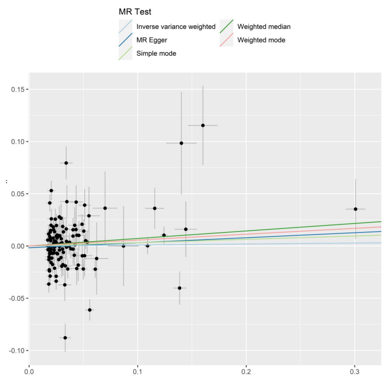
MR Test
Inverse variance weighted
Weighted median
MR Egger
Weighted mode
Simple mode

(R)

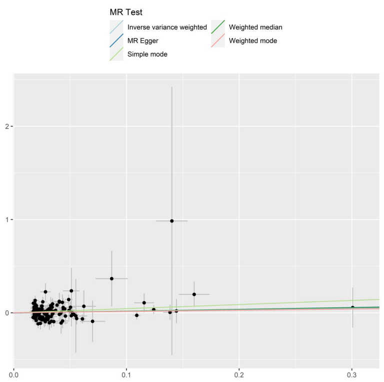
MR Test
Inverse variance weighted
Weighted median
MR Egger
Weighted mode
Simple mode

(S)

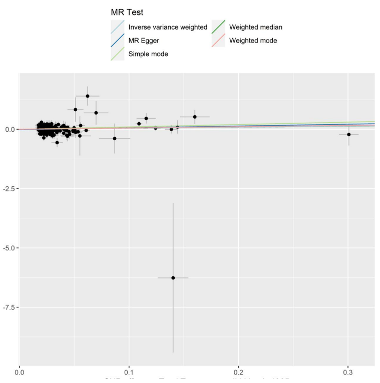
MR Test
Inverse variance weighted
Weighted median
MR Egger
Weighted mode
Simple mode

(T)

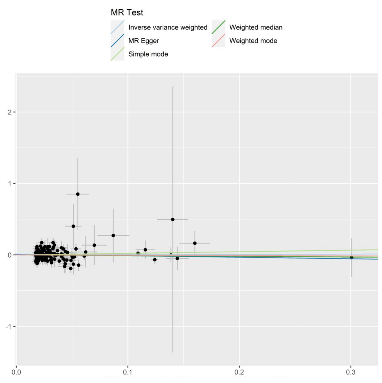
MR Test
Inverse variance weighted
Weighted median
MR Egger
Weighted mode
Simple mode

(U)

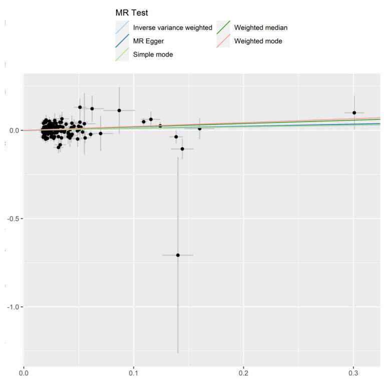
MR Test
Inverse variance weighted
Weighted median
MR Egger
Weighted mode
Simple mode

(V)

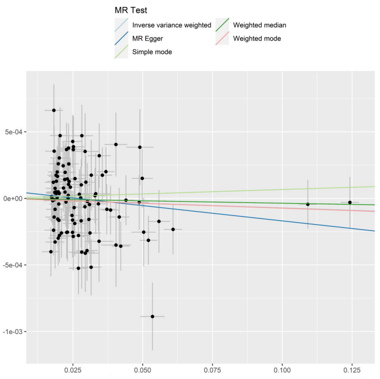
MR Test
Inverse variance weighted
Weighted median
MR Egger
Weighted mode
Simple mode

Figure S1:Scatter plot of the causal effect of total testosterone on different types of cancer, with the slope of each line corresponding to the estimated causal effect per method: (A) Head and neck cancer; (B) Brain cancer; (C) Thyroid cancer; (D) Oesophageal cancer; (E) Lip,oral, pharynx cancer; (F) Stomach cancer; (G) Liver and intrahepatic bile ducts cancer; (H) Pancreatic cancer; (I) Small intestine cancer; (J) Colon cancer; (K) Colorectal cancer; (L) Rectum cancer; (M) Non-small cell lung cancer; (N) Adenocarcinoma cell lung cancer; (O) Squamous cell lung cancer; (P) Bladder cancer; (Q) Prostate cancer; (R) Kidney cancer; (S) Testis cancer; (T) Malignant melanoma; (U) Malignant neoplasm of male genital organs; (V) Multiple myeloma and malignant plasma cell neoplasms.

(A) (B) (C)

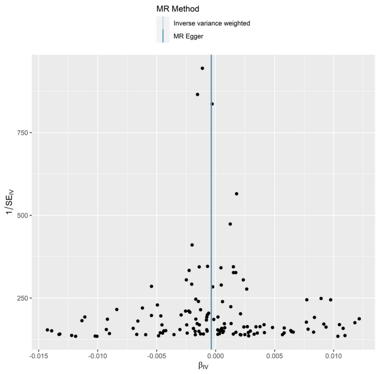

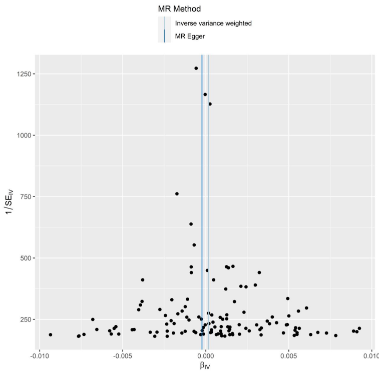

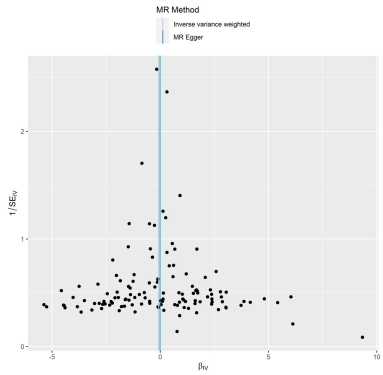

(D) (E) (F)

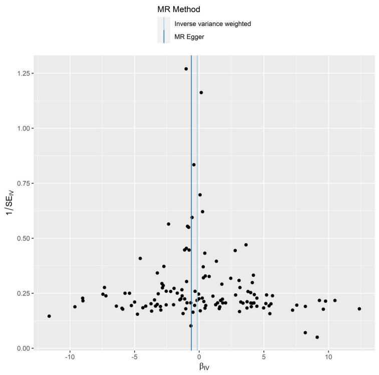

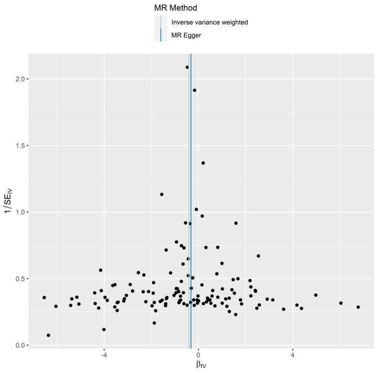

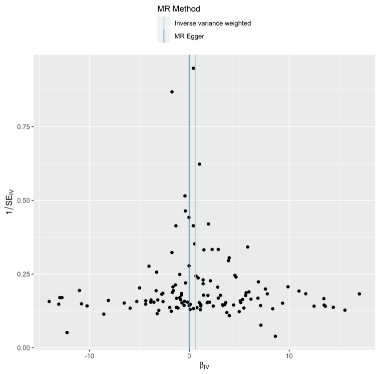

(G) (H) (I)

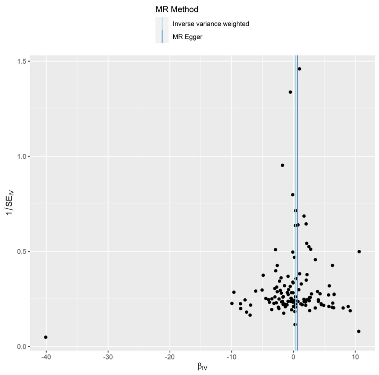

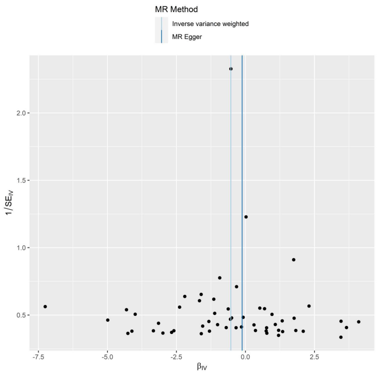

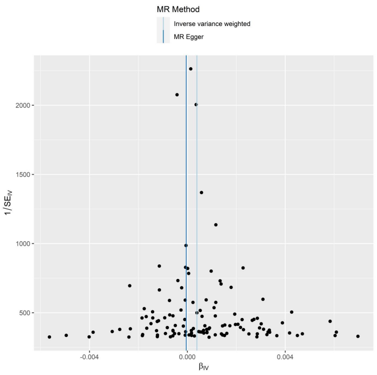

(J) (K) (L)

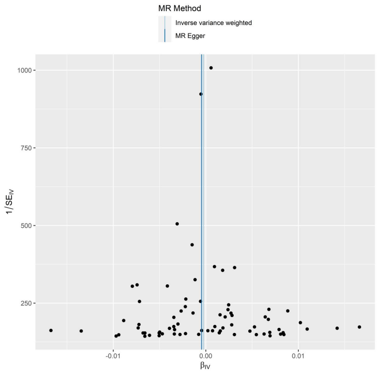

MR Method
Inverse variance weighted
MR Egger
1/SE$_{IV}$
β_{IV}

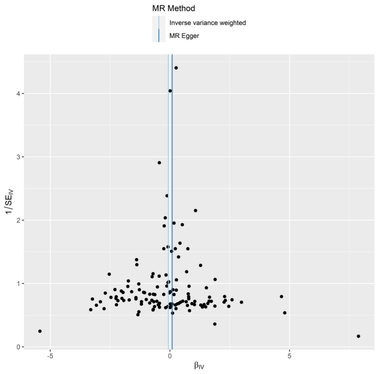

MR Method
Inverse variance weighted
MR Egger
1/SE$_{IV}$
β_{IV}

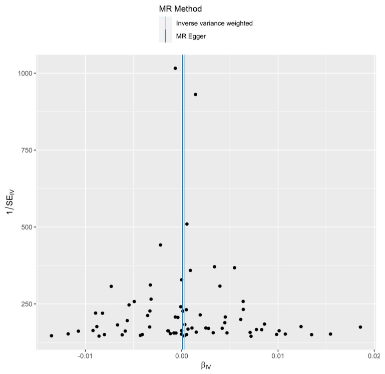

MR Method
Inverse variance weighted
MR Egger
1/SE$_{IV}$
β_{IV}

(M) **(N)** **(O)**

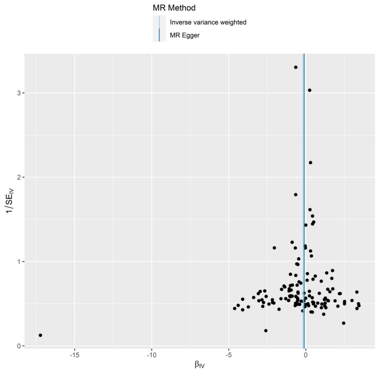

MR Method
Inverse variance weighted
MR Egger
1/SE$_{IV}$
β_{IV}

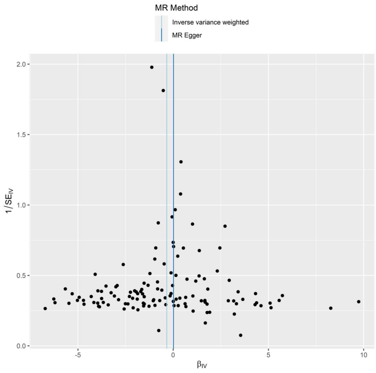

MR Method
Inverse variance weighted
MR Egger
1/SE$_{IV}$
β_{IV}

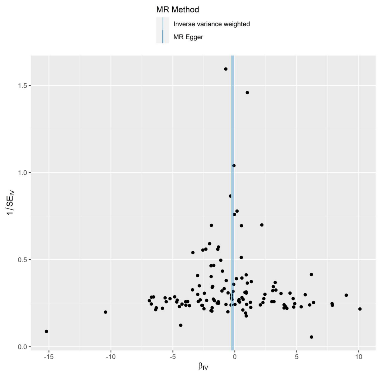

MR Method
Inverse variance weighted
MR Egger
1/SE$_{IV}$
β_{IV}

(P) **(Q)** **(R)**

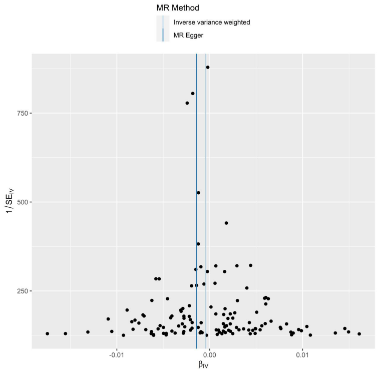

MR Method
Inverse variance weighted
MR Egger
1/SE$_{IV}$
β_{IV}

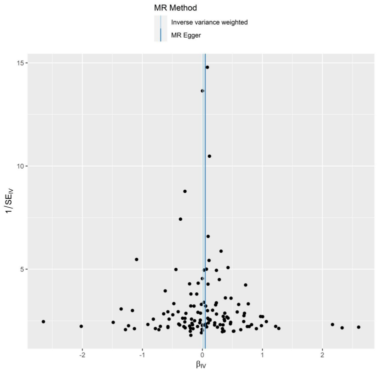

MR Method
Inverse variance weighted
MR Egger
1/SE$_{IV}$
β_{IV}

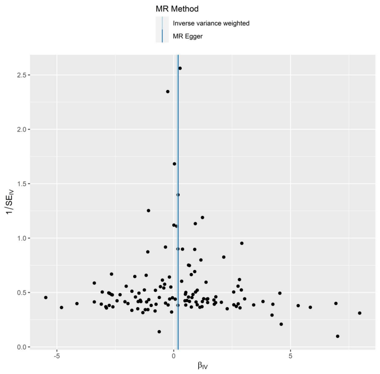

MR Method
Inverse variance weighted
MR Egger
1/SE$_{IV}$
β_{IV}

(S) **(T)** **(U)**

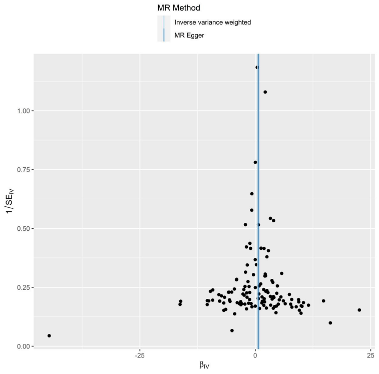

MR Method
Inverse variance weighted
MR Egger
1/SE$_{IV}$
β_{IV}

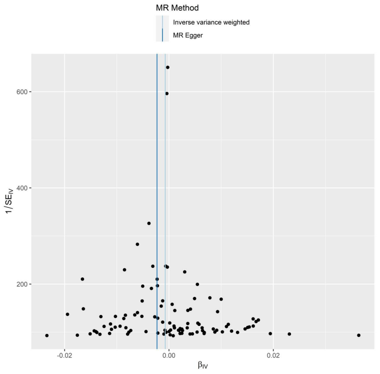

MR Method
Inverse variance weighted
MR Egger
1/SE$_{IV}$
β_{IV}

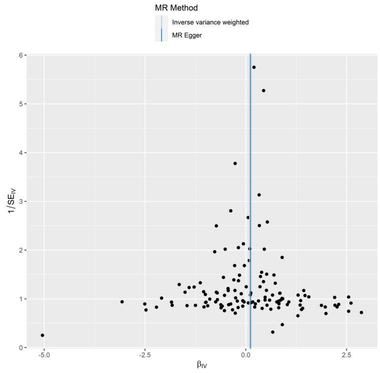

MR Method
Inverse variance weighted
MR Egger
1/SE$_{IV}$
β_{IV}

(V)

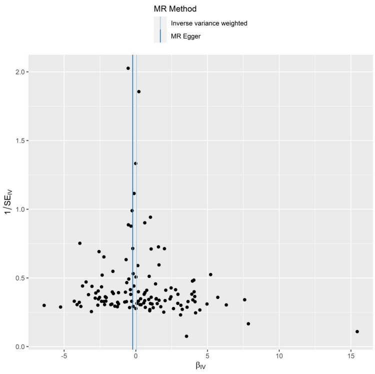

Figure S2:Funnel plot of the causal effect of total testosterone on different types of cancer: (A) Head and neck cancer; (B) Brain cancer; (C) Thyroid cancer; (D) Oesophageal cancer; (E) Lip, oral,pharynx cancer; (F) Stomach cancer; (G) Liver and intrahepatic bile ducts cancer; (H) Pancreatic cancer; (I) Small intestine cancer; (J) Colon cancer; (K) Colorectal cancer; (L) Rectum cancer; (M) Non-small cell lung cancer; (N) Adenocarcinoma cell lung cancer; (O) Squamous cell lung cancer; (P) Bladder cancer; (Q) Prostate cancer; (R) Kidney cancer; (S) Testis cancer; (T) Malignant melanoma; (U) Malignant neoplasm of male genital organs; (V) Multiple myeloma and malignant plasma cell neoplasms.

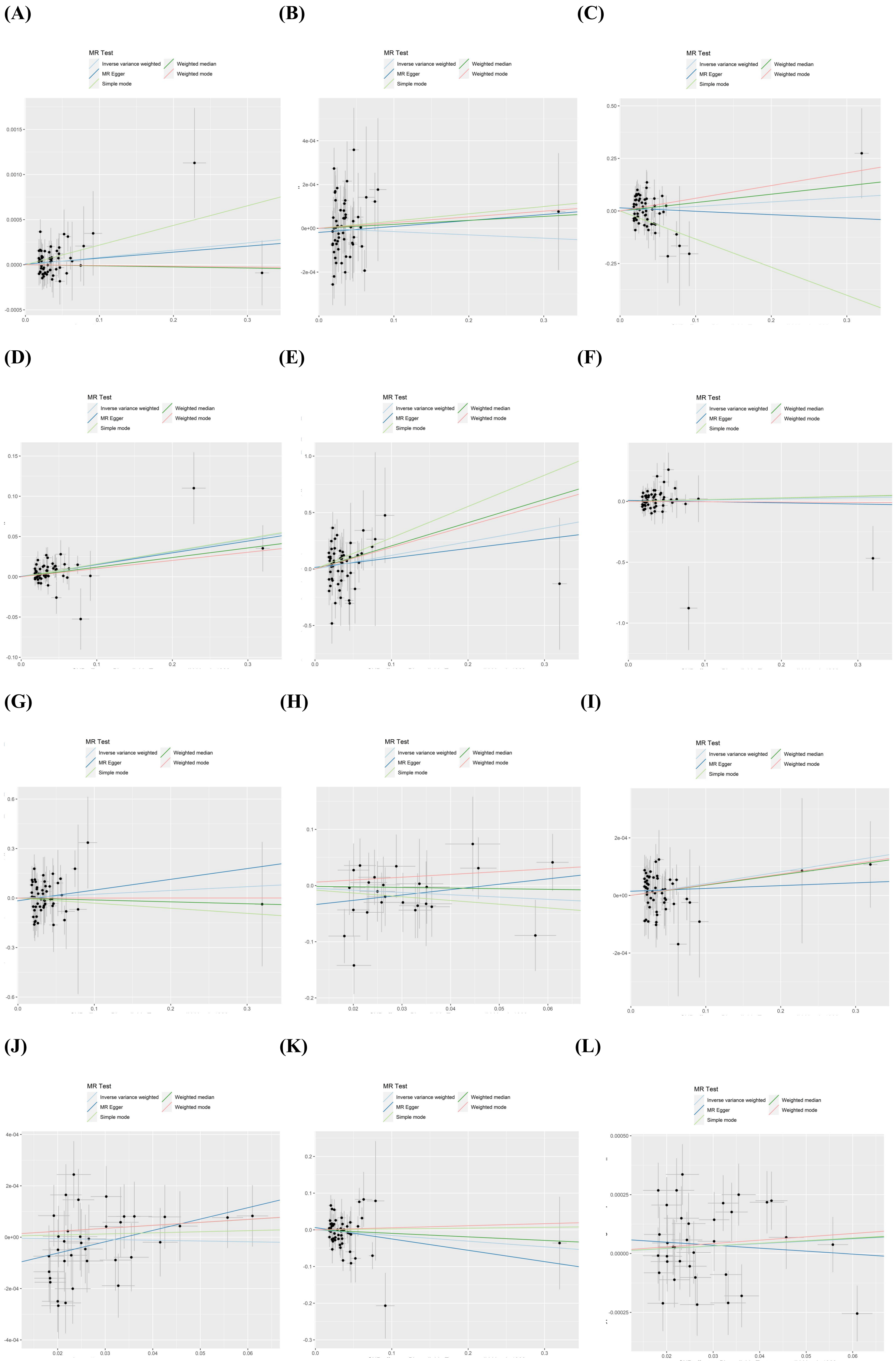
(A)
(B)
(C)
(D)
(E)
(F)
(G)
(H)
(I)
(J)
(K)
(L)
MR Test
Inverse variance weighted
MR Egger
Simple mode
Weighted median
Weighted mode

(M)

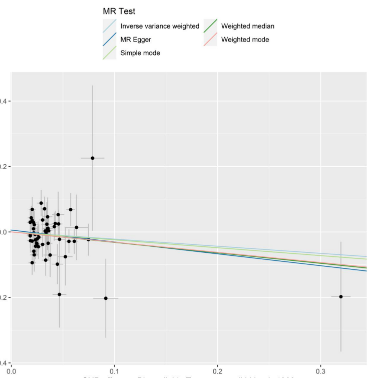
MR Test
Inverse variance weighted
MR Egger
Simple mode
Weighted median
Weighted mode

(N)

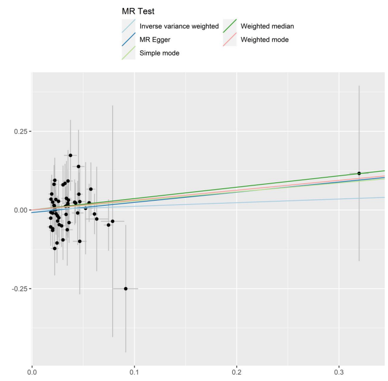
MR Test
Inverse variance weighted
MR Egger
Simple mode
Weighted median
Weighted mode

(O)

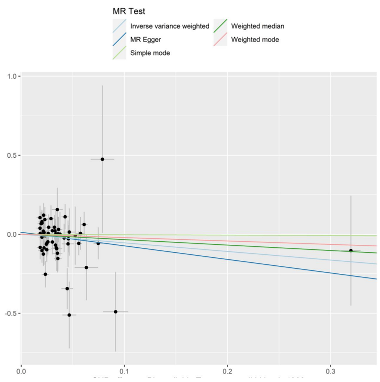
MR Test
Inverse variance weighted
MR Egger
Simple mode
Weighted median
Weighted mode

(P)

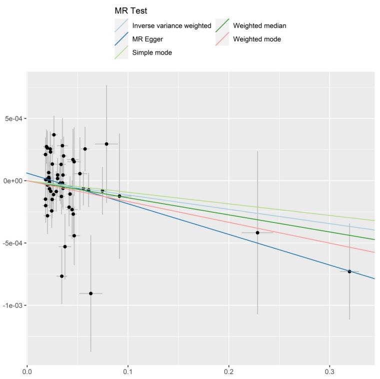
MR Test
Inverse variance weighted
MR Egger
Simple mode
Weighted median
Weighted mode

(Q)

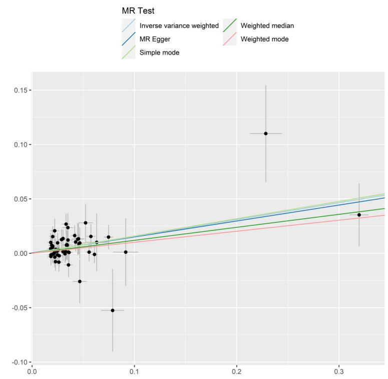
MR Test
Inverse variance weighted
MR Egger
Simple mode
Weighted median
Weighted mode

(R)

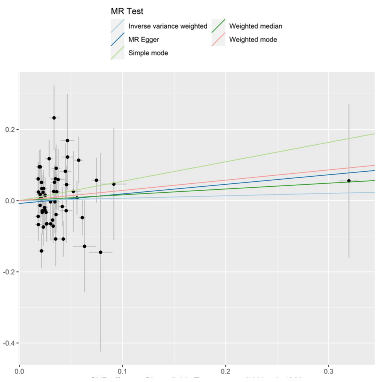
MR Test
Inverse variance weighted
MR Egger
Simple mode
Weighted median
Weighted mode

(S)

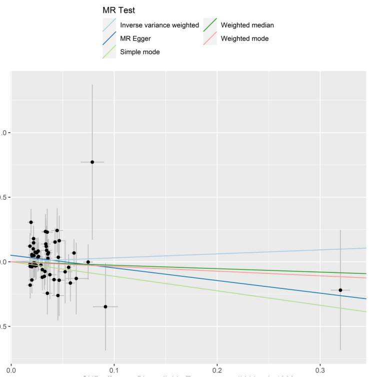
MR Test
Inverse variance weighted
MR Egger
Simple mode
Weighted median
Weighted mode

(T)

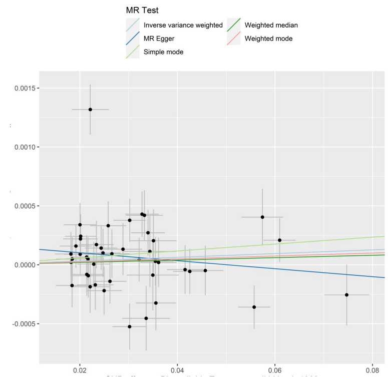
MR Test
Inverse variance weighted
MR Egger
Simple mode
Weighted median
Weighted mode

(U)

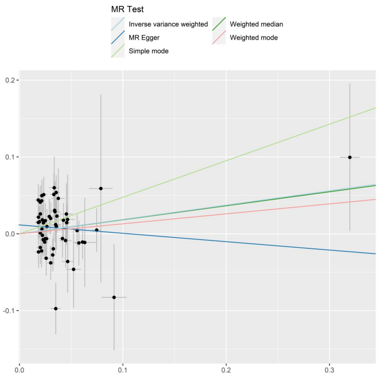
MR Test
Inverse variance weighted
MR Egger
Simple mode
Weighted median
Weighted mode

(V)

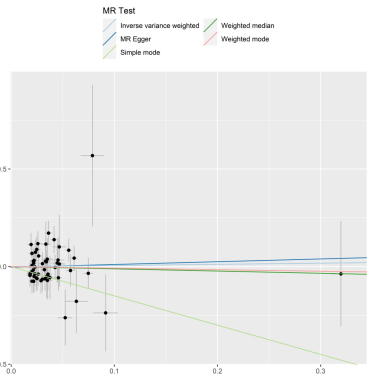
MR Test
Inverse variance weighted
MR Egger
Simple mode
Weighted median
Weighted mode

Figure S3:Scatter plot of the causal effect of bioavailable testosterone on different types of cancer, with the slope of each line corresponding to the estimated causal effect per method: (A) Head and neck cancer; (B) Brain cancer; (C) Thyroid cancer; (D) Oesophageal cancer; (E) Lip, oral,pharynx cancer; (F) Stomach cancer; (G) Liver and intrahepatic bile ducts cancer; (H) Pancreatic cancer; (I) Small intestine cancer; (J) Colon cancer; (K) Colorectal cancer; (L) Rectum cancer; (M) Non-small cell lung cancer; (N) Adenocarcinoma cell lung cancer; (O) Squamous cell lung cancer; (P) Bladder cancer; (Q) Prostate cancer; (R) Kidney cancer; (S) Testis cancer; (T) Malignant melanoma; (U) Malignant neoplasm of male genital organs; (V) Multiple myeloma and malignant plasma cell neoplasms.

(A)

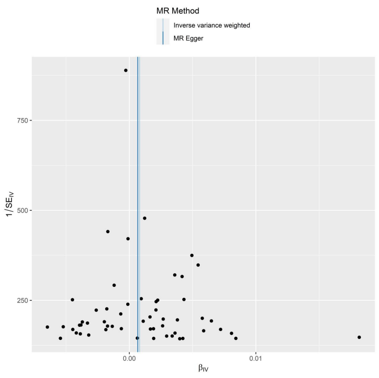

(B)

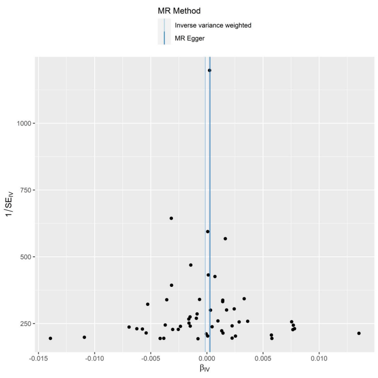

(C)

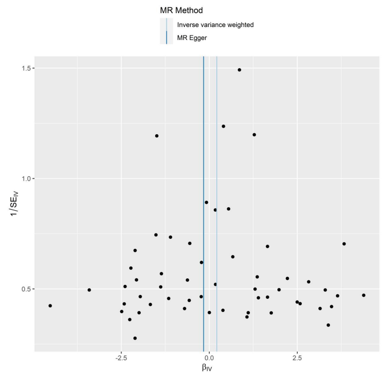

(D)

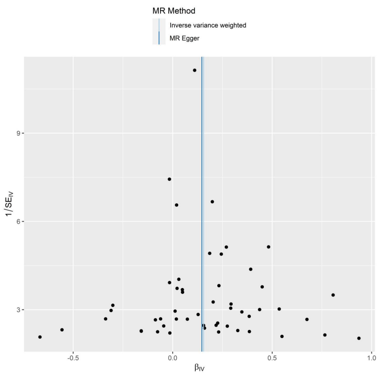

(E)

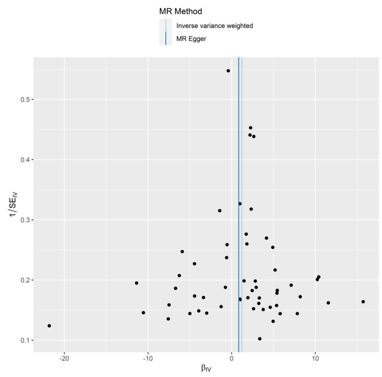

(F)

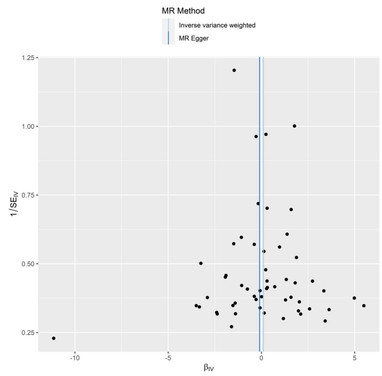

(G)

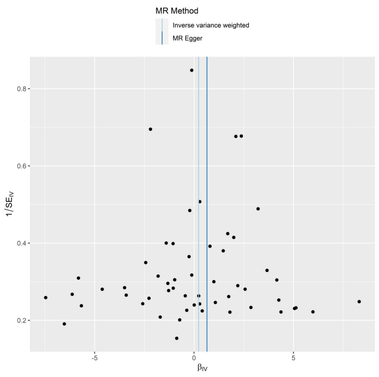

(H)

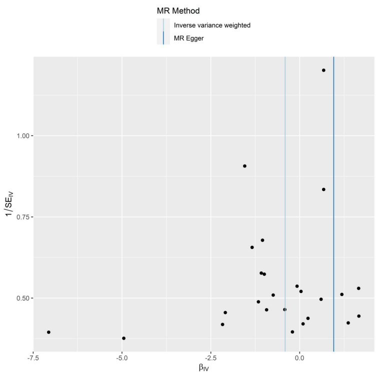

(I)

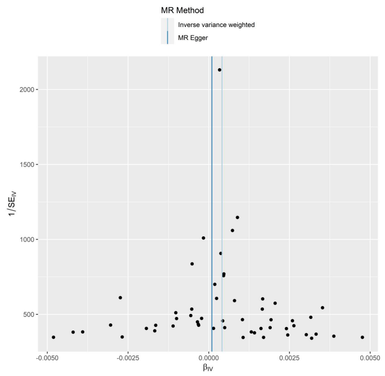

(J)

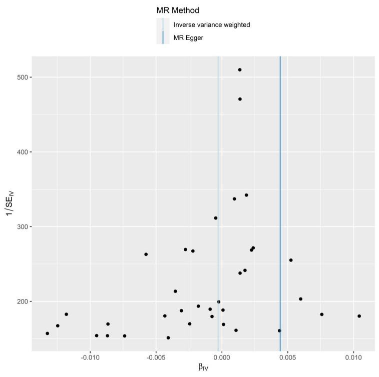

(K)

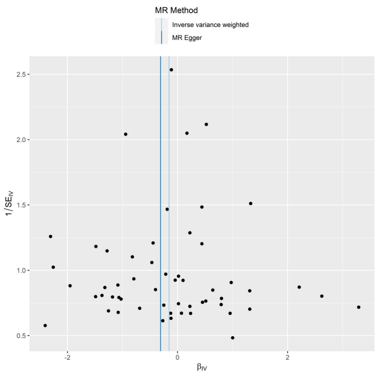

(L)

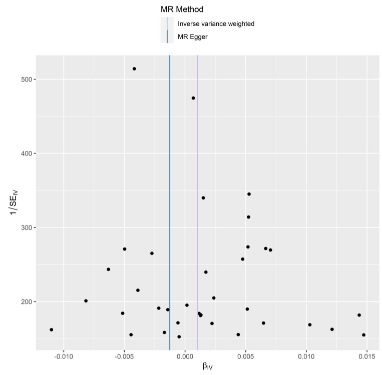

(M)

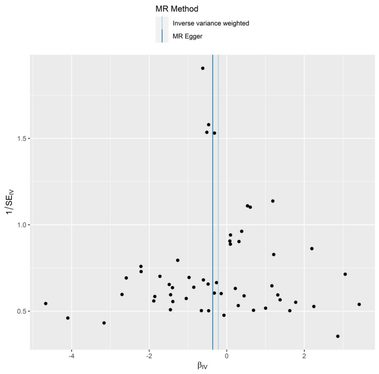
MR Method
Inverse variance weighted
MR Egger
1/SE_IV
β_IV

(N)

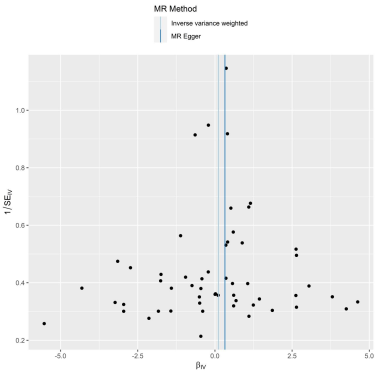
MR Method
Inverse variance weighted
MR Egger
1/SE_IV
β_IV

(O)

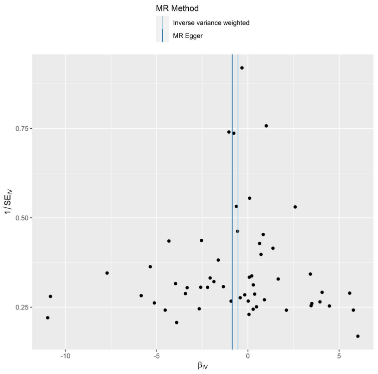
MR Method
Inverse variance weighted
MR Egger
1/SE_IV
β_IV

(P)

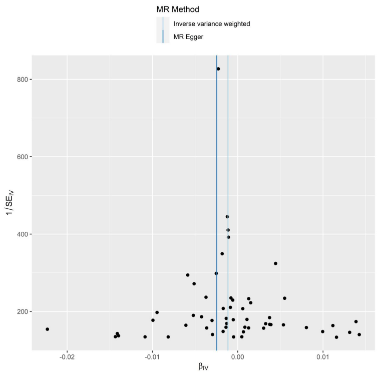
MR Method
Inverse variance weighted
MR Egger
1/SE_IV
β_IV

(Q)

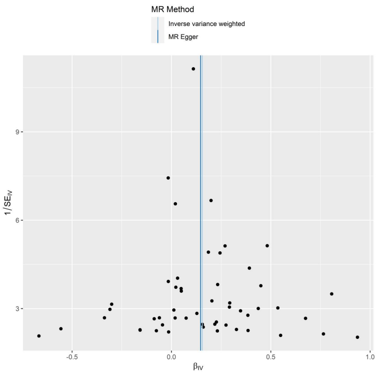
MR Method
Inverse variance weighted
MR Egger
1/SE_IV
β_IV

(R)

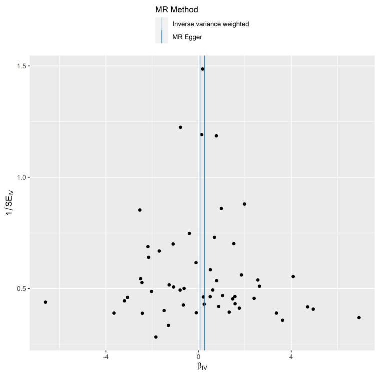
MR Method
Inverse variance weighted
MR Egger
1/SE_IV
β_IV

(S)

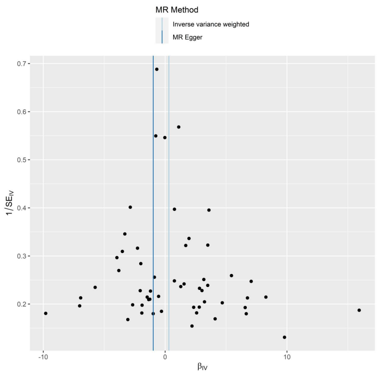
MR Method
Inverse variance weighted
MR Egger
1/SE_IV
β_IV

(T)

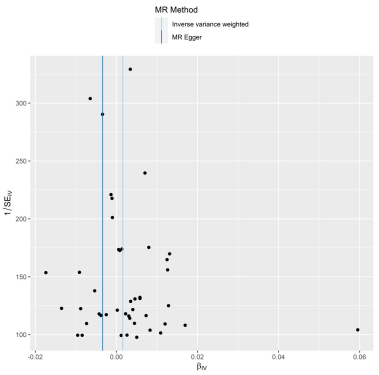
MR Method
Inverse variance weighted
MR Egger
1/SE_IV
β_IV

(U)

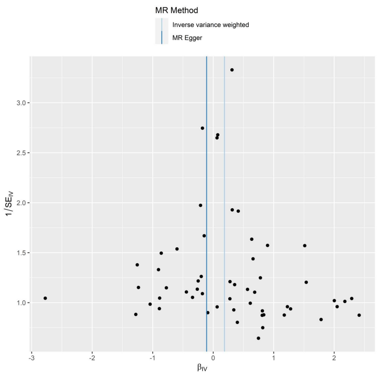
MR Method
Inverse variance weighted
MR Egger
1/SE_IV
β_IV

(V)

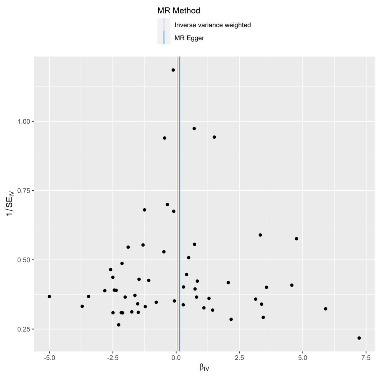
MR Method
Inverse variance weighted
MR Egger
1/SE_IV
β_IV

Figure S4:Funnel plot of the causal effect of bioavailable testosterone on different types of cancer: (A) Head and neck cancer; (B) Brain cancer; (C) Thyroid cancer; (D) Colon cancer; (E) Colorectal cancer; (F) Rectum cancer; (G) Lip, oral, pharynx cancer; (H) Liver and intrahepatic bile ducts cancer; (I) Non-small cell lung cancer; (J) Adenocarcinoma cell lung cancer; (K) Squamous cell lung cancer; (L) Stomach cancer; (M) Small intestine cancer; (N)Bladder cancer; (O) Malignant melanoma; (P) Malignant neoplasm of male genital organs ; (Q)Multiple myeloma and malignant plasma cell neoplasms; (R) Oesophageal cancer; (S) Pancreatic cancer; (T) Prostate cancer; (U) Kidney cancer; (V) Testis cancer.

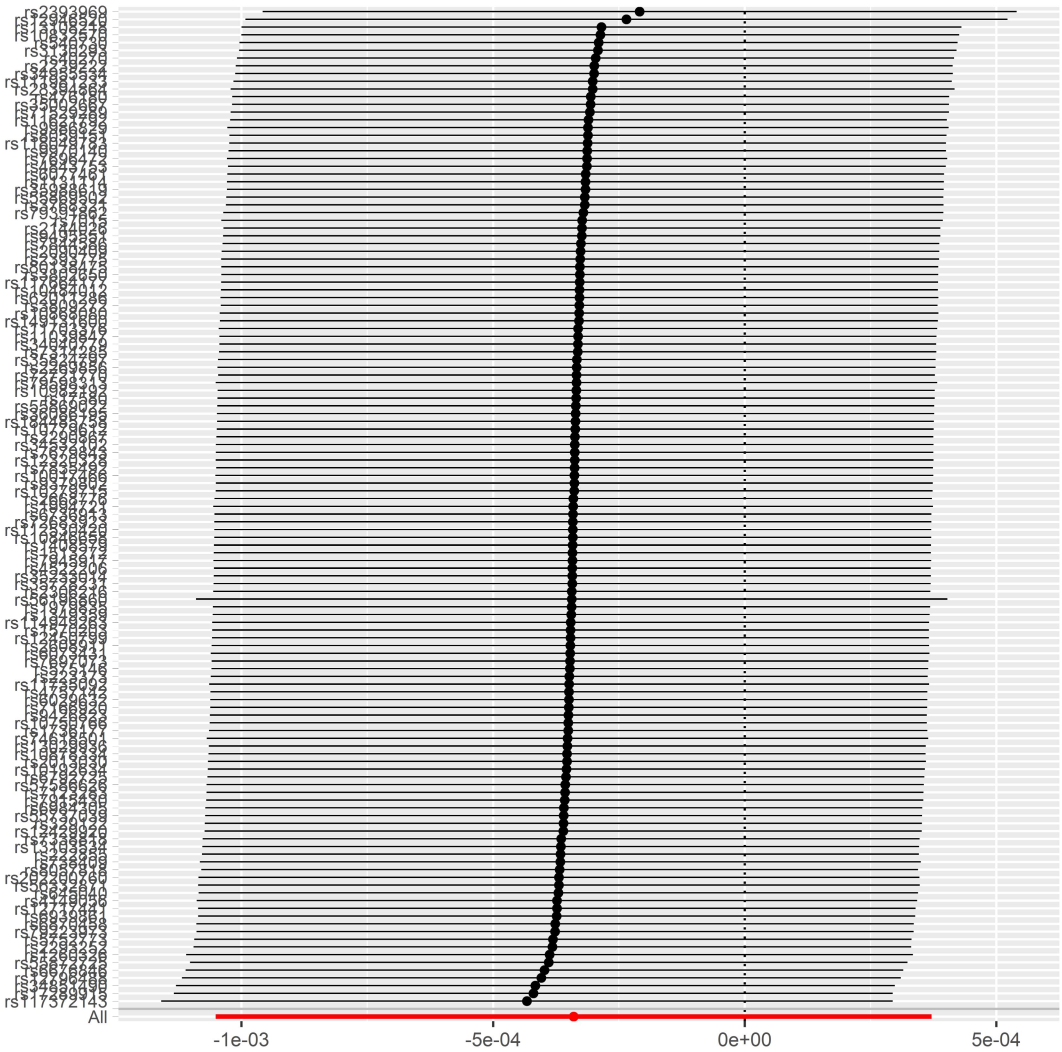

Figure S5:Leave-one-out inverse-variance weighted mendelian randomization analyses of total testosterone on Head and neck cancer

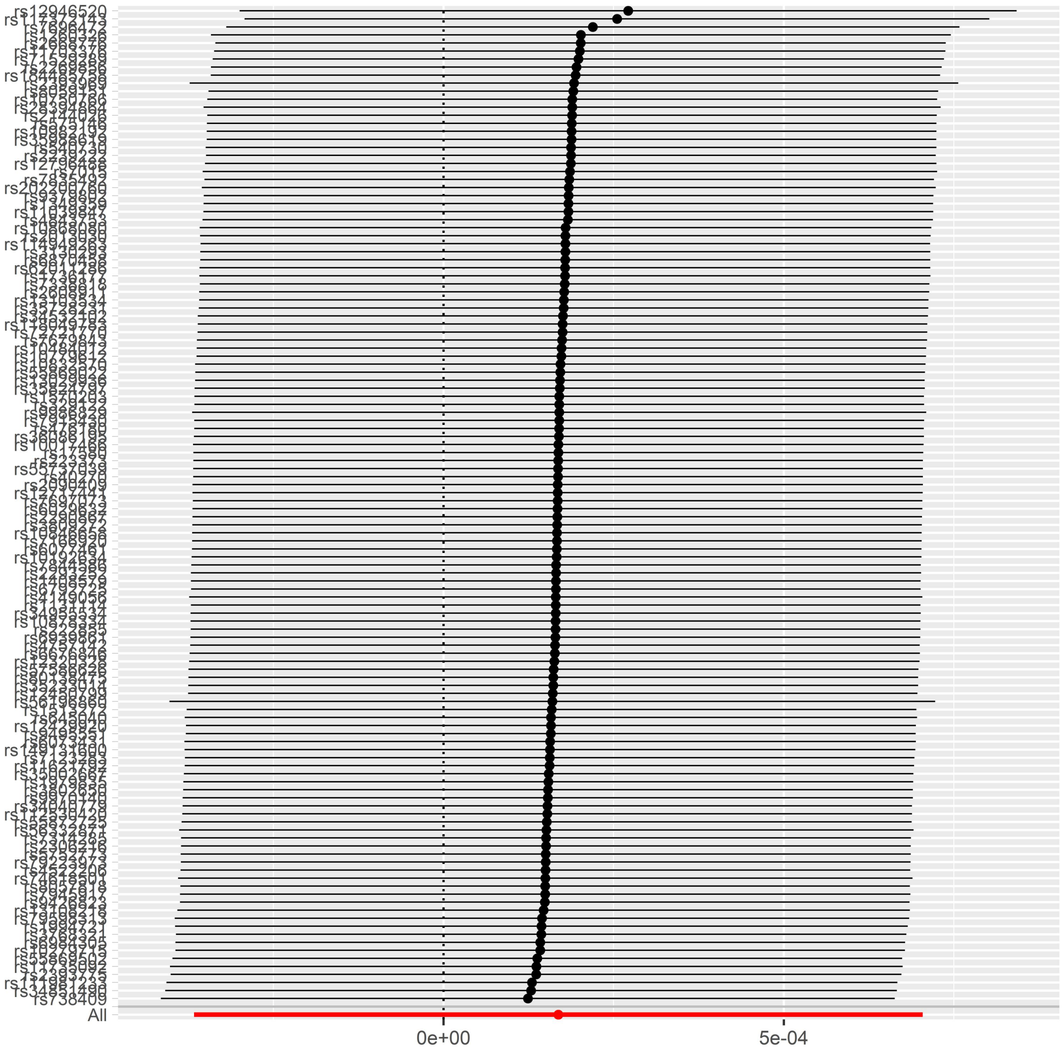

Figure S6:Leave-one-out inverse-variance weighted mendelian randomization analyses of total testosterone on Brain cancer

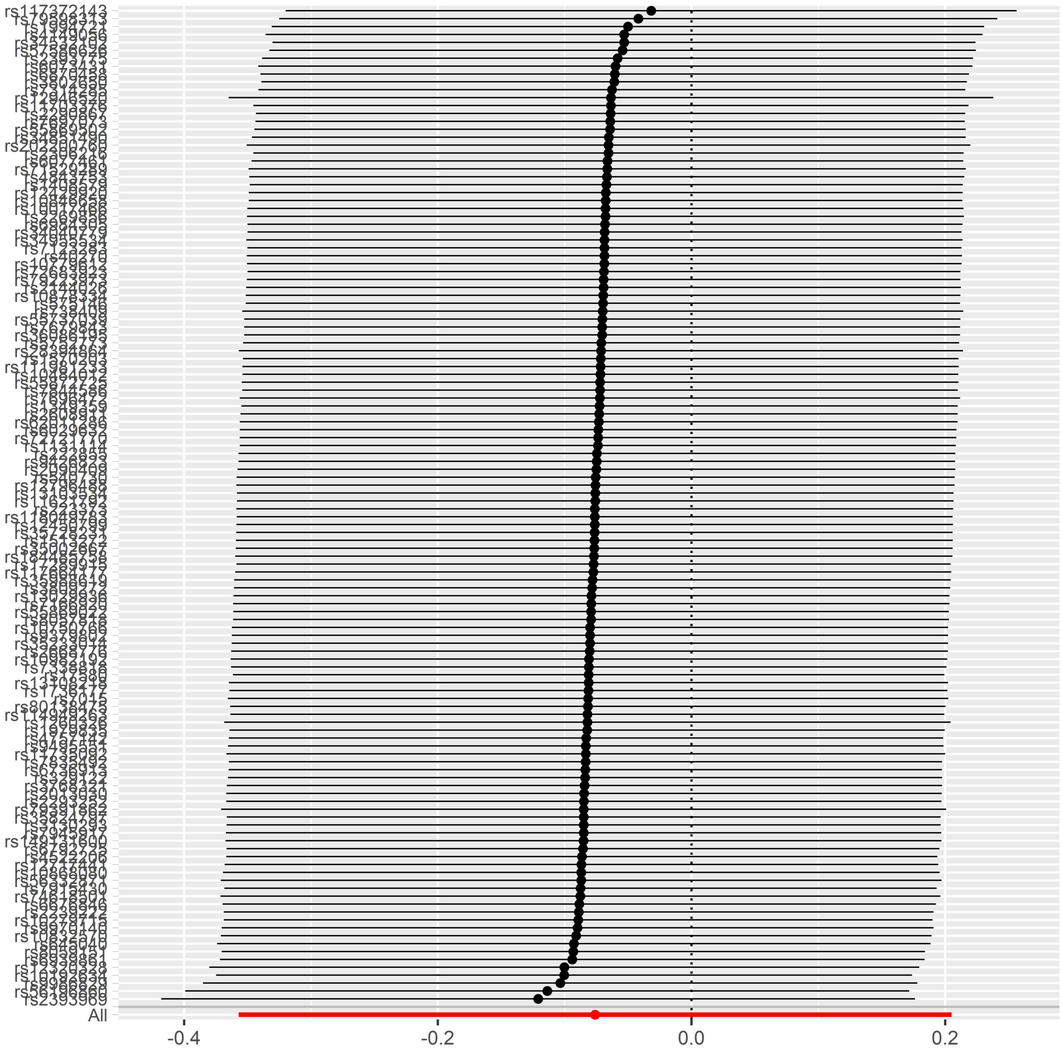

Figure S8:Leave-one-out inverse-variance weighted mendelian randomization analyses of total testosterone on Thyroid cancer

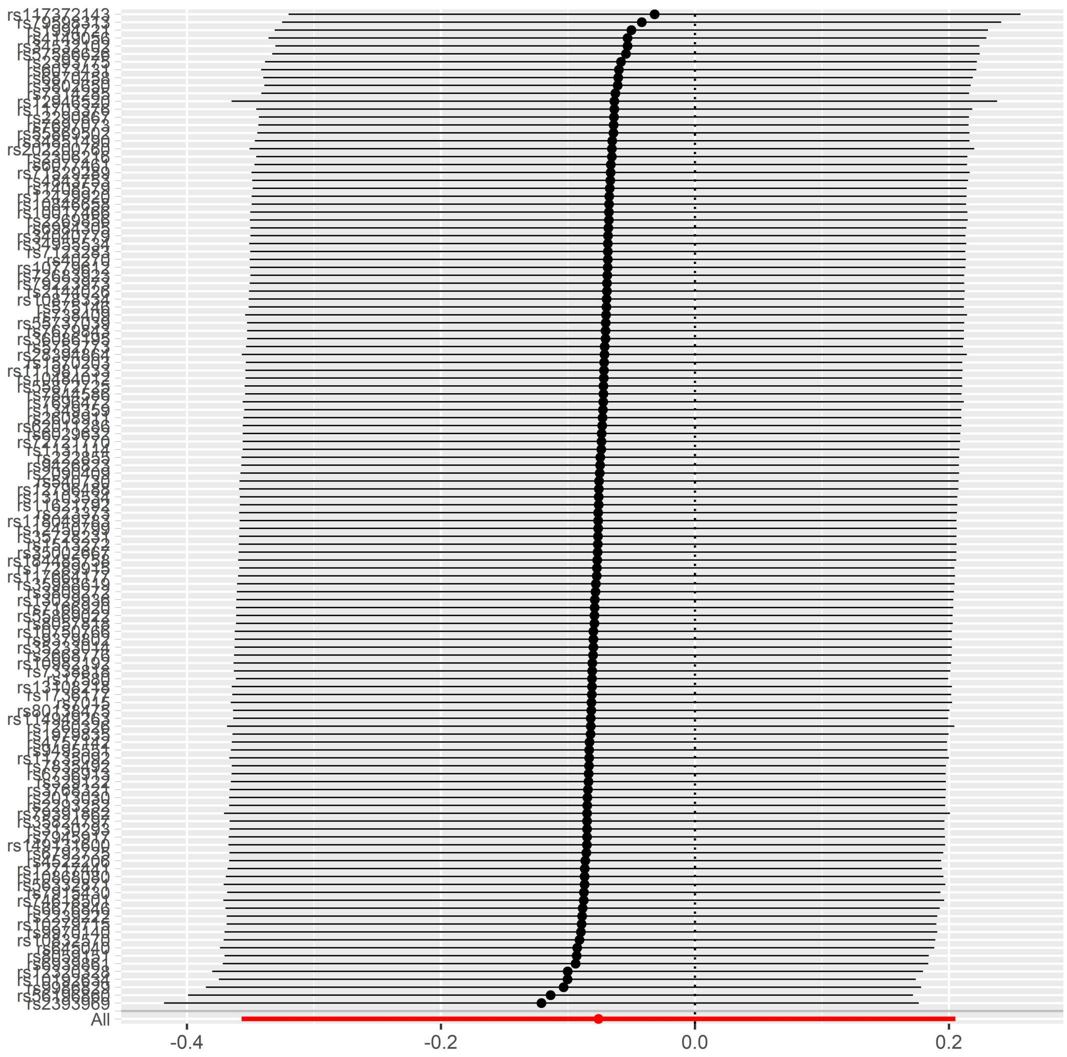

Figure S9:Leave-one-out inverse-variance weighted mendelian randomization analyses of total testosterone on Oesophageal cancer

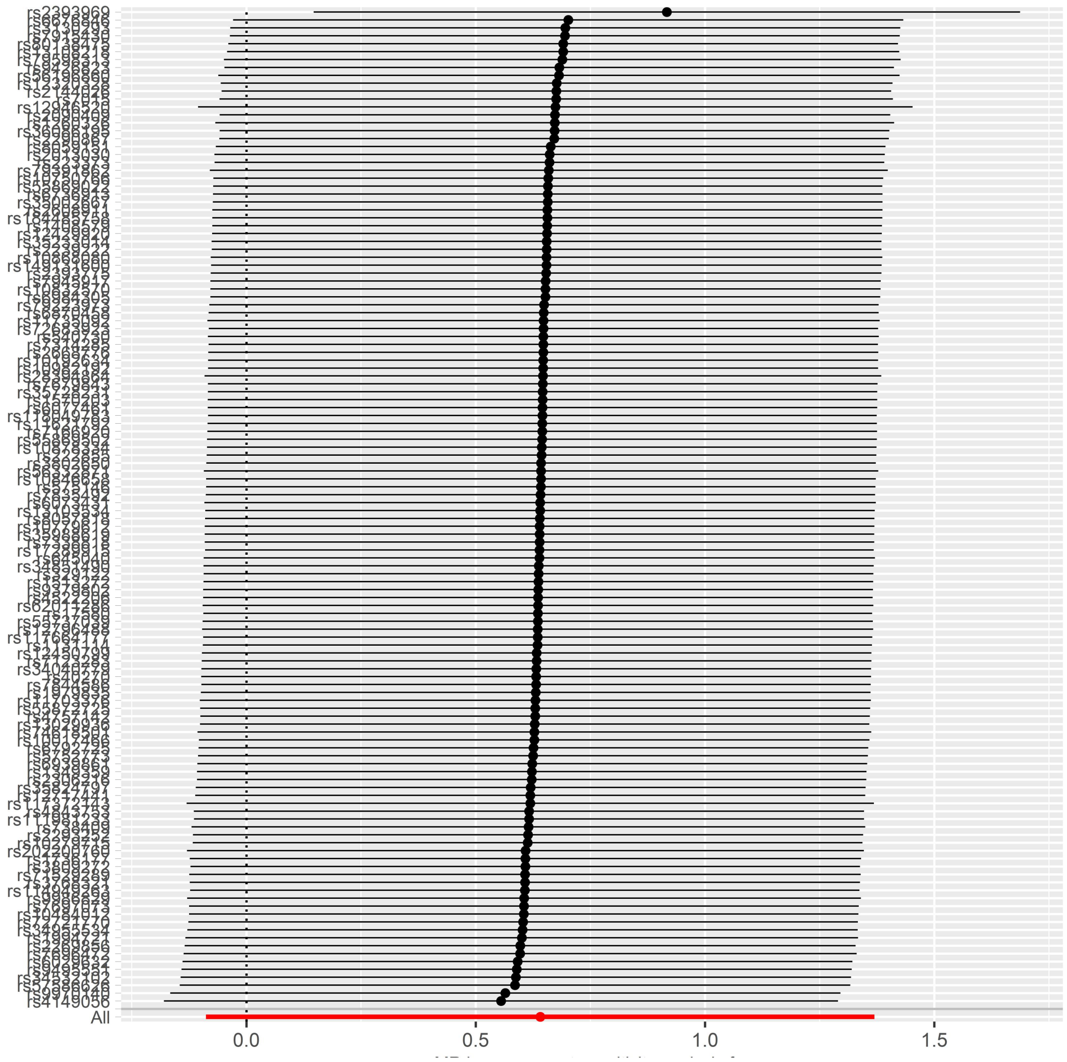

Figure S10:Leave-one-out inverse-variance weighted mendelian randomization analyses of total testosterone on Lip, oral, and, pharynx cancer

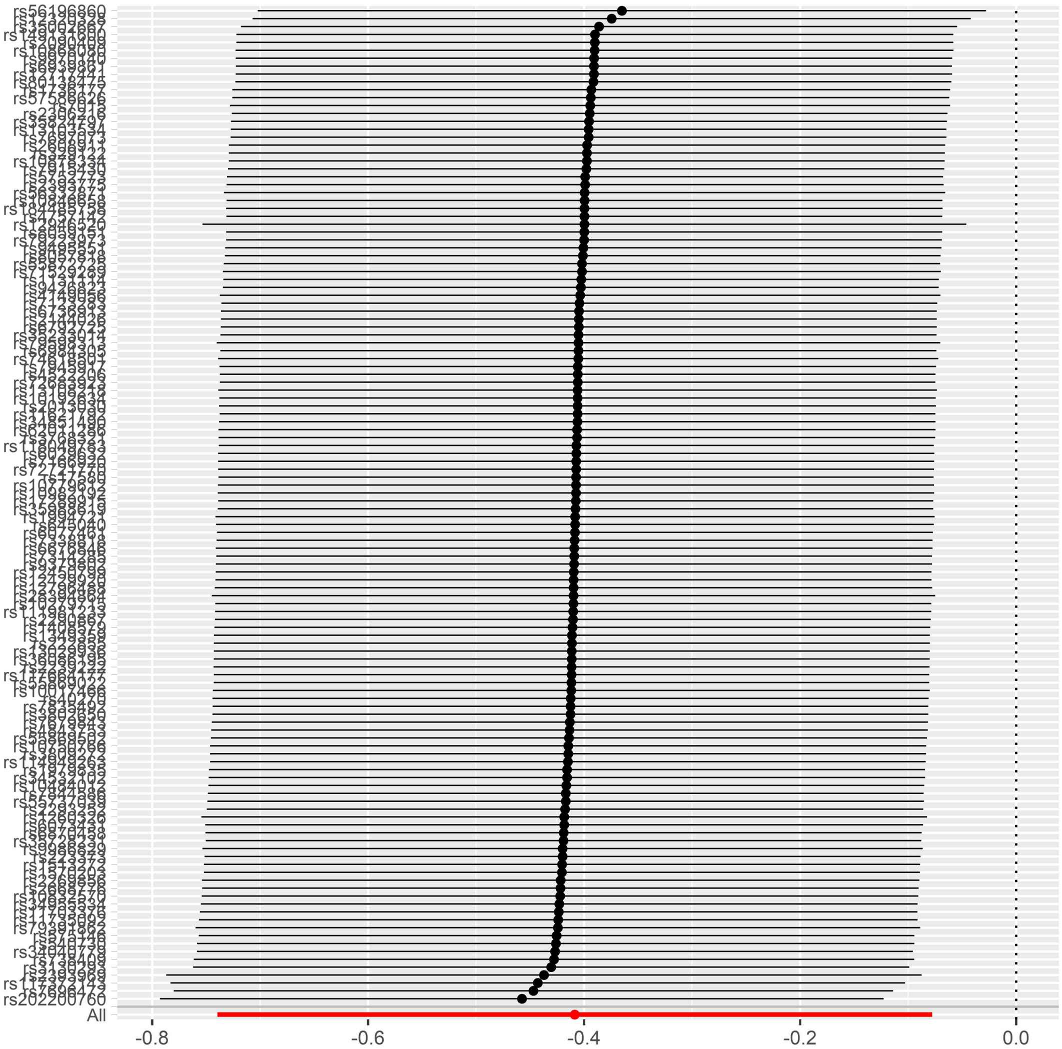

Figure S11:Leave-one-out inverse-variance weighted mendelian randomization analyses of total testosterone on Stomach cancer

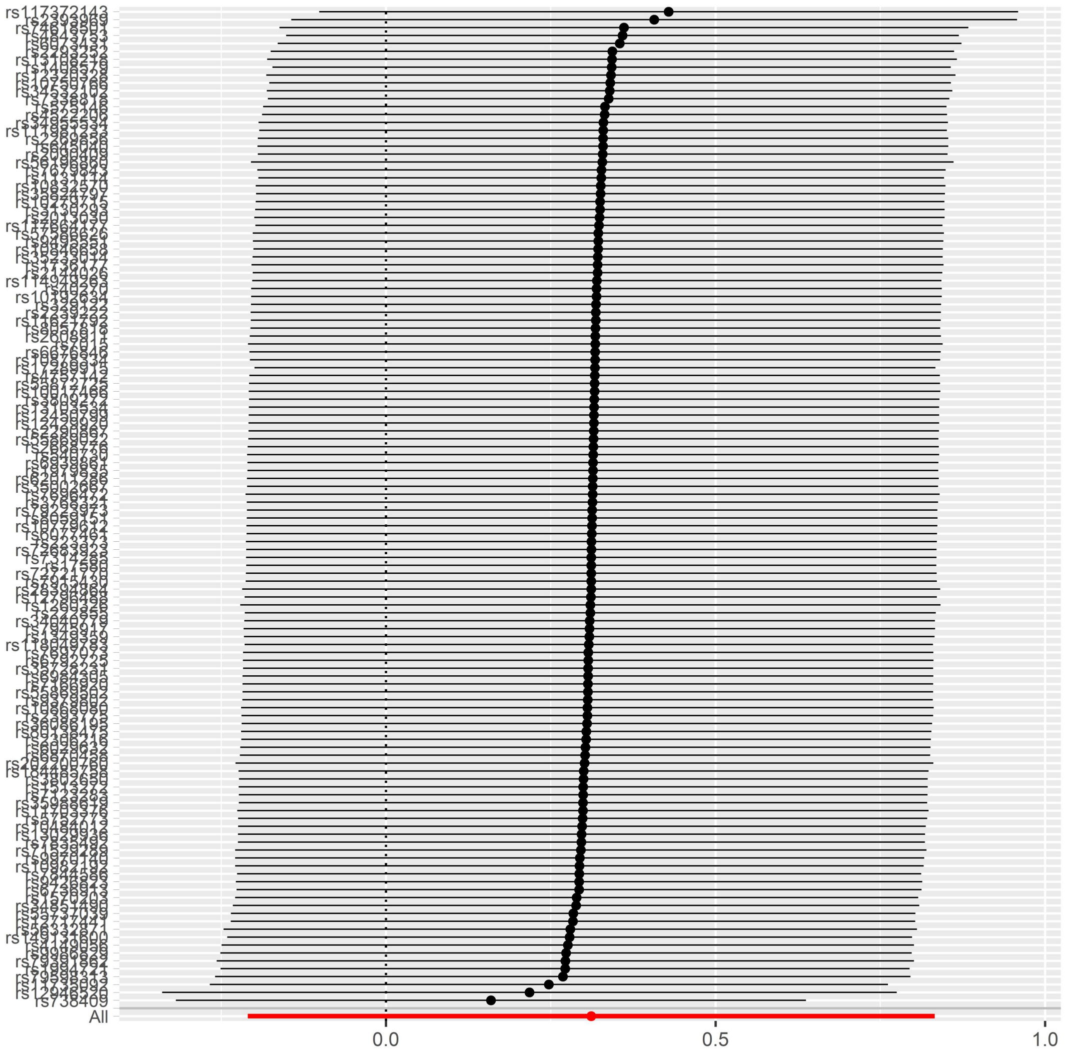

Figure S12:Leave-one-out inverse-variance weighted mendelian randomization analyses of total testosterone on Liver and intrahepatic bile ducts cancer

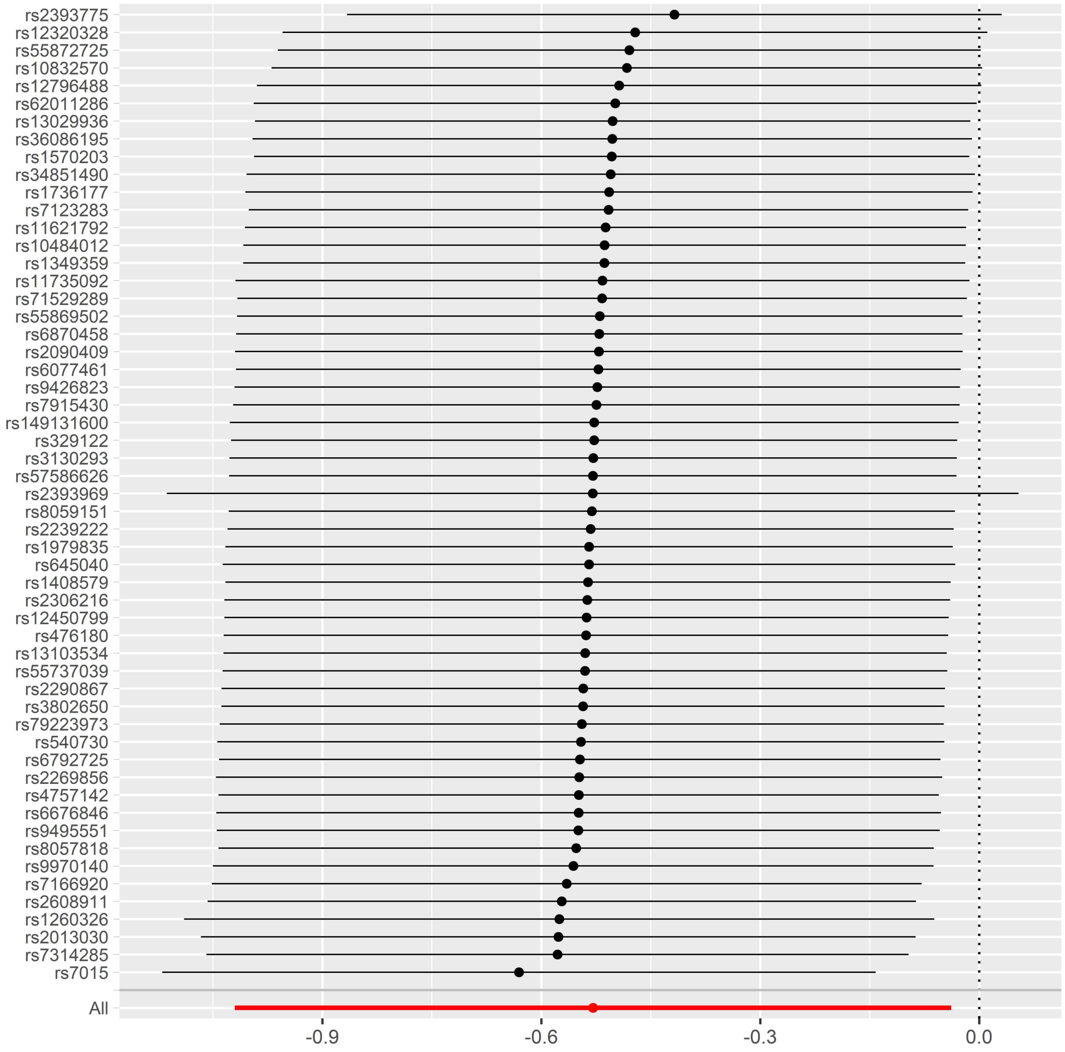

Figure S13:Leave-one-out inverse-variance weighted mendelian randomization analyses of total testosterone on Pancreatic cancer

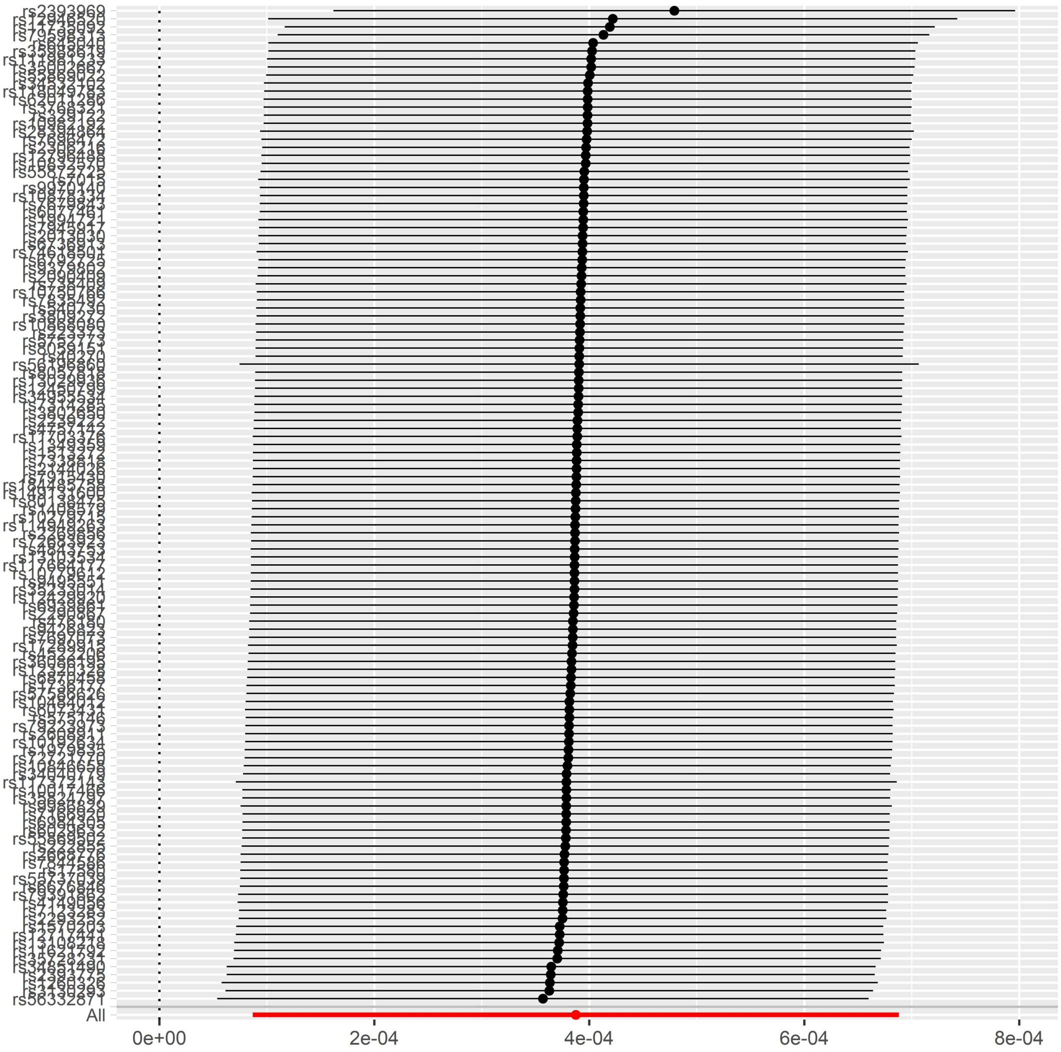

Figure S14:Leave-one-out inverse-variance weighted mendelian randomization analyses of total testosterone on Small intestine cancer

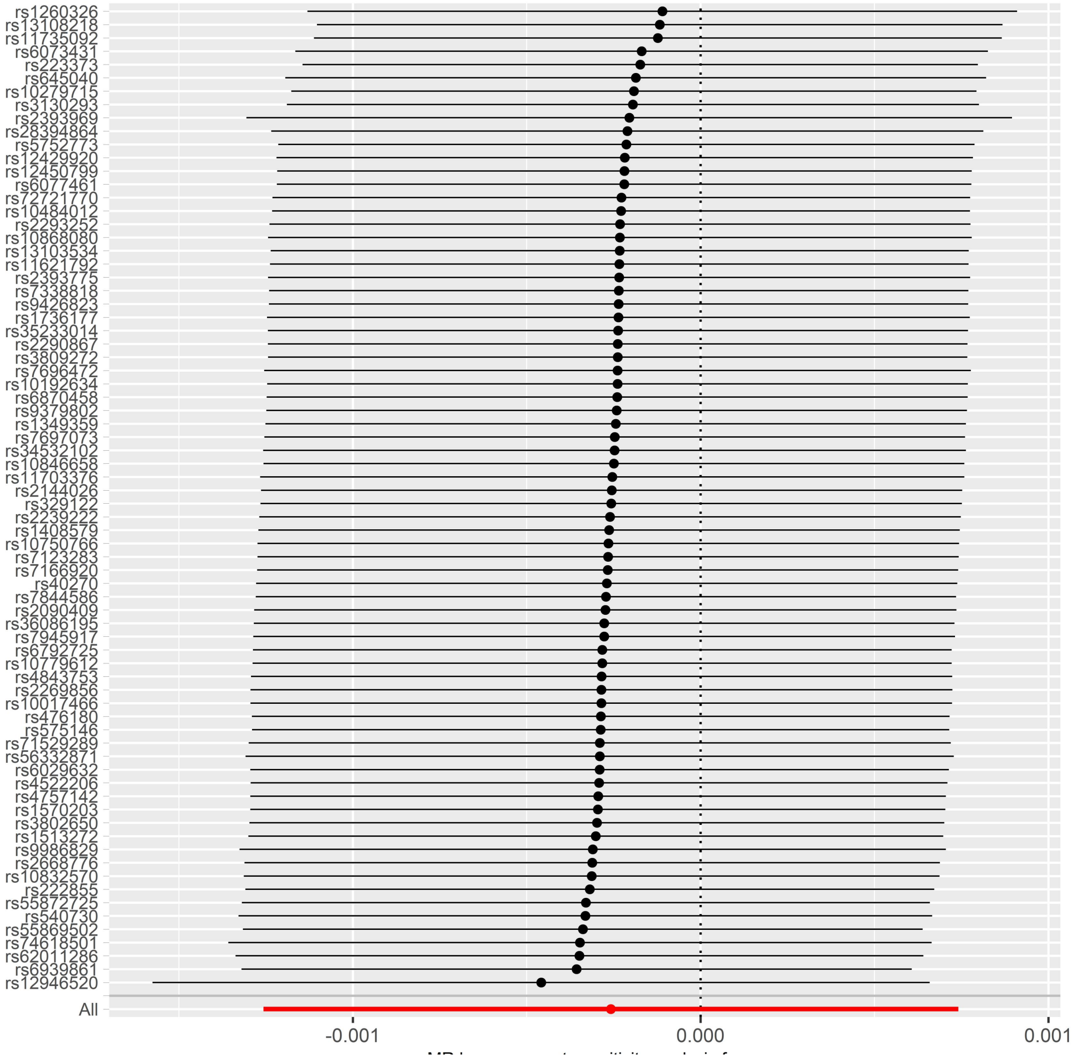

Figure S15:Leave-one-out inverse-variance weighted mendelian randomization analyses of total testosterone on Colon cancer

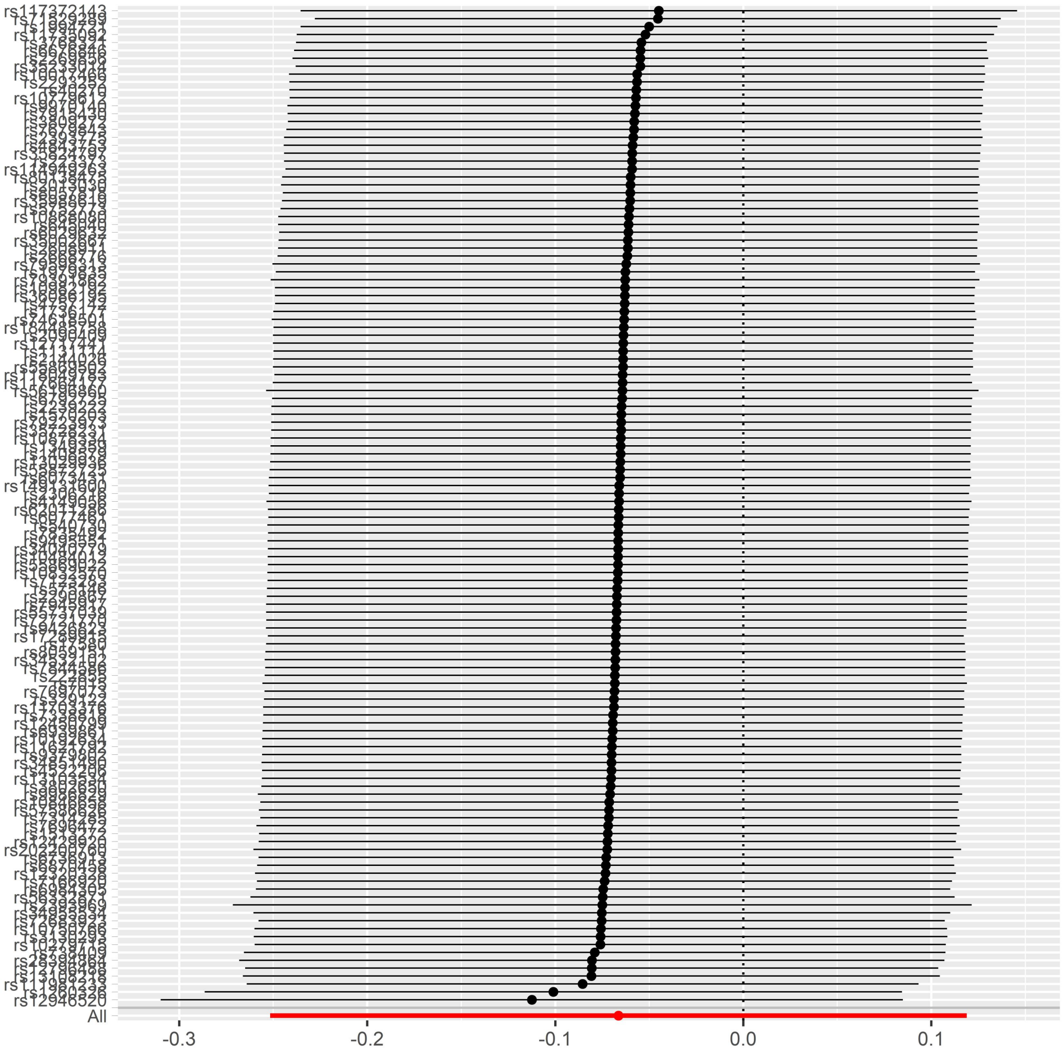

Figure S16:Leave-one-out inverse-variance weighted mendelian randomization analyses of total testosterone on Colorectal cancer

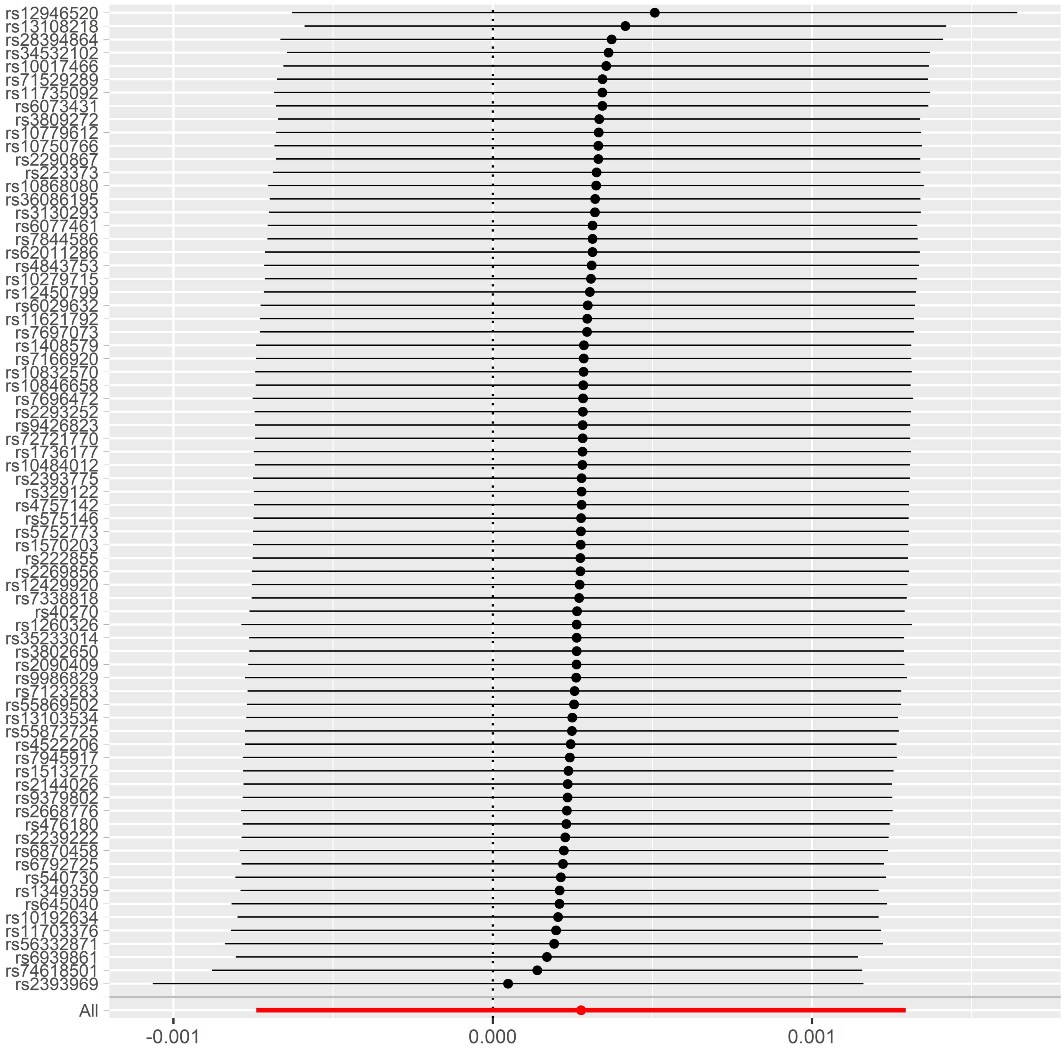

Figure S17:Leave-one-out inverse-variance weighted mendelian randomization analyses of total testosterone on Rectum cancer

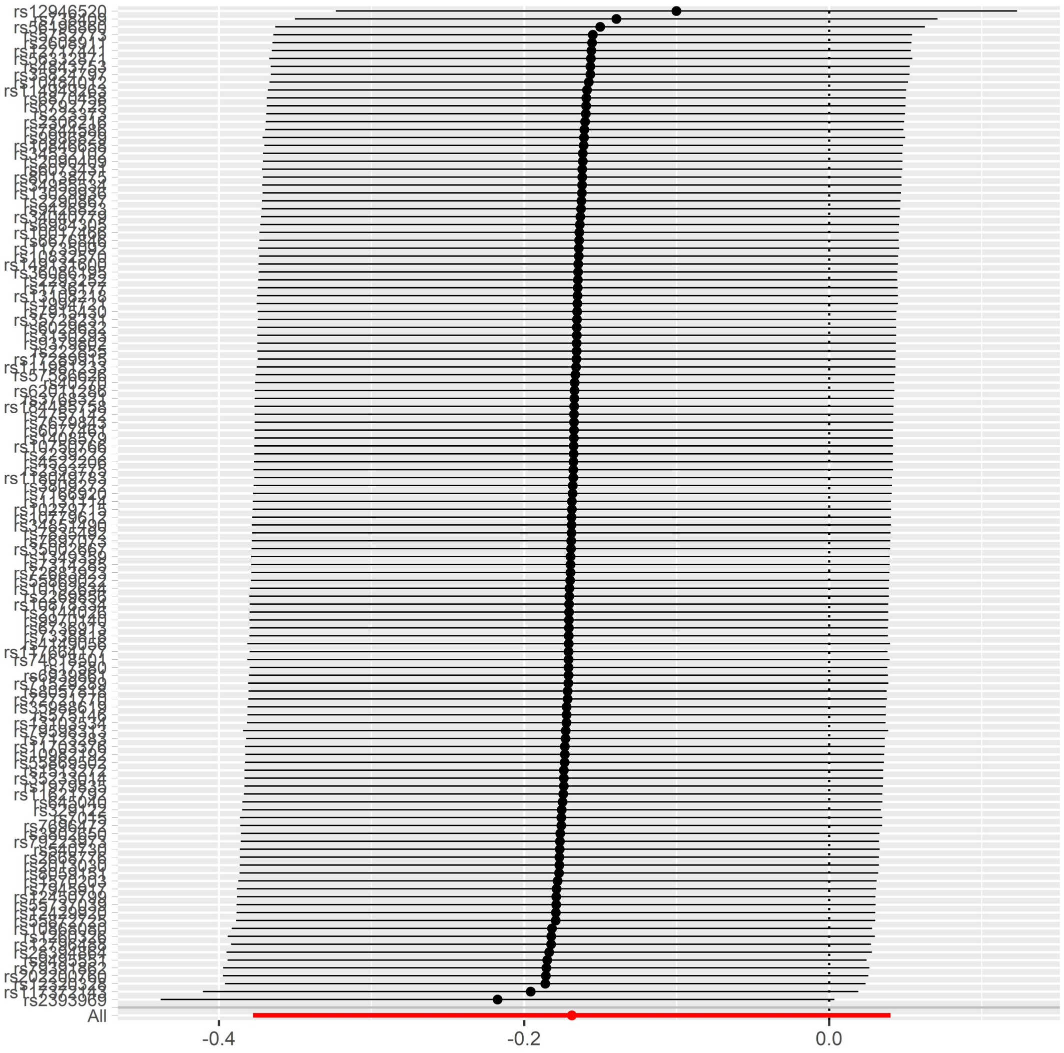

Figure S18:Leave-one-out inverse-variance weighted mendelian randomization analyses of total testosterone on Non-small cell lung cancer

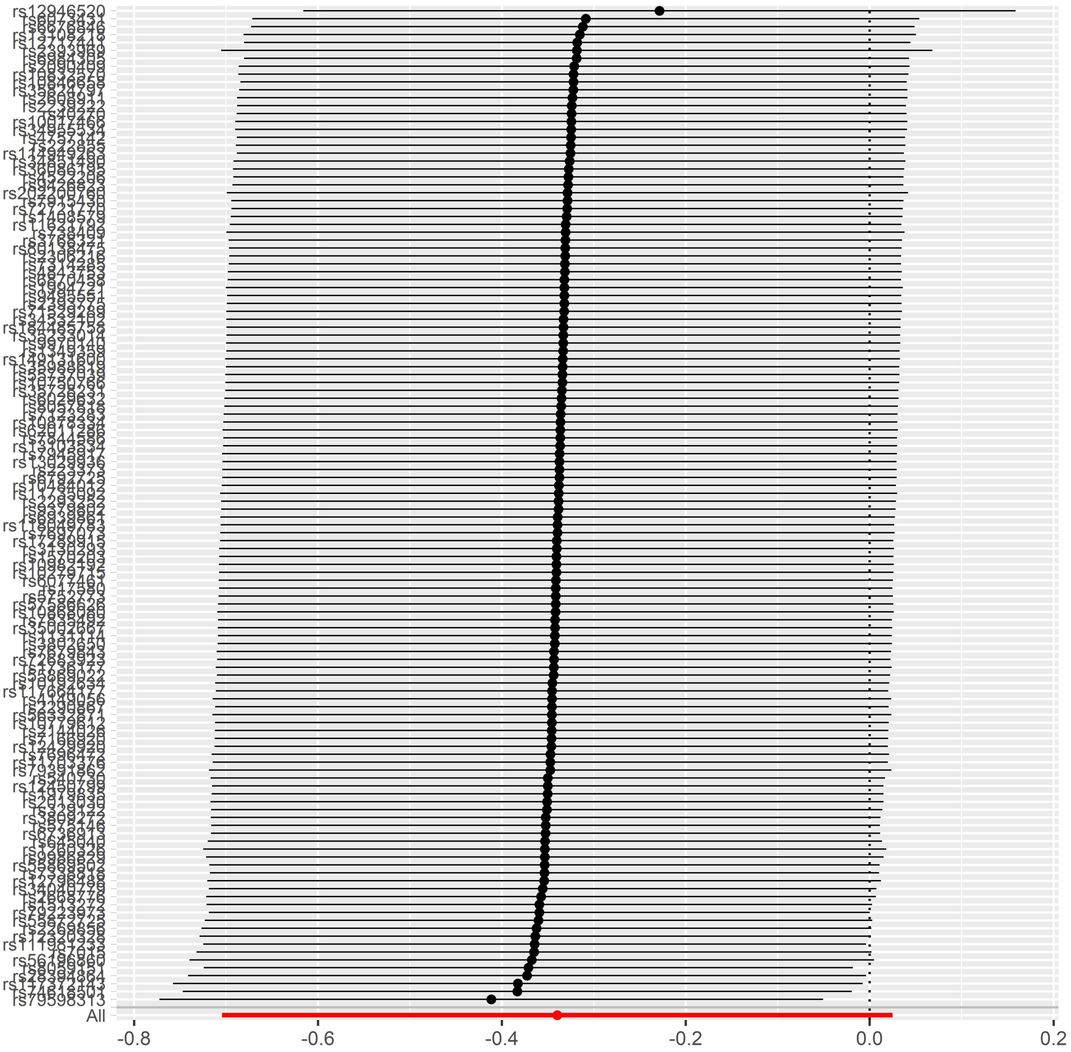

Figure S19:Leave-one-out inverse-variance weighted mendelian randomization analyses of total testosterone on Adenocarcinoma cell lung cancer

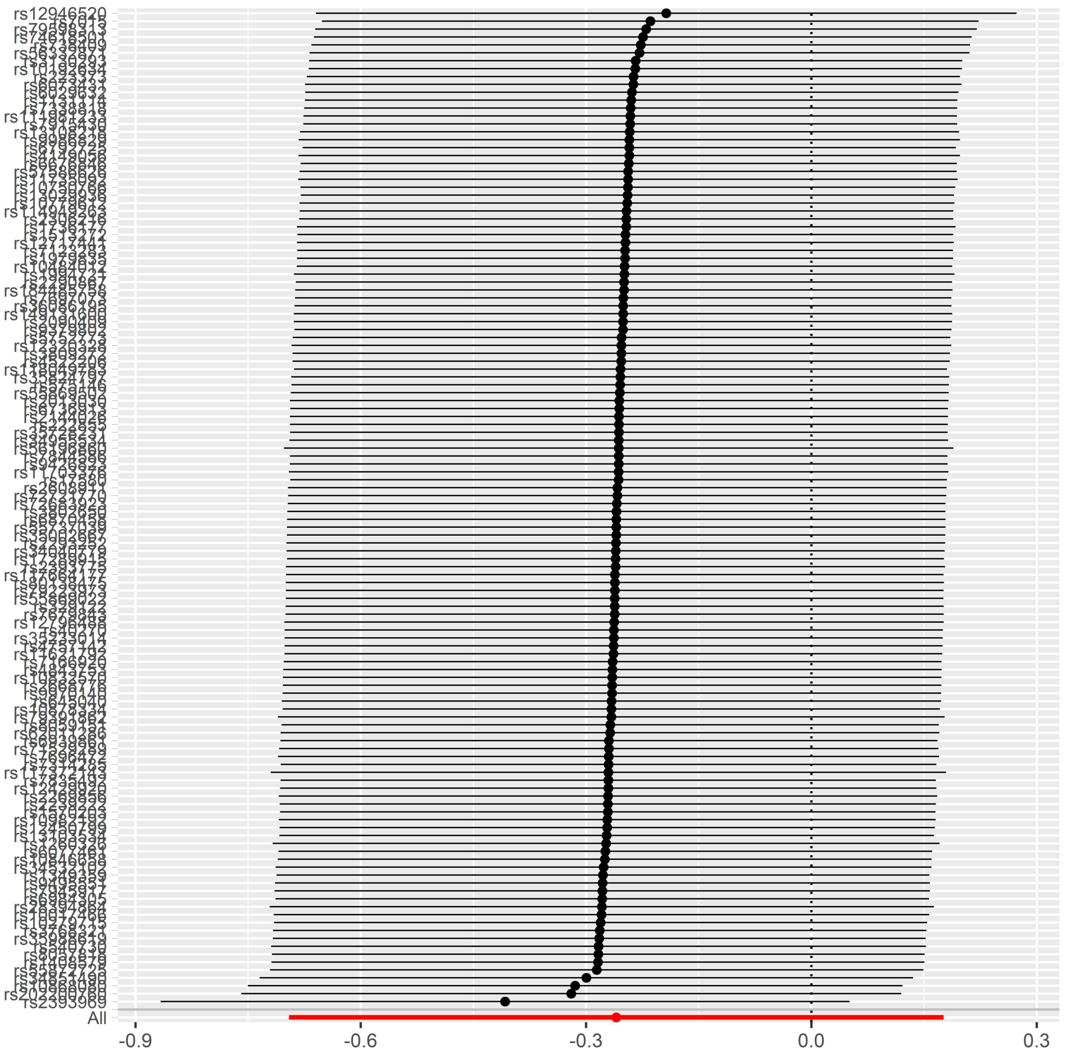

Figure S20:Leave-one-out inverse-variance weighted mendelian randomization analyses of total testosterone on Squamous cell lung cancer

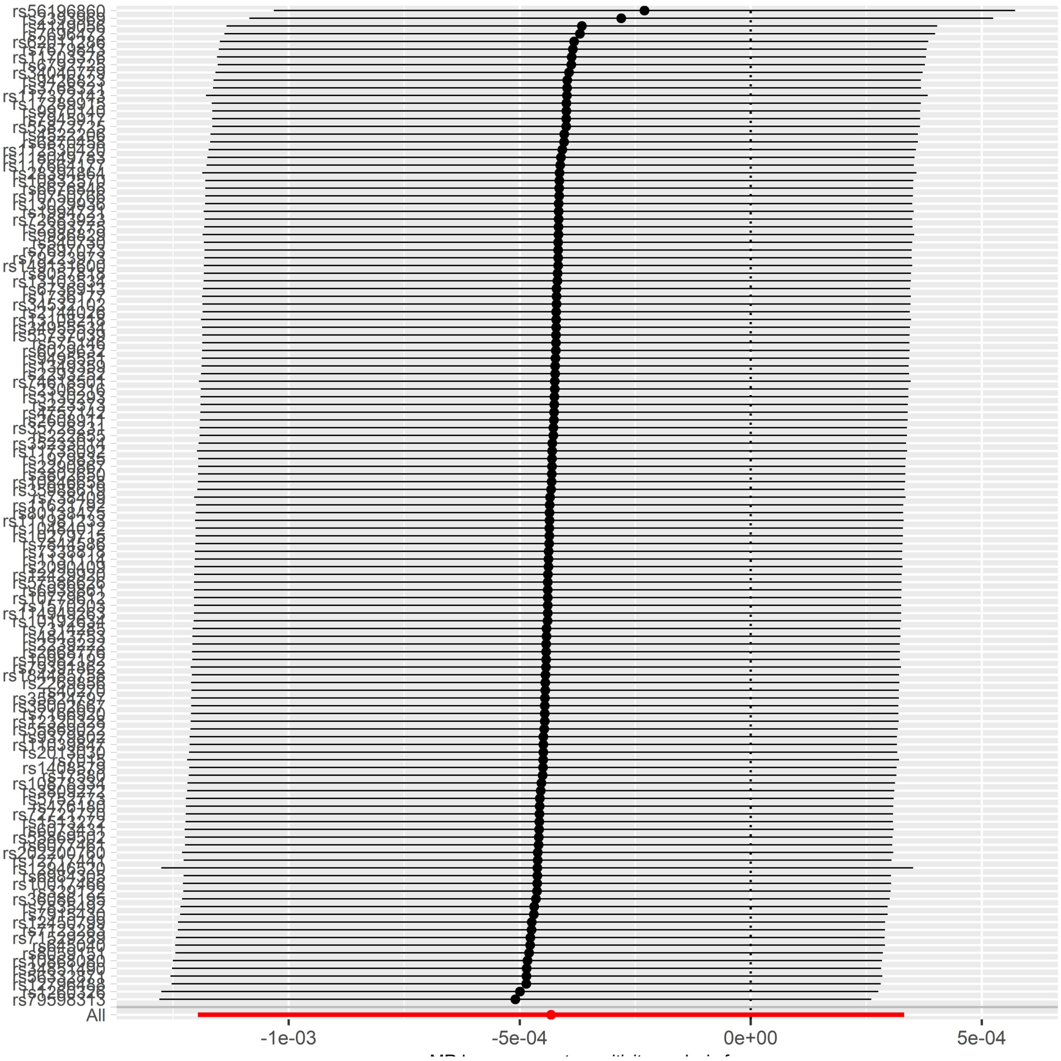

Figure S21:Leave-one-out inverse-variance weighted mendelian randomization analyses of total testosterone on Bladder cancer

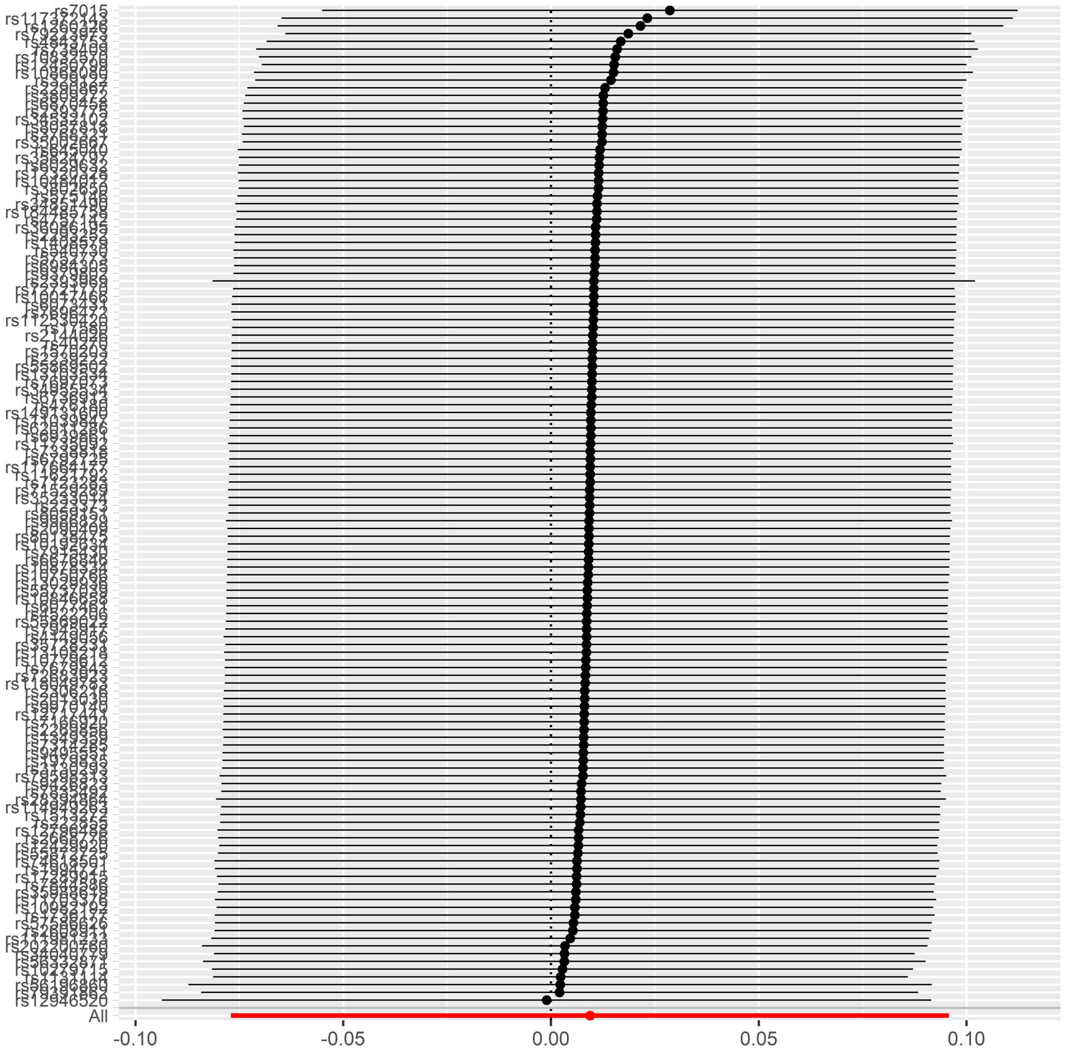

Figure S22:Leave-one-out inverse-variance weighted mendelian randomization analyses of total testosterone on Prostate cancer

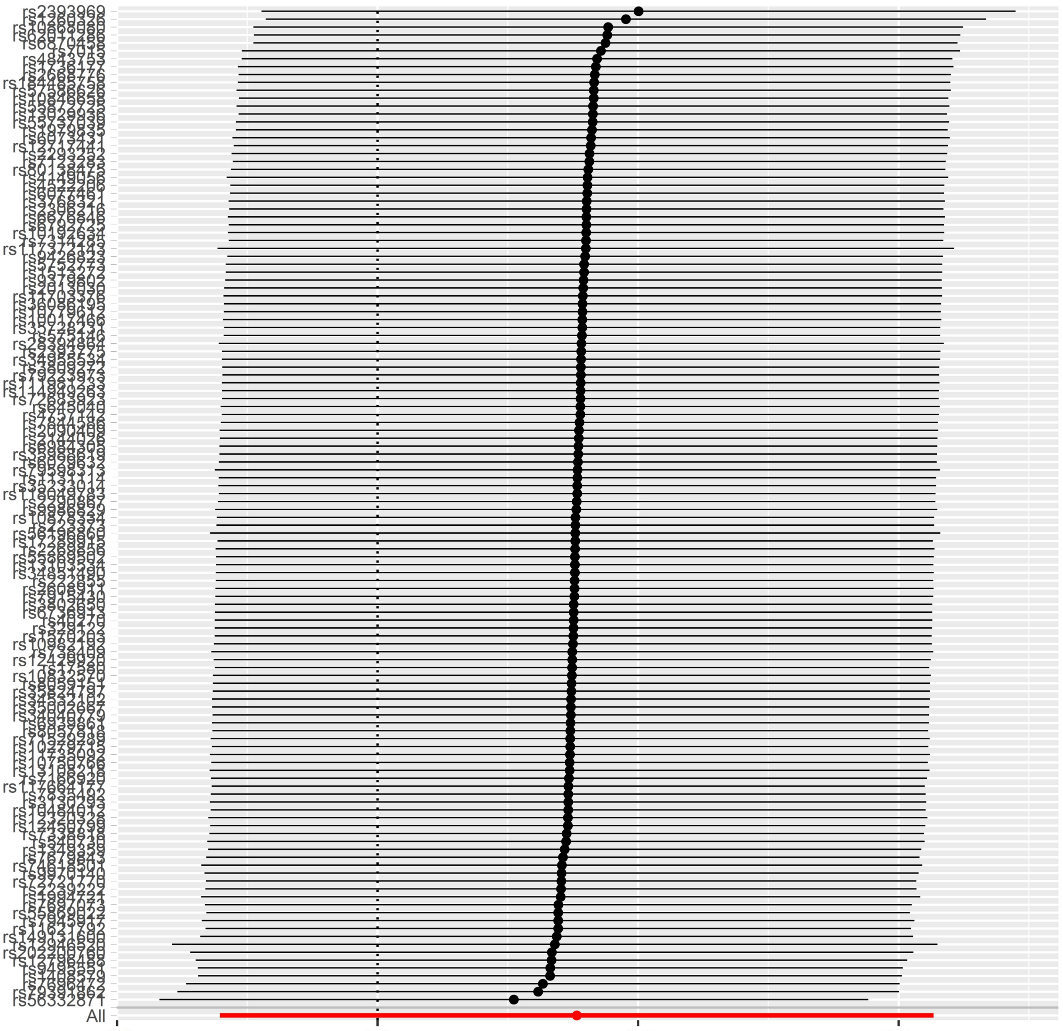

Figure S23:Leave-one-out inverse-variance weighted mendelian randomization analyses of total testosterone on Kidney cancer

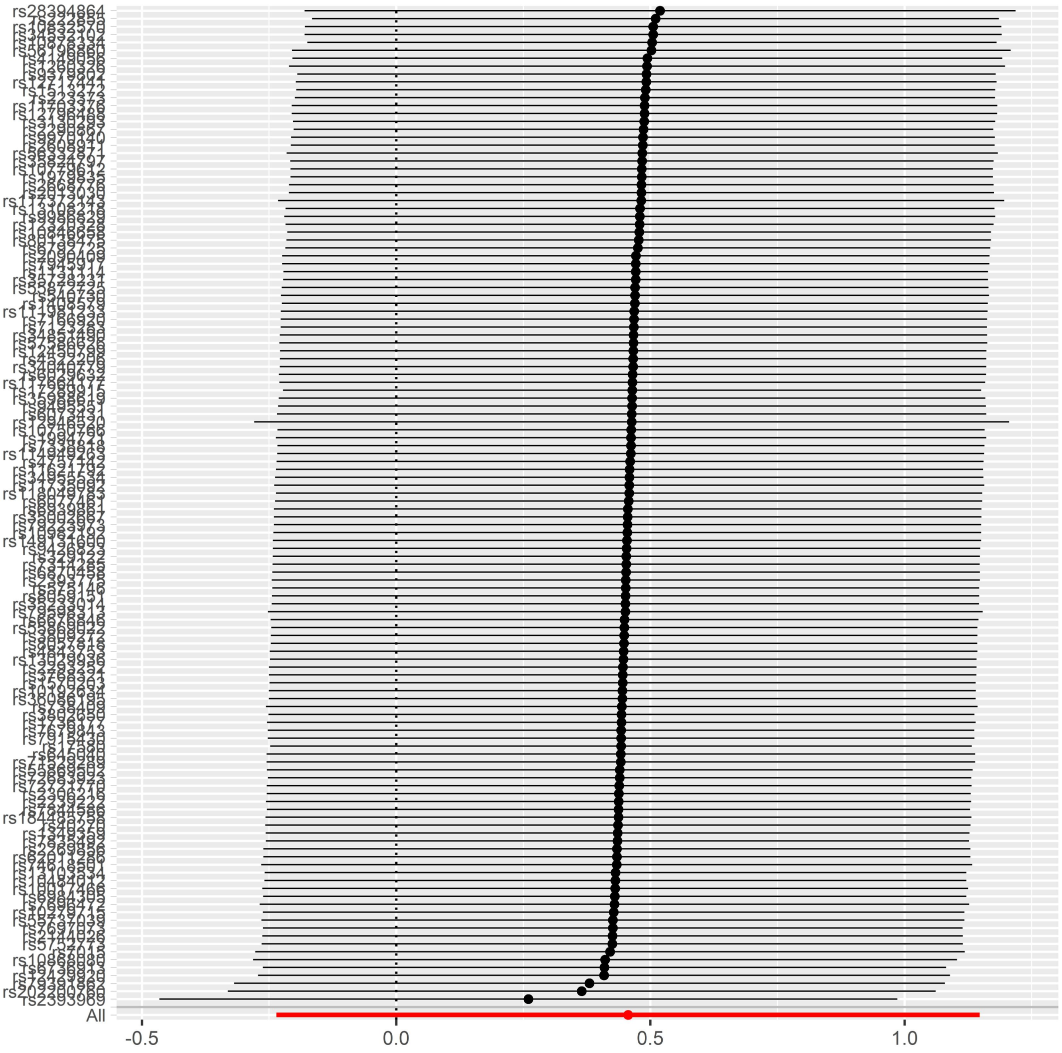

Figure S24:Leave-one-out inverse-variance weighted mendelian randomization analyses of total testosterone on Testis cancer

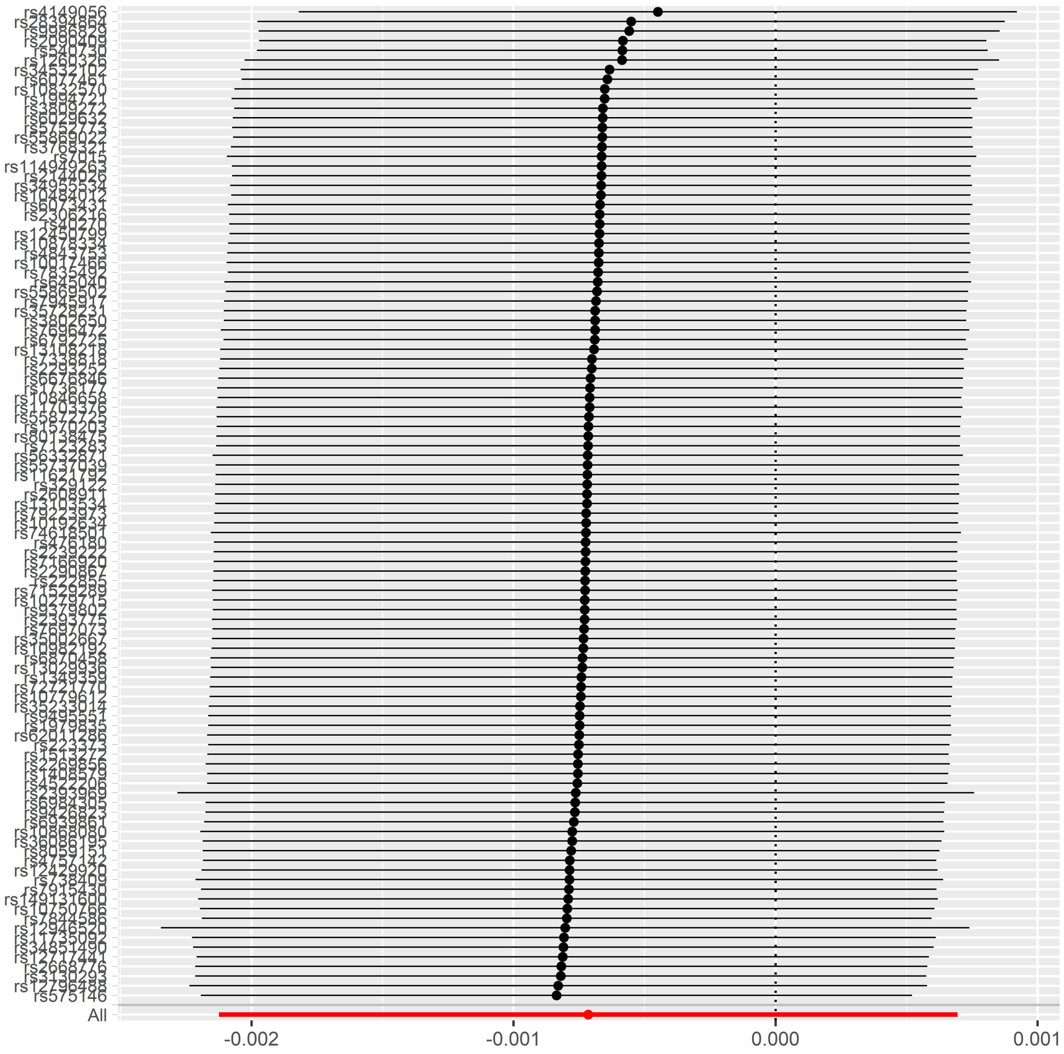

Figure S25:Leave-one-out inverse-variance weighted mendelian randomization analyses of total testosterone on Malignant melanoma

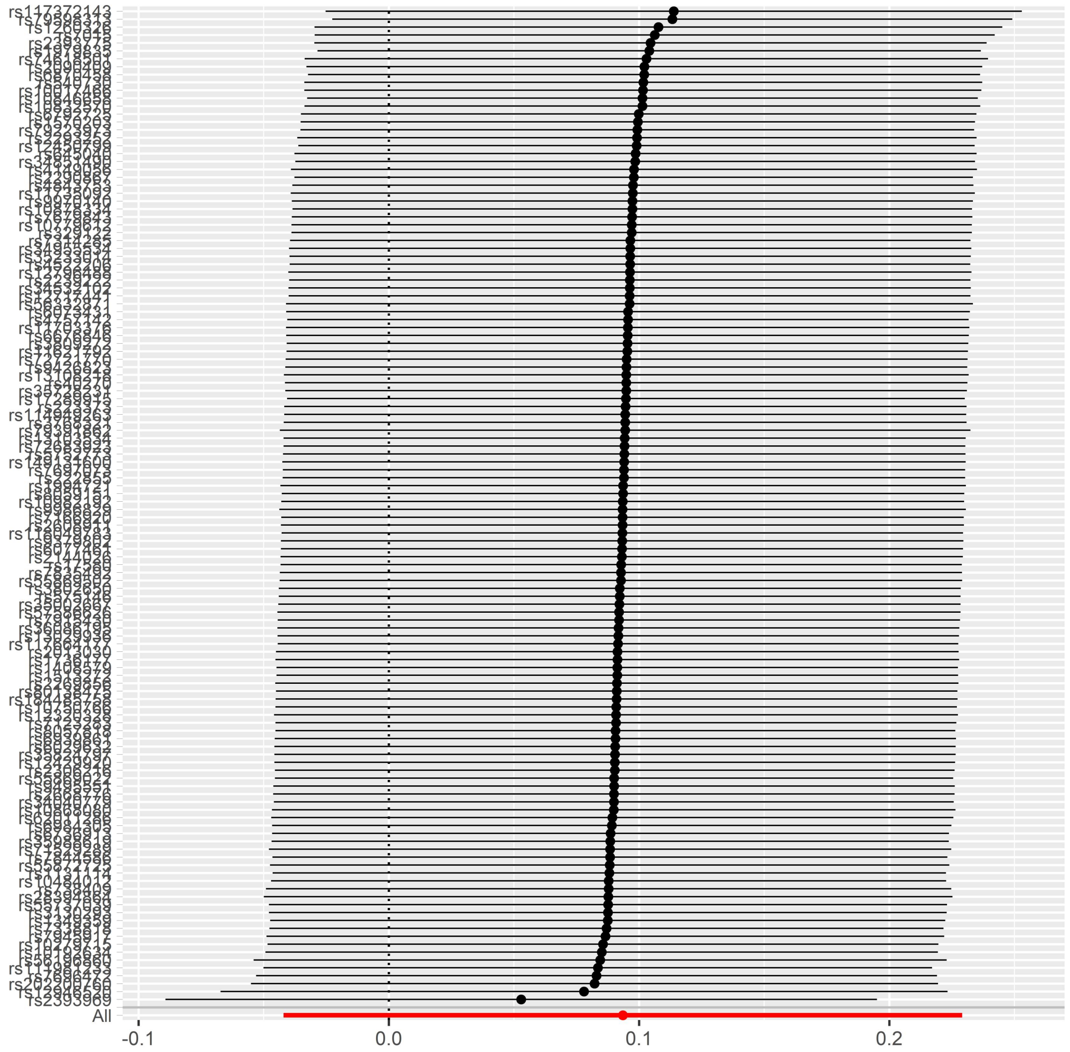

Figure S26:Leave-one-out inverse-variance weighted mendelian randomization analyses of total testosterone on Malignant neoplasm of male genital organs

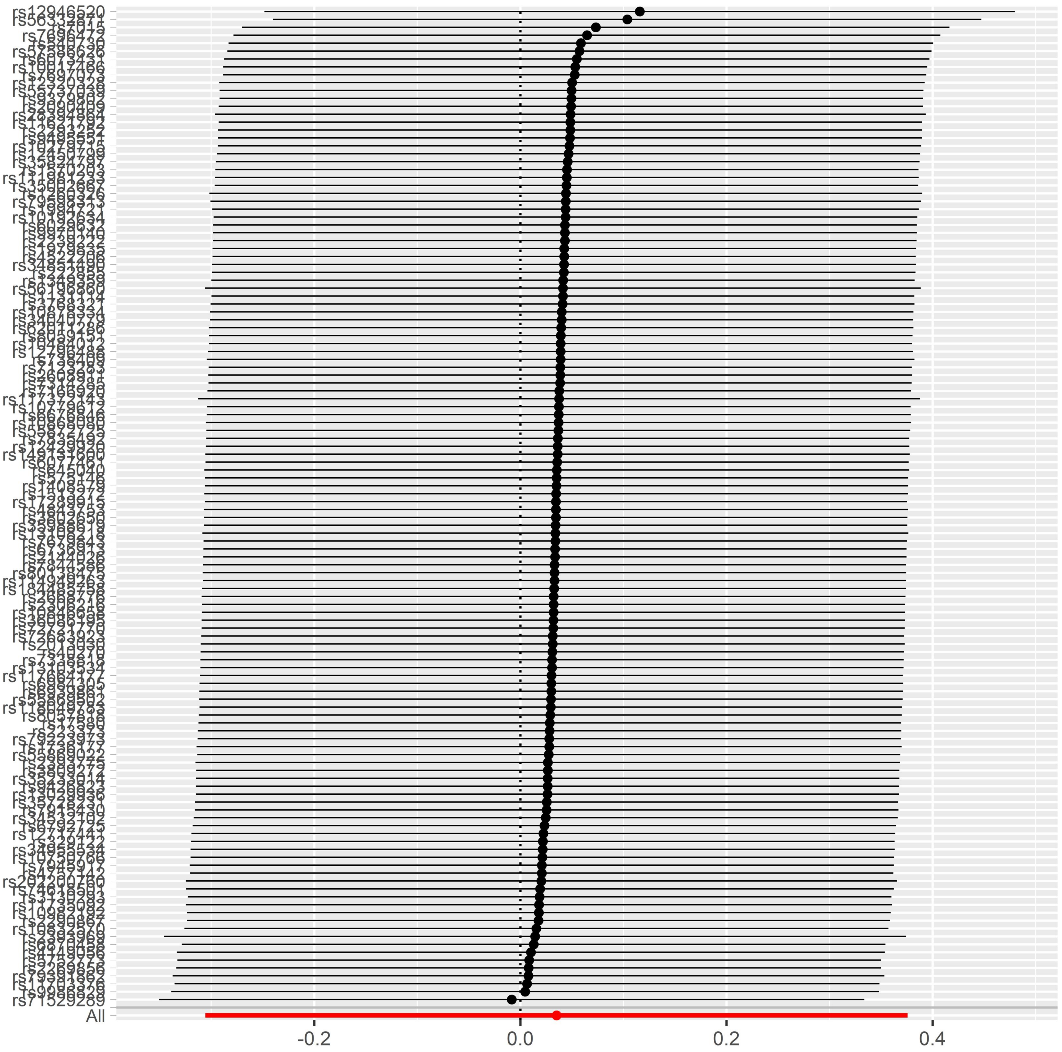

Figure S27:Leave-one-out inverse-variance weighted mendelian randomization analyses of total testosterone on Multiple myeloma and malignant plasma cell neoplasms

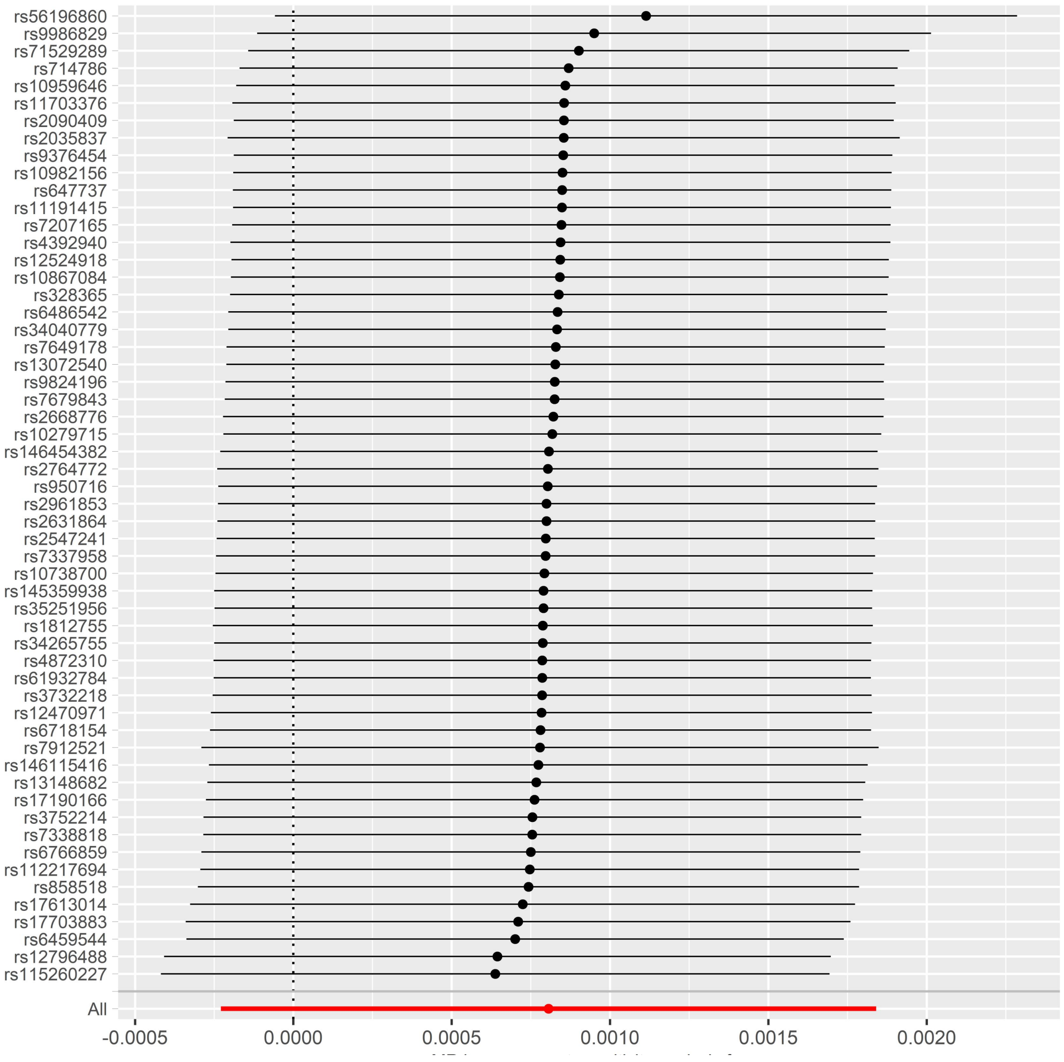

Figure S28:Leave-one-out inverse-variance weighted mendelian randomization analyses of bioavailable testosterone on Head and neck cancer

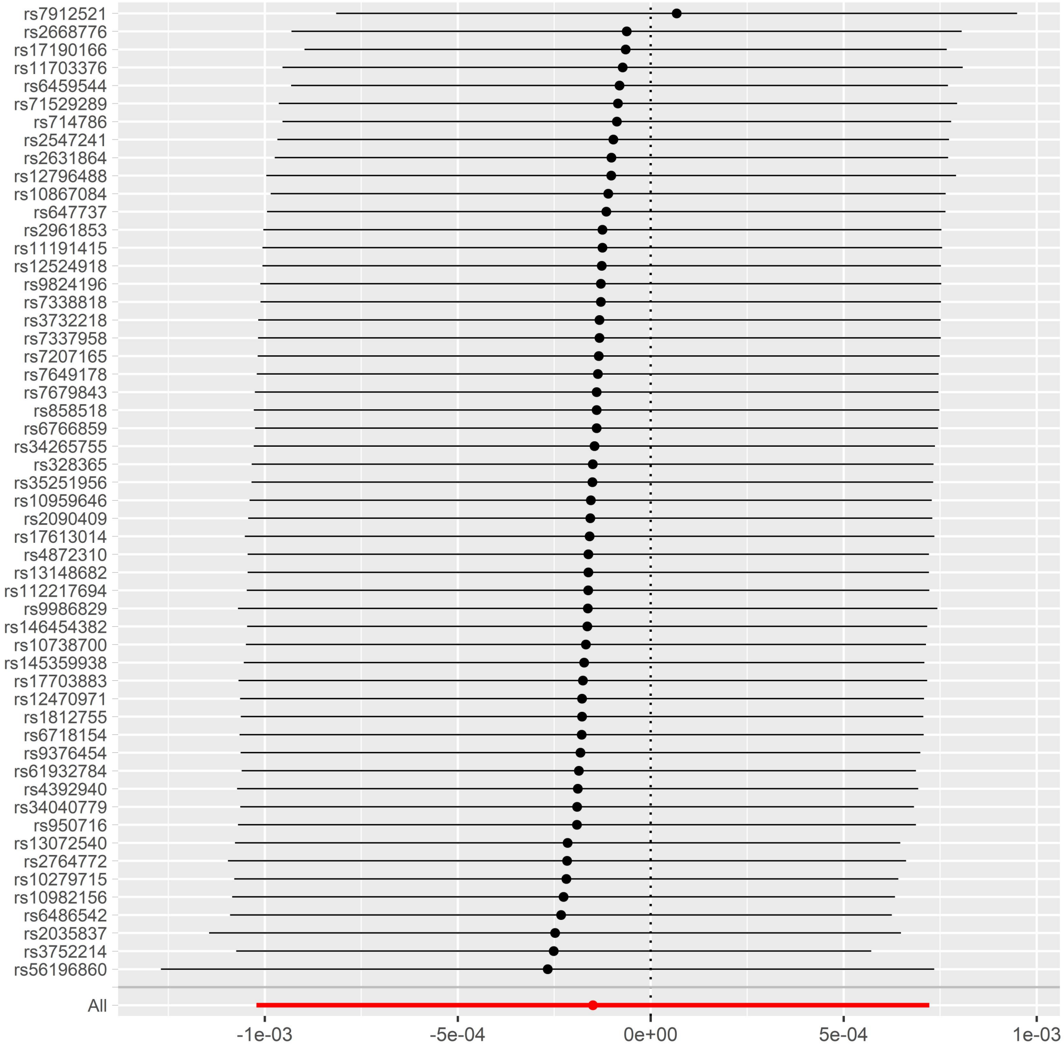

Figure S29:Leave-one-out inverse-variance weighted mendelian randomization analyses of bioavailable testosterone on Brain cancer

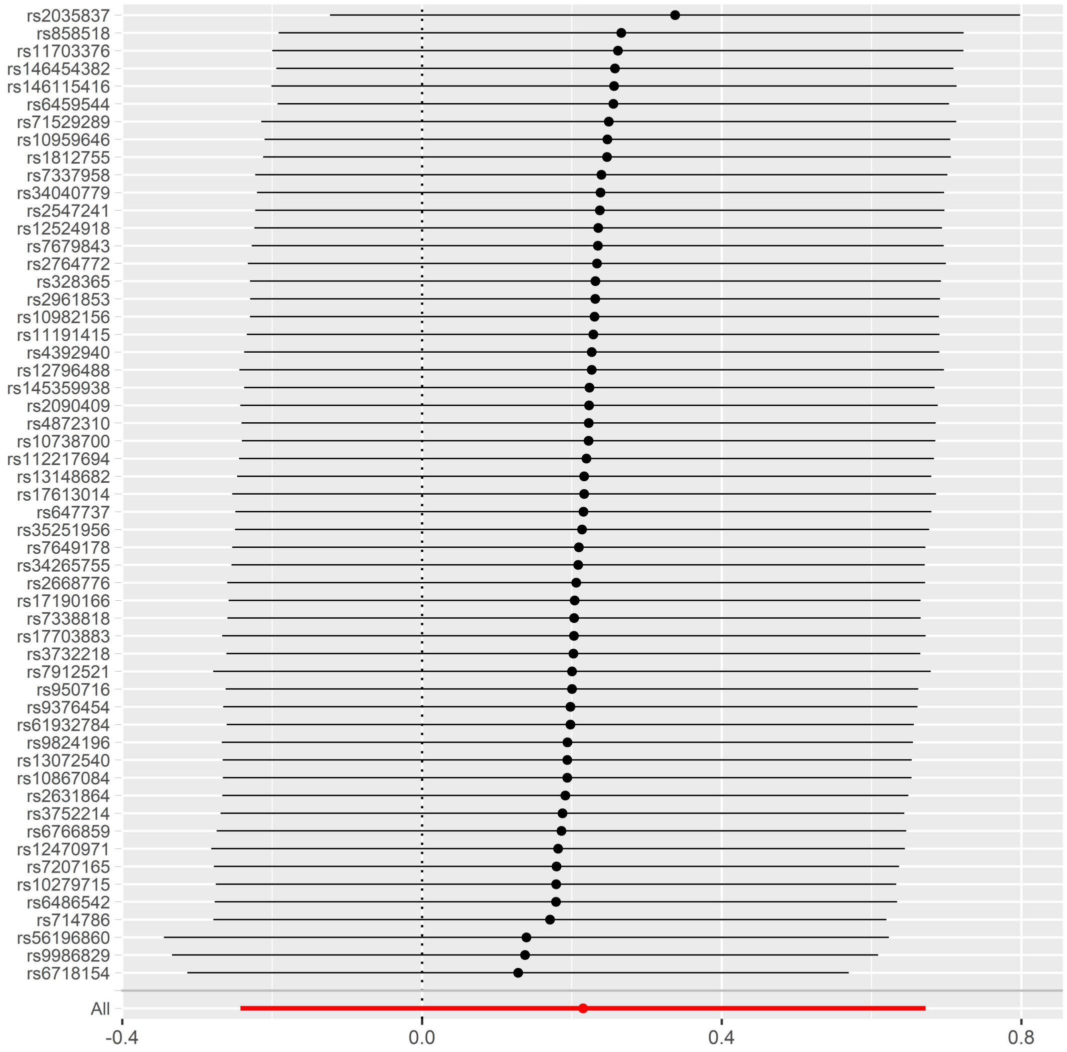

Figure S31:Leave-one-out inverse-variance weighted mendelian randomization analyses of bioavailable testosterone on Thyroid cancer

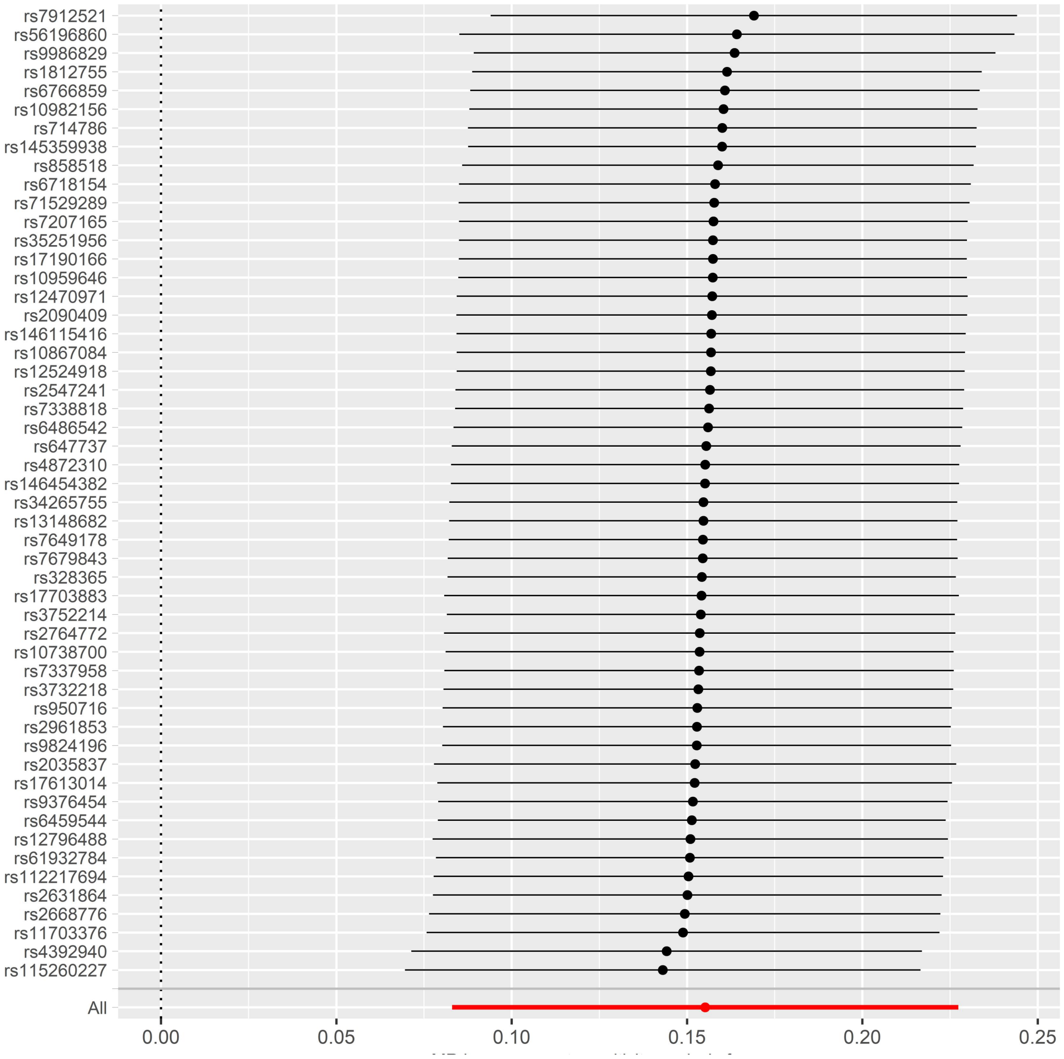

Figure S32:Leave-one-out inverse-variance weighted mendelian randomization analyses of bioavailable testosterone on Oesophageal cancer

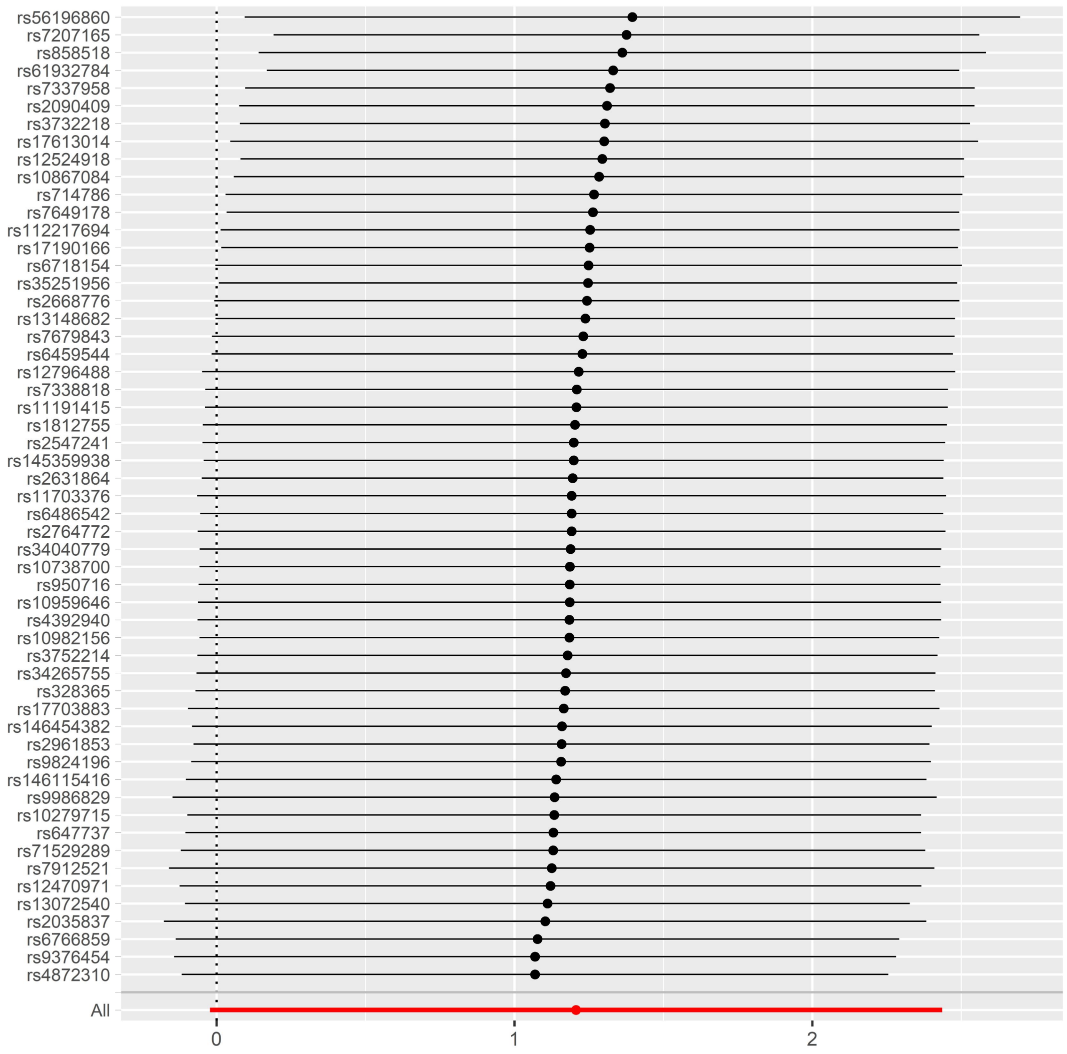

Figure S33:Leave-one-out inverse-variance weighted mendelian randomization analyses of bioavailable testosterone on Lip, oral, and pharynx cancer

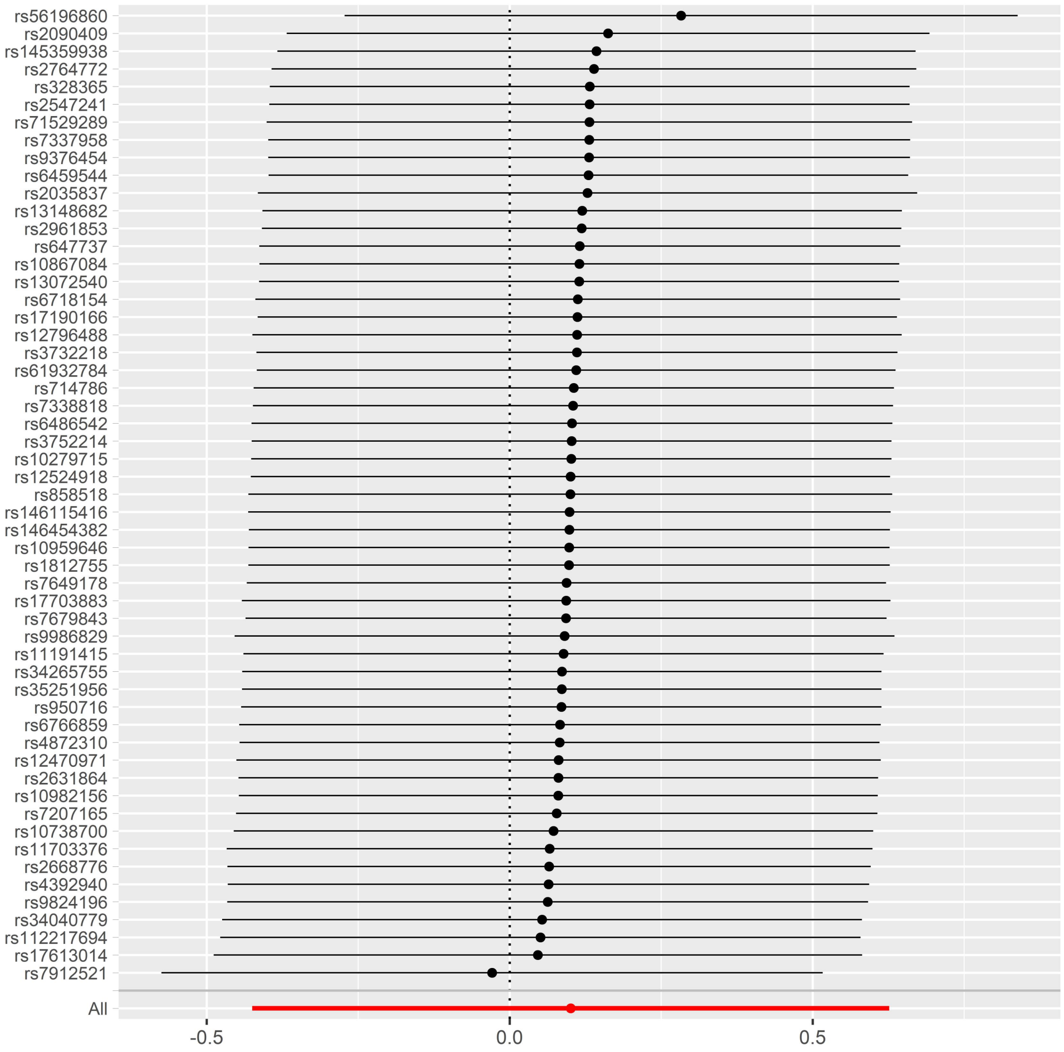

Figure S34:Leave-one-out inverse-variance weighted mendelian randomization analyses of bioavailable testosterone on Stomach cancer

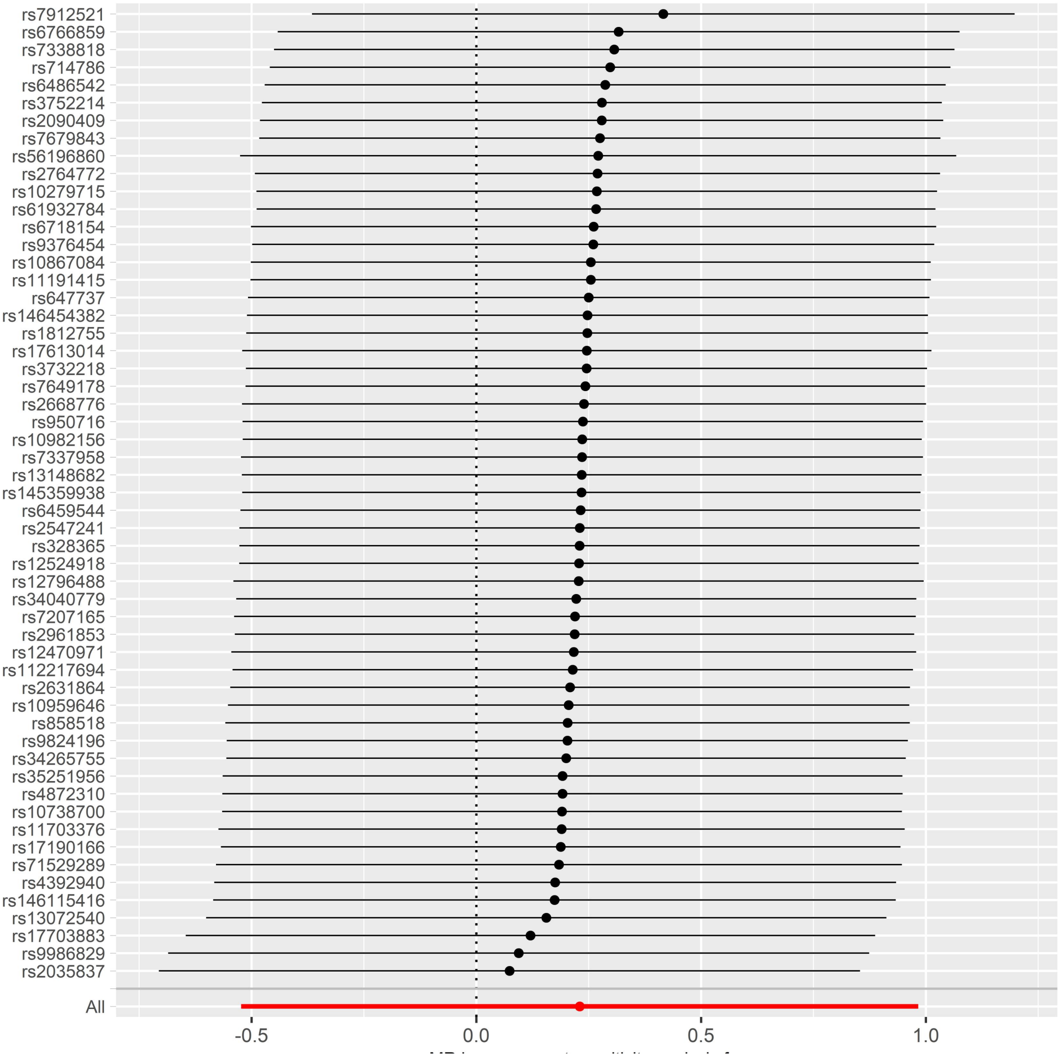

Figure S35:Leave-one-out inverse-variance weighted mendelian randomization analyses of bioavailable testosterone on Liver and intrahepatic bile ducts cancer

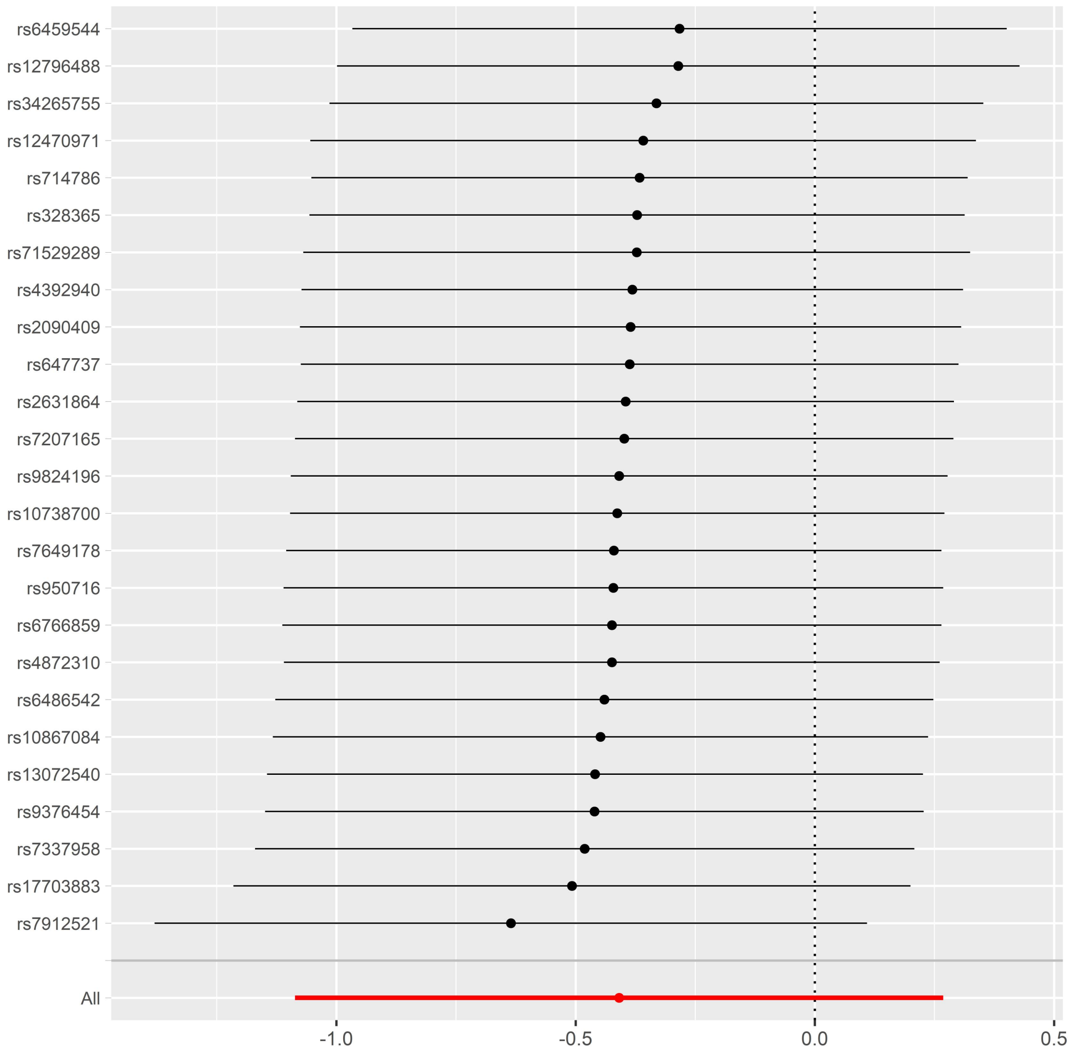

Figure S36:Leave-one-out inverse-variance weighted mendelian randomization analyses of bioavailable testosterone on Pancreatic cancer

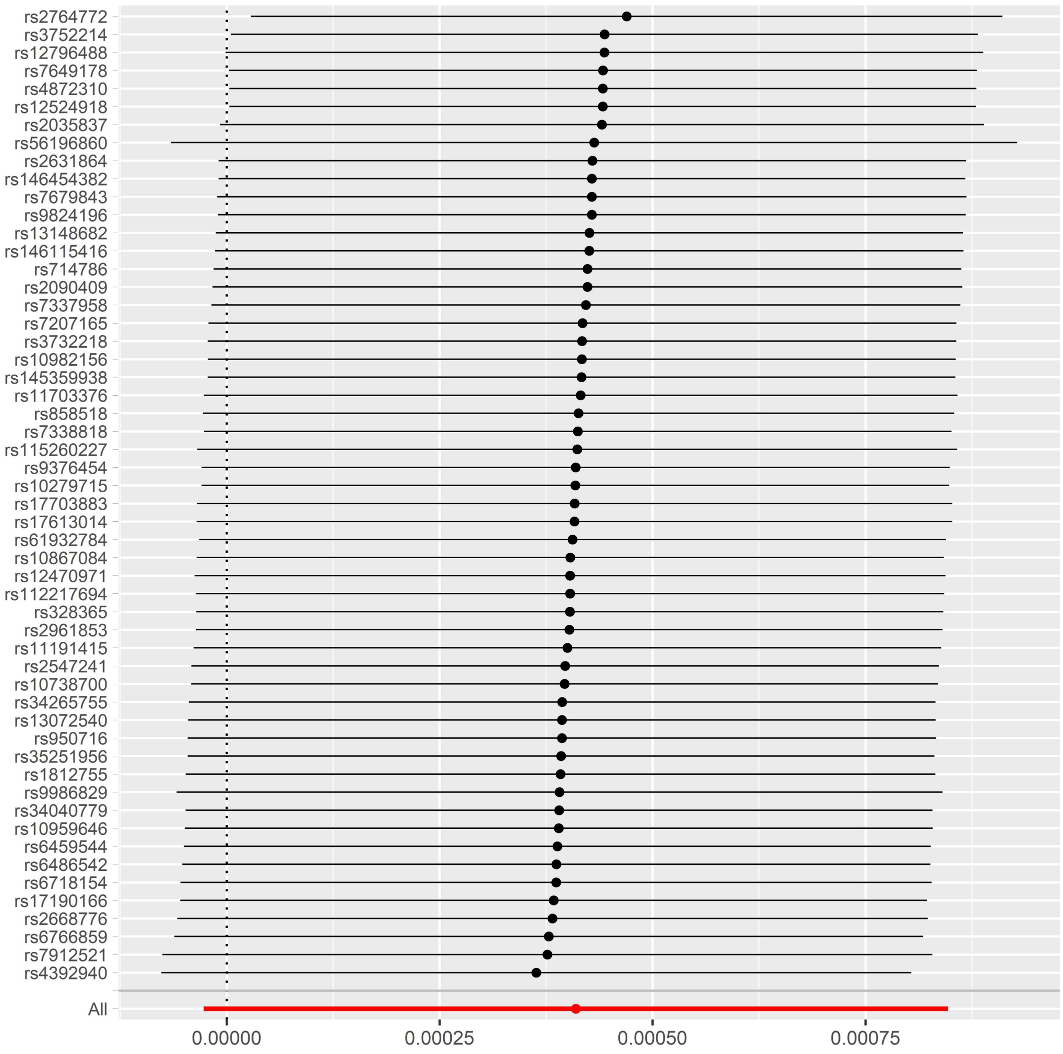

Figure S37:Leave-one-out inverse-variance weighted mendelian randomization analyses of bioavailable testosterone on Small intestine cancer

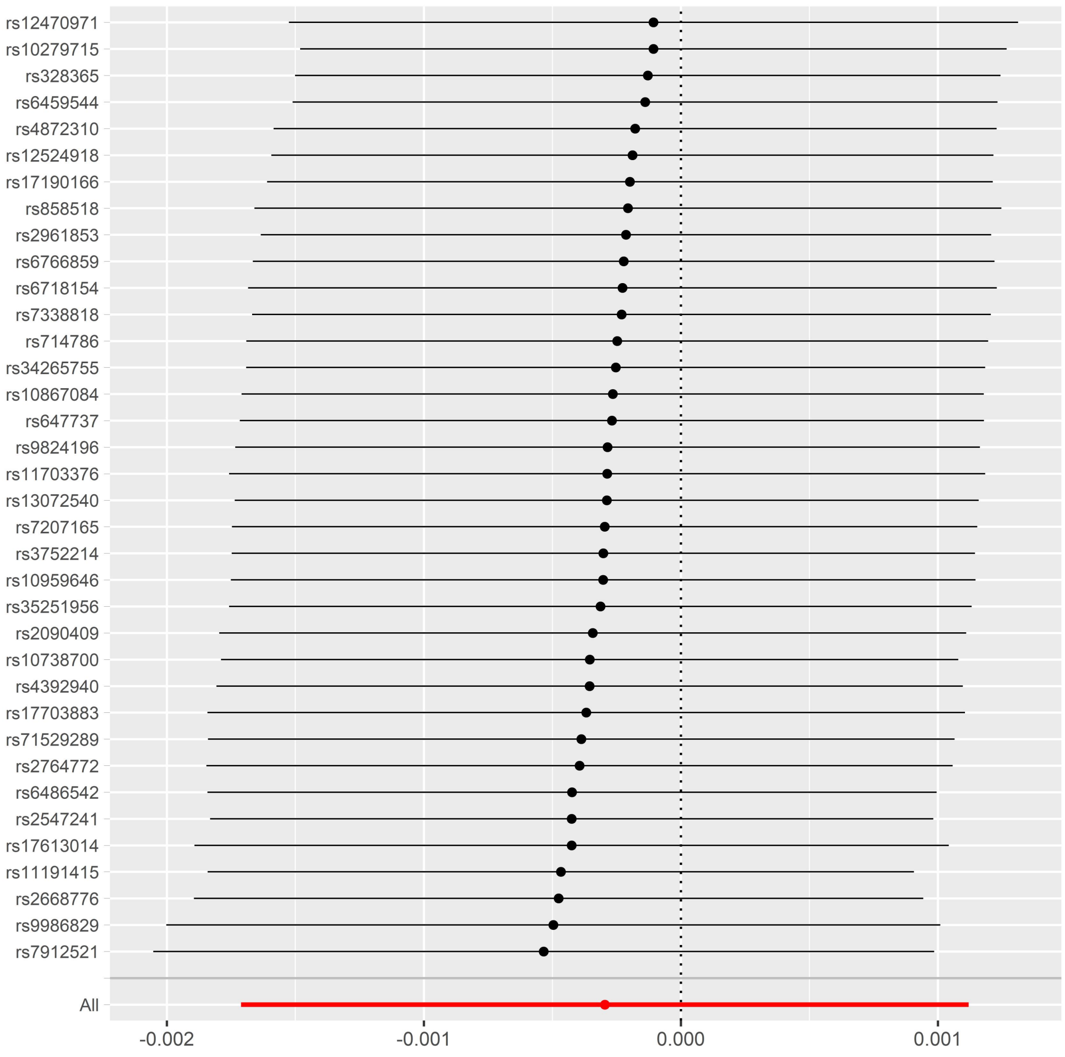

Figure S38:Leave-one-out inverse-variance weighted mendelian randomization analyses of bioavailable testosterone on Colon cancer

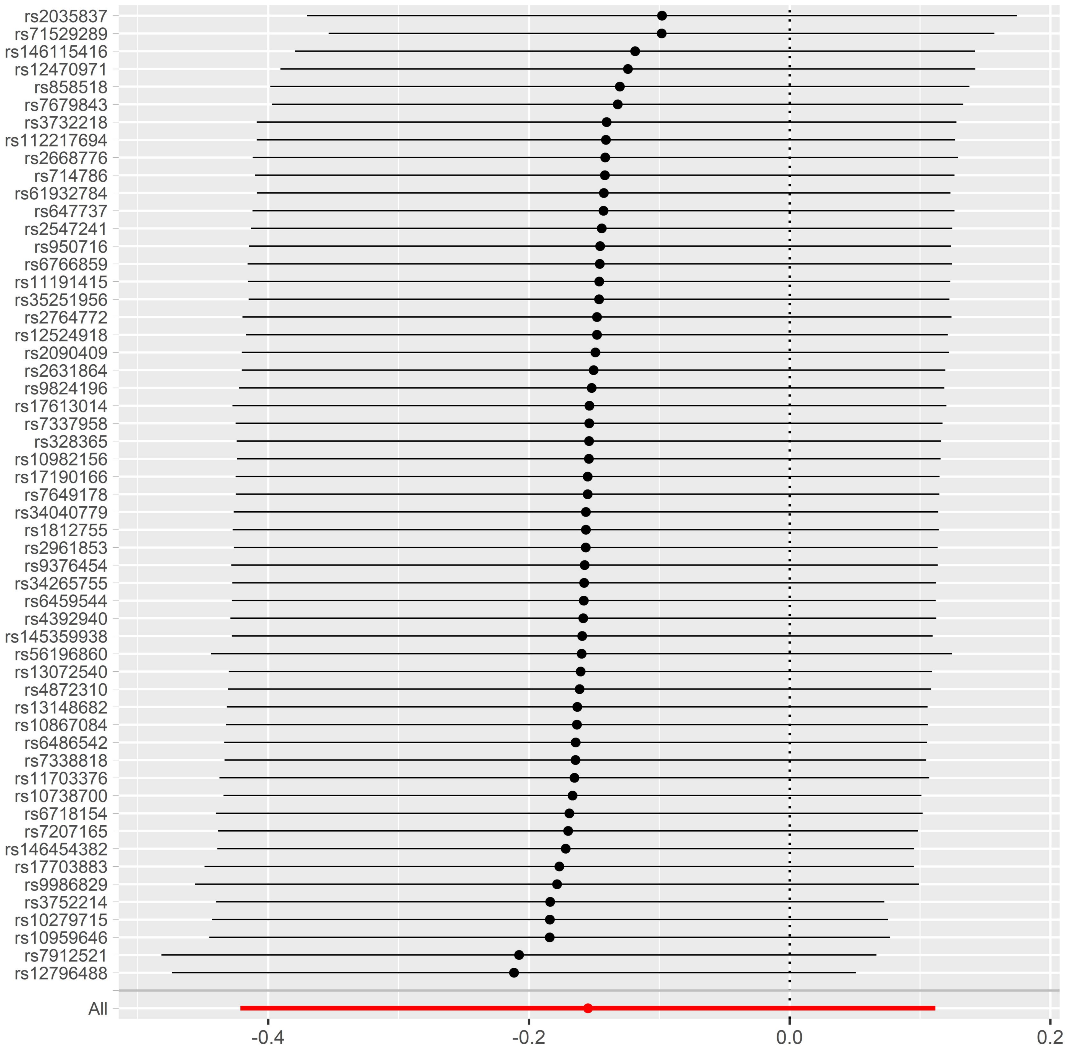

Figure S39:Leave-one-out inverse-variance weighted mendelian randomization analyses of bioavailable testosterone on Colorectal cancer

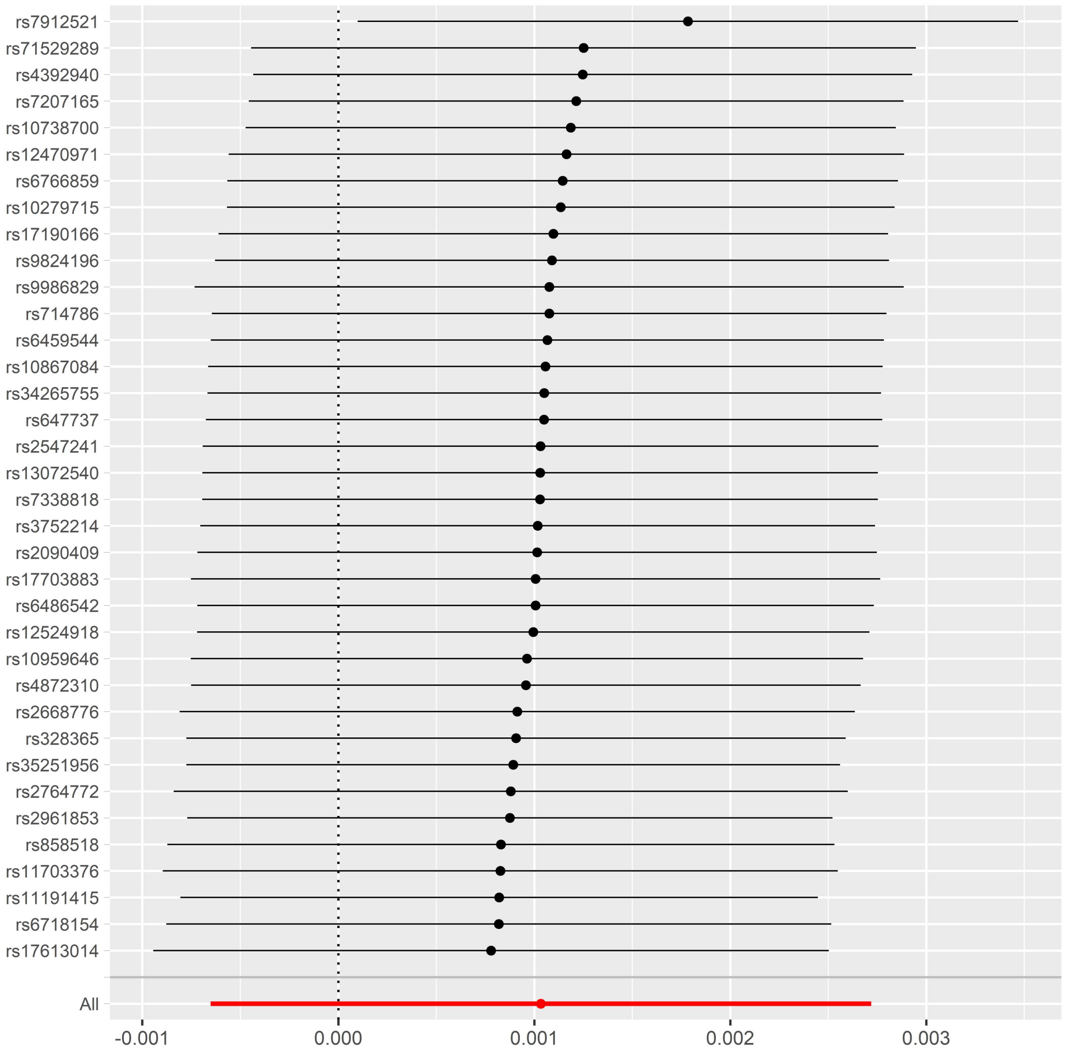

Figure S40:Leave-one-out inverse-variance weighted mendelian randomization analyses of bioavailable testosterone on Rectum cancer

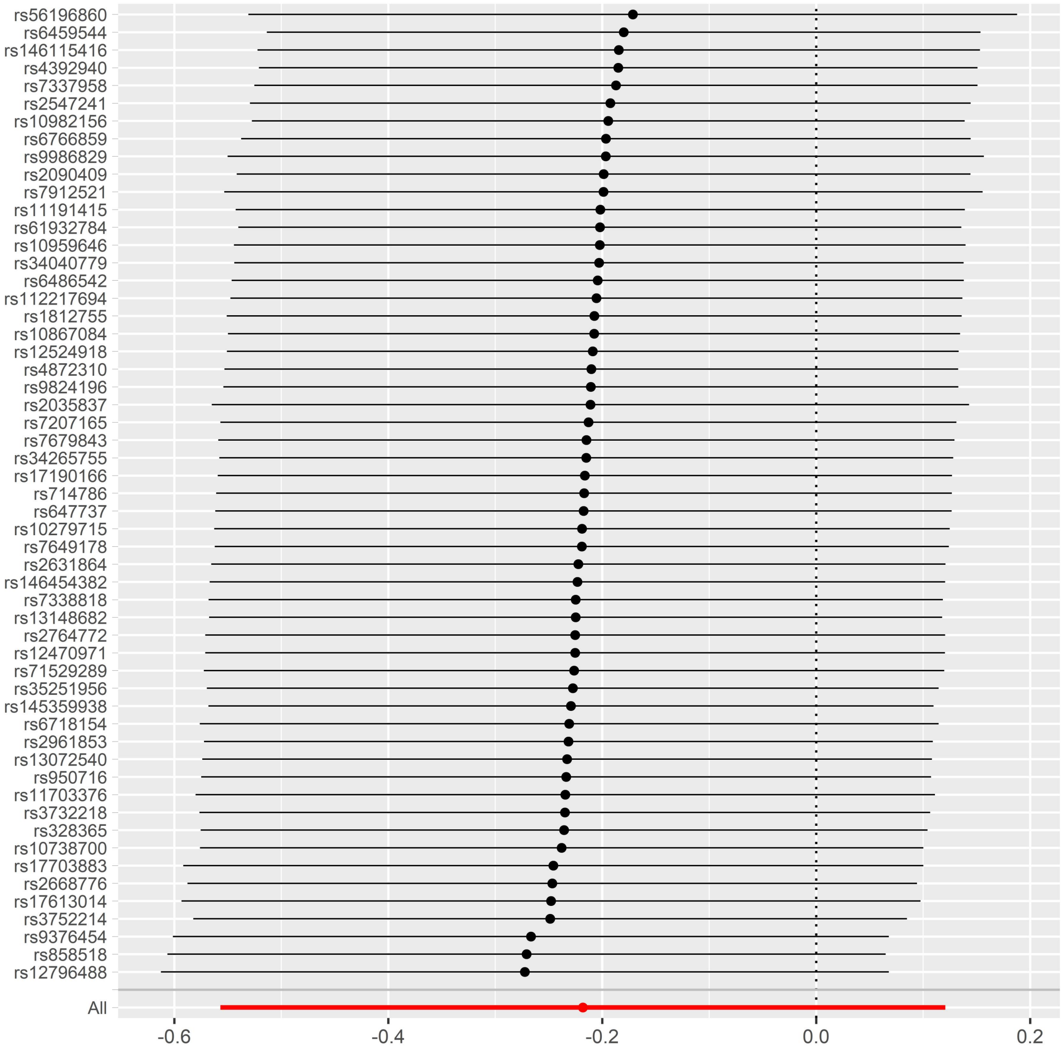

Figure S41:Leave-one-out inverse-variance weighted mendelian randomization analyses of bioavailable testosterone on Non-small cell lung cancer

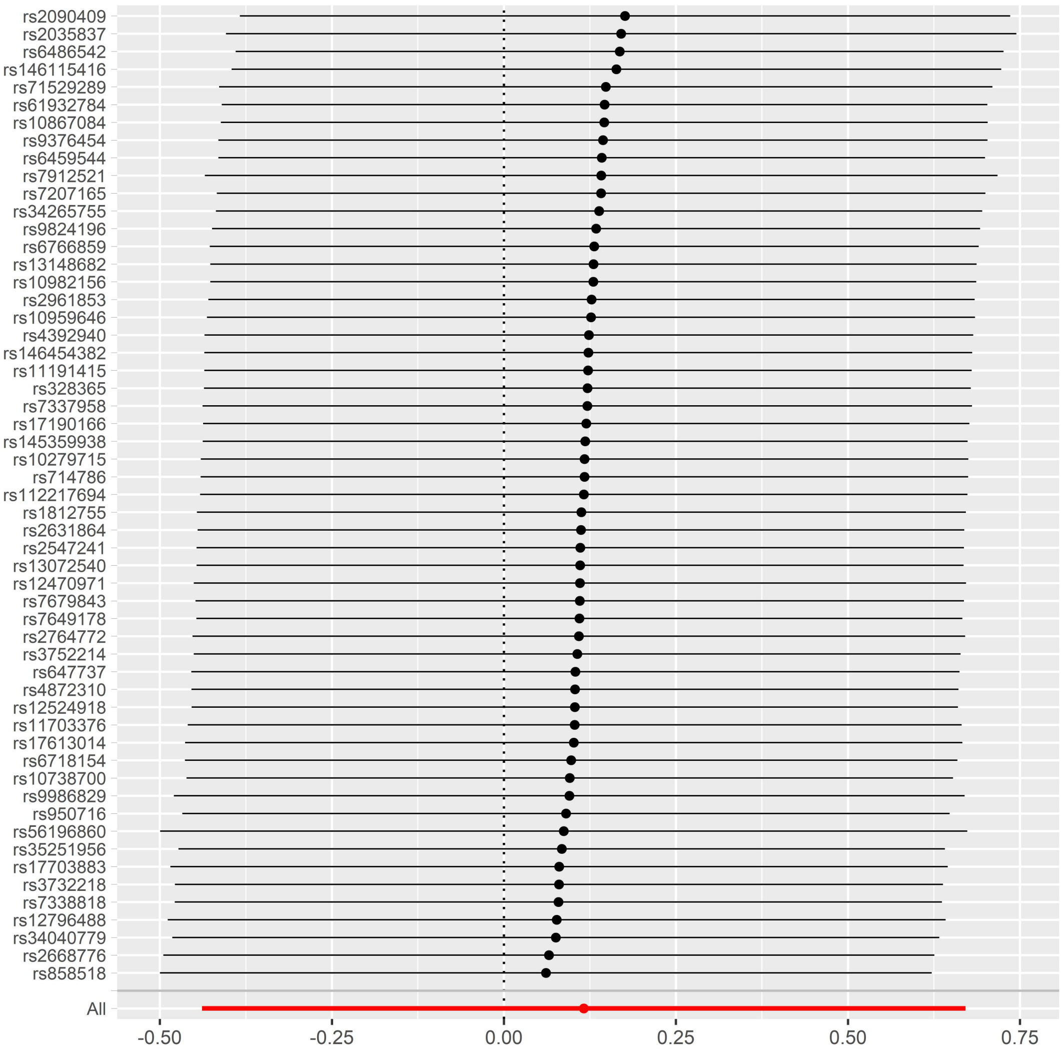

Figure S42:Leave-one-out inverse-variance weighted mendelian randomization analyses of bioavailable testosterone on Adenocarcinoma cell lung cancer

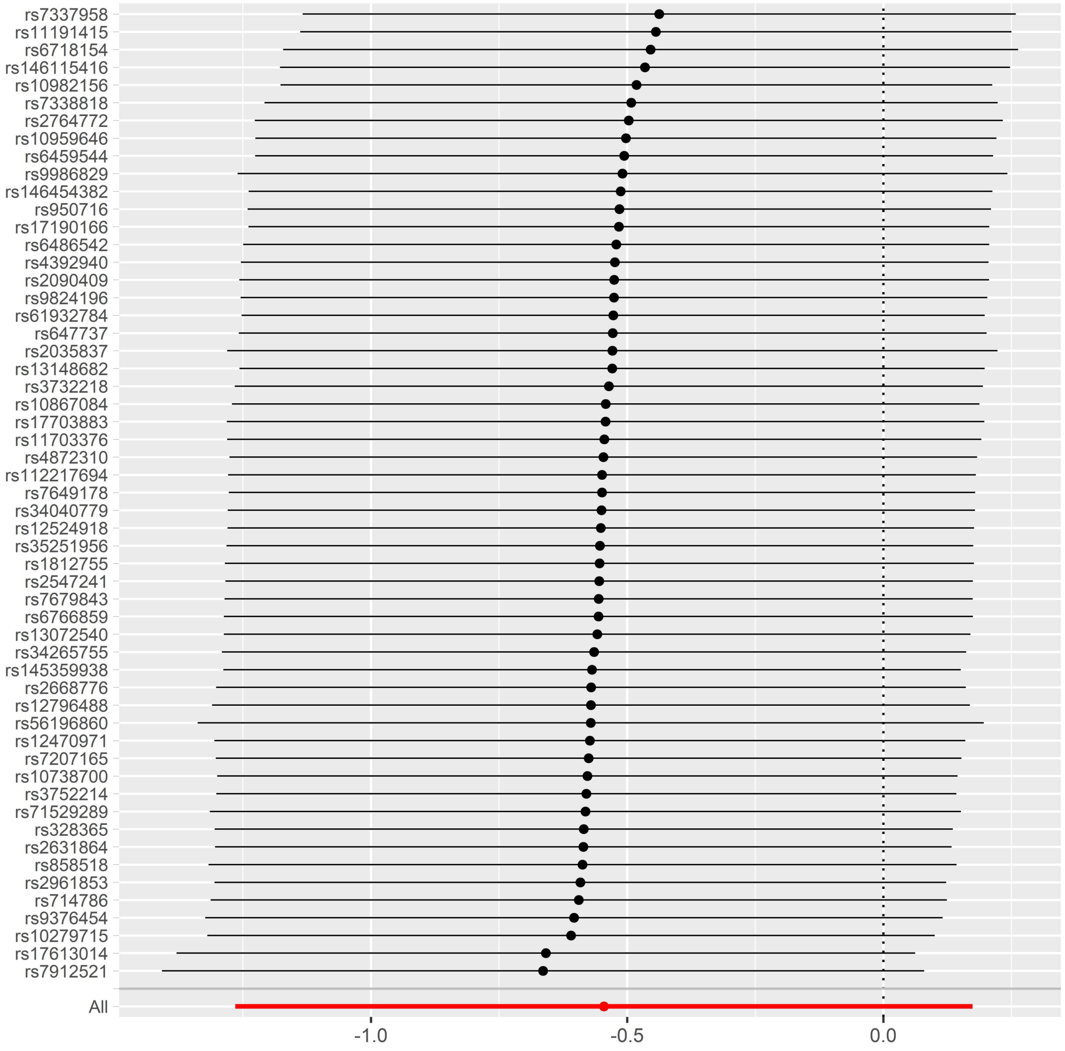

Figure S43:Leave-one-out inverse-variance weighted mendelian randomization analyses of bioavailable testosterone on Squamous cell lung cancer

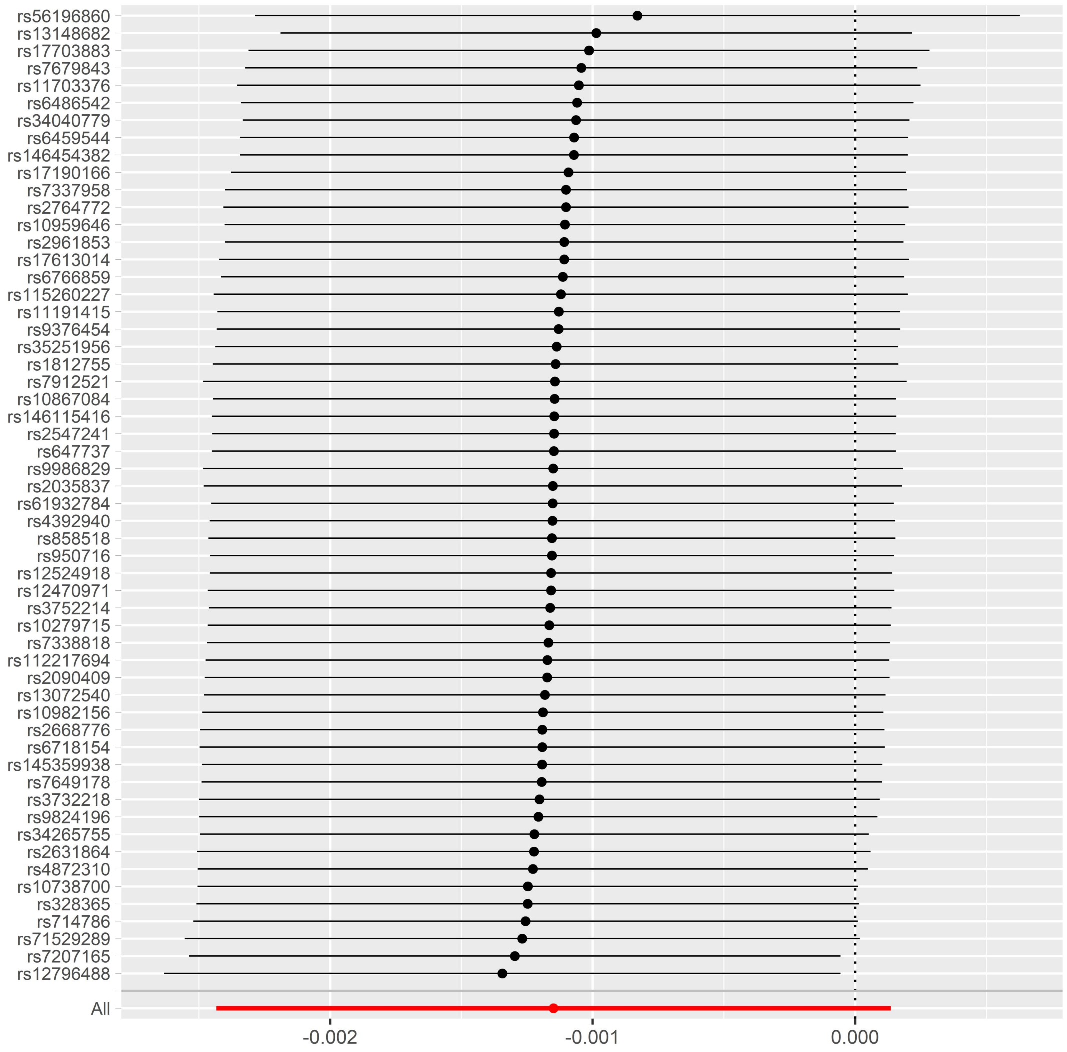

Figure S44:Leave-one-out inverse-variance weighted mendelian randomization analyses of bioavailable testosterone on Bladder cancer

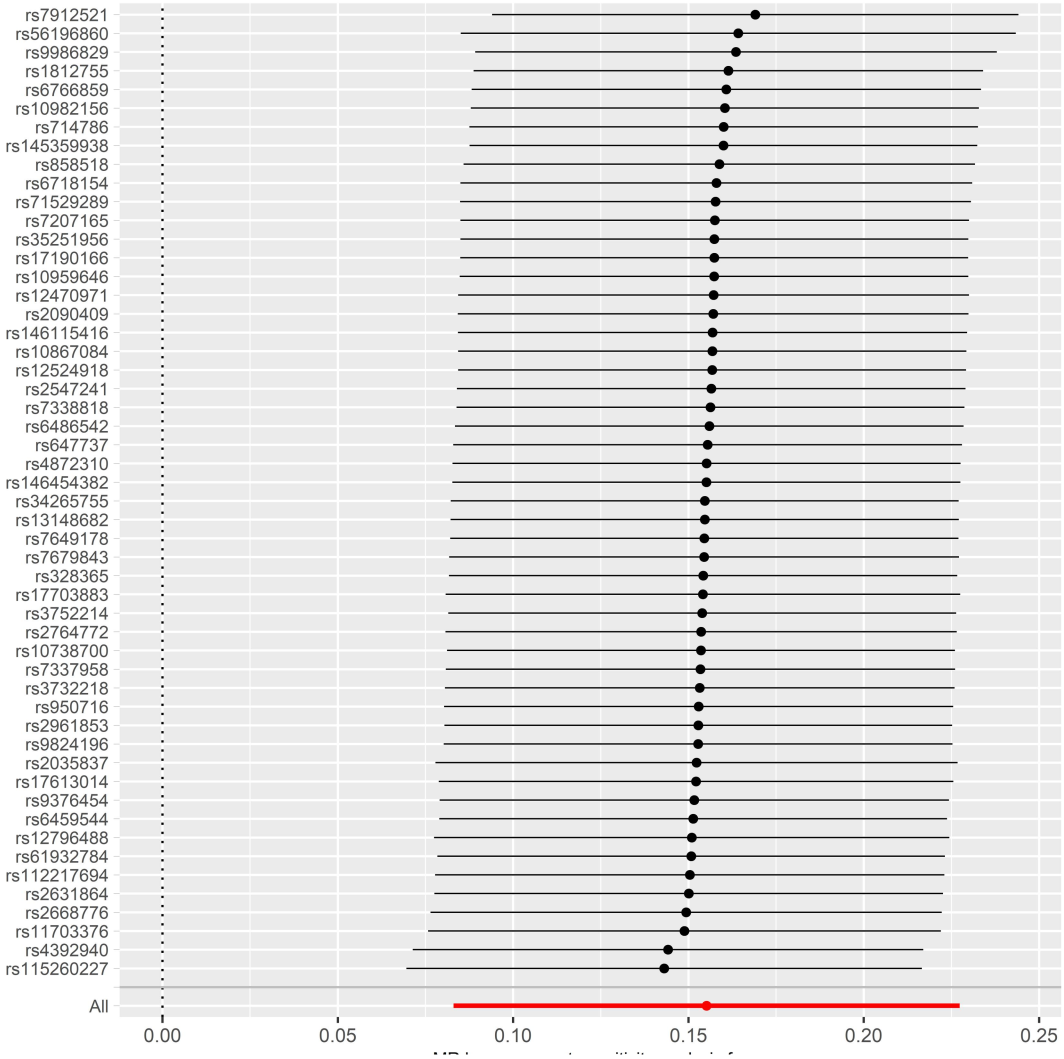

Figure S45:Leave-one-out inverse-variance weighted mendelian randomization analyses of bioavailable testosterone on Prostate cancer

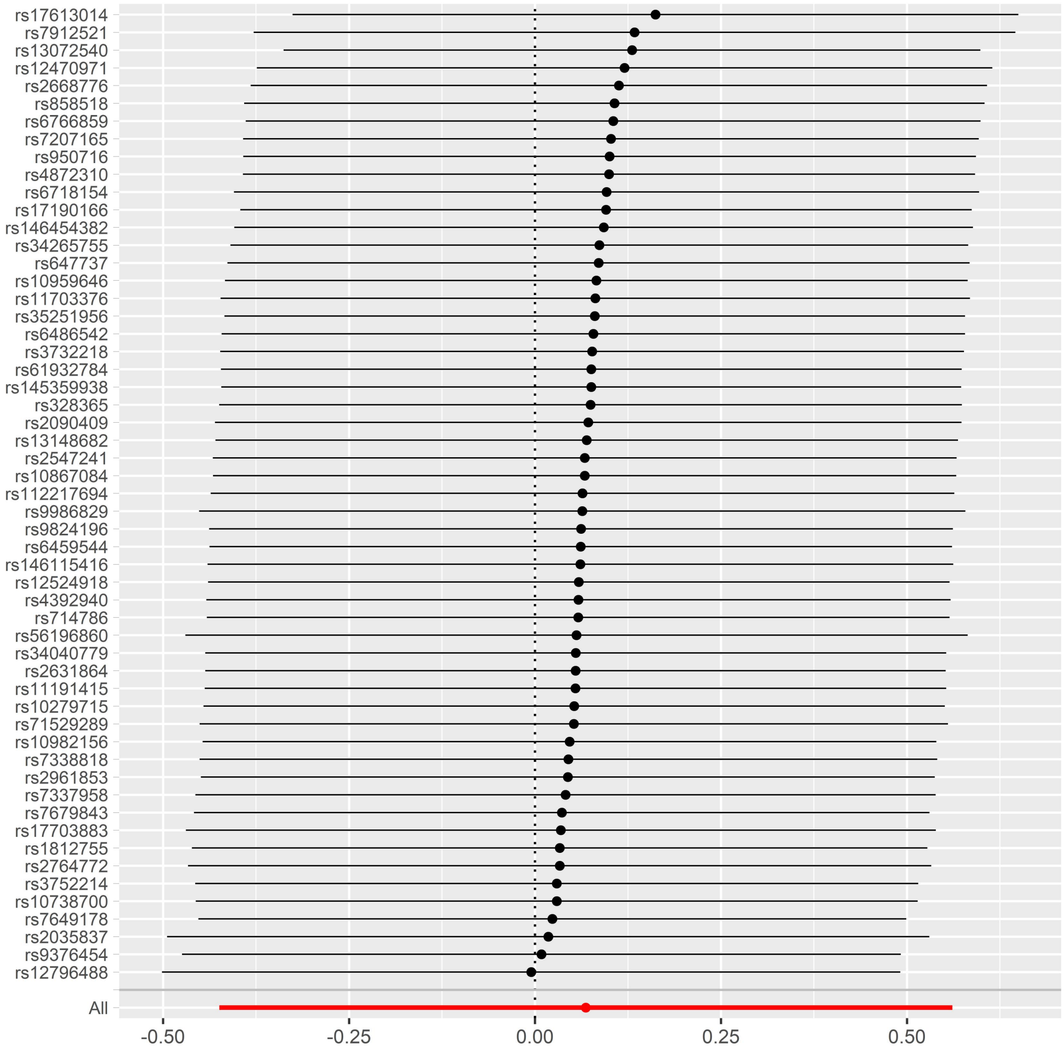

Figure S46:Leave-one-out inverse-variance weighted mendelian randomization analyses of bioavailable testosterone on Kidney cancer

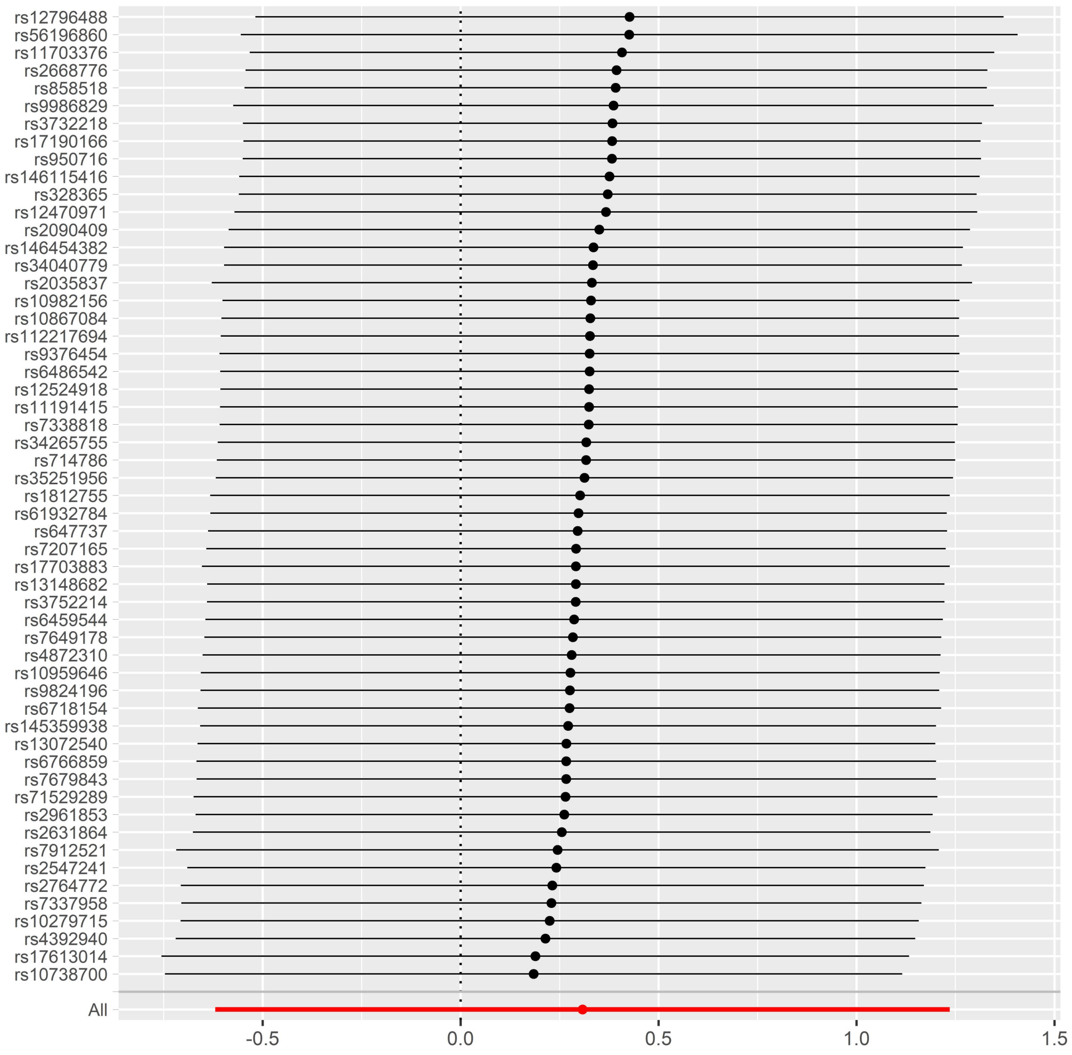

Figure S47:Leave-one-out inverse-variance weighted mendelian randomization analyses of bioavailable testosterone on Testis cancer

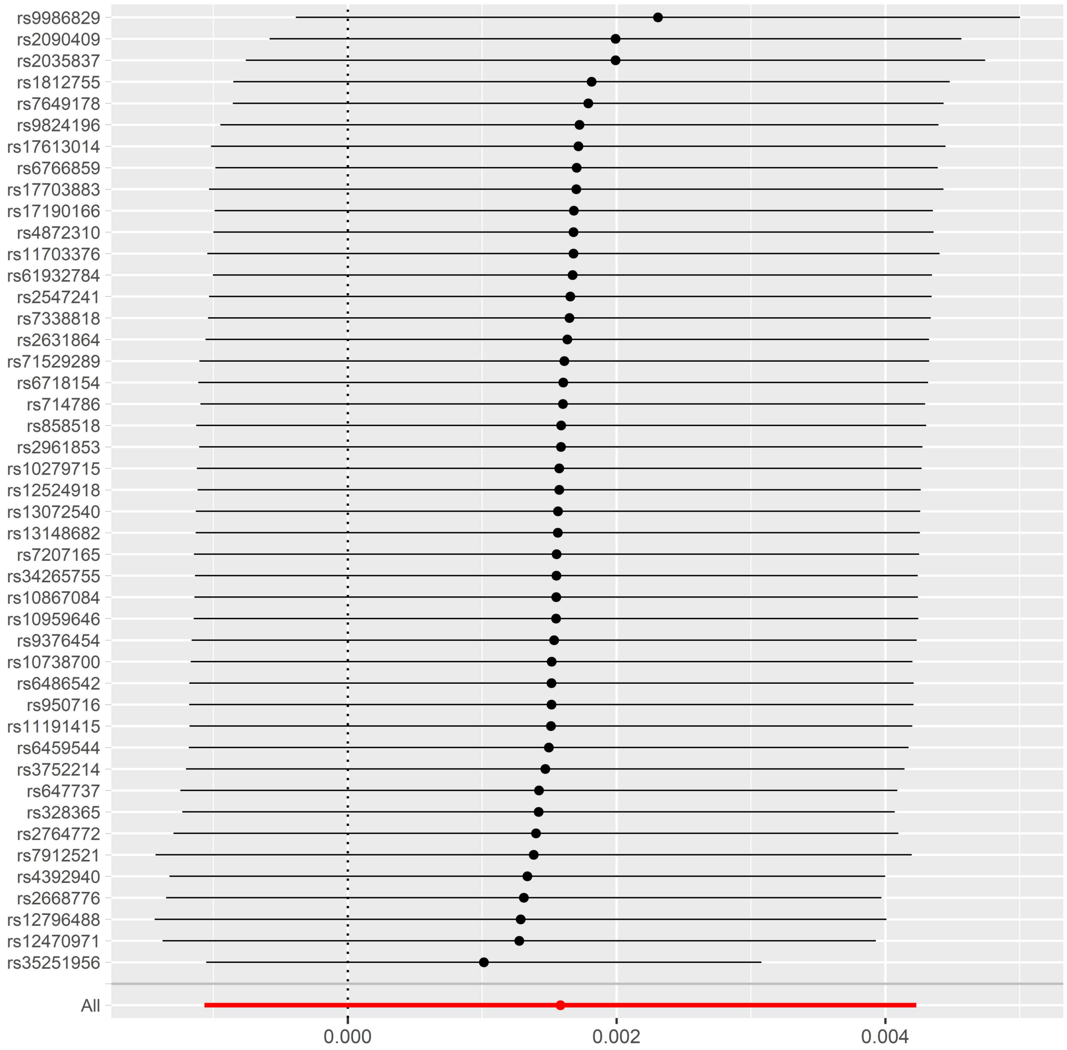

Figure S48:Leave-one-out inverse-variance weighted mendelian randomization analyses of bioavailable testosterone on Malignant melanoma

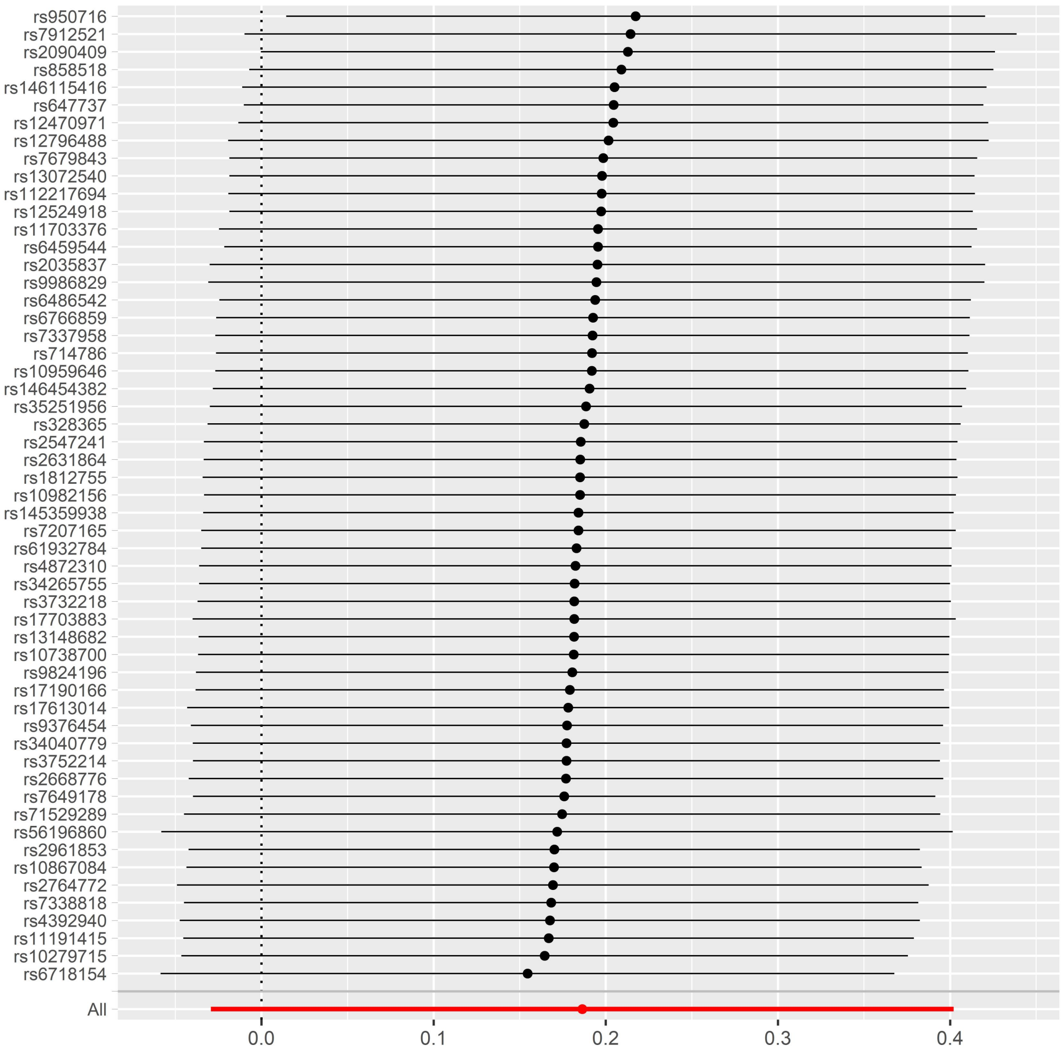

Figure S49:Leave-one-out inverse-variance weighted mendelian randomization analyses of bioavailable testosterone on Malignant neoplasm of male genital organs

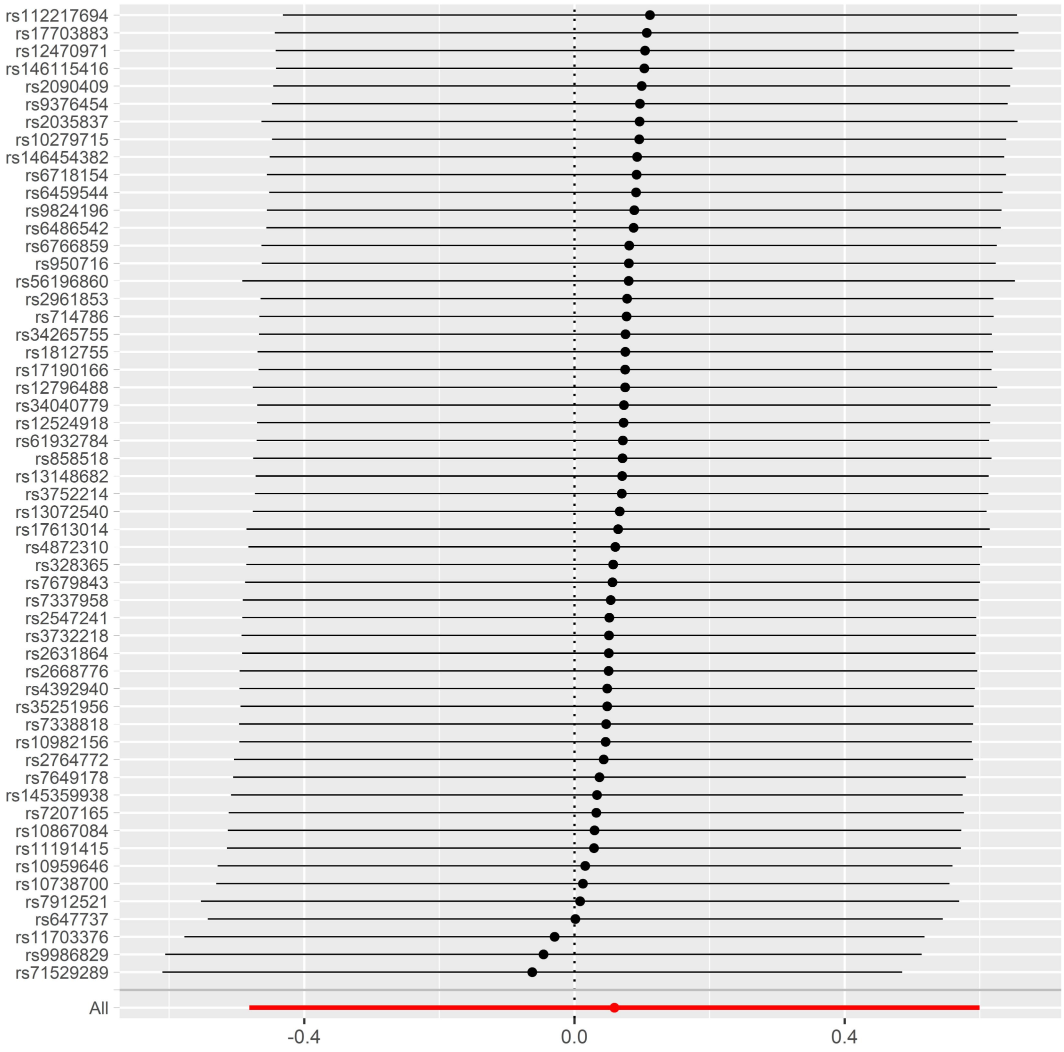

Figure S50:Leave-one-out inverse-variance weighted mendelian randomization analyses of bioavailable testosterone on Multiple myeloma and malignant plasma cell neoplasms
